# Beat- and Side-family cell-surface molecules are expressed combinatorially in the partner neurons of the olfactory circuit in *Drosophila*

**DOI:** 10.1371/journal.pbio.3003955

**Published:** 2026-08-27

**Authors:** Qichen Duan, Sumie Okuwa, Rachel Estrella, Chun Yeung, Yu-Chieh David Chen, Laura Quintana Rio, Chengcheng Du, Khanh M. Vien, Pelin Cayirlioglu Volkan

**Affiliations:** 1 Department of Biology, Duke University, Durham, North Carolina, United States of America; 2 Department of Biology, New York University, New York, New York, United States of America; 3 Department of Biochemistry and Molecular Biophysics, Columbia University, New York, New York, United States of America; University of Michigan, UNITED STATES OF AMERICA

## Abstract

Over the past decades, many molecular players have been uncovered to control distinct steps of olfactory circuit assembly in *Drosophila*. Among these, multi-member gene families encoding cell-surface proteins are of interest as they can act as neuron-specific recognition tags in combinations and contribute to circuit assembly in complex brains. Recently, a multi-protein interactome has been described between Beat and Side families of IgSF proteins. Here, we use newly generated gene trap transgenic driver lines to probe the spatial expression pattern of *beat/side* genes in olfactory receptor neurons (ORNs) and their synaptic target projection neurons (PNs)*.* Our results revealed that each ORN/PN class expresses a specific combination of *beat/side* genes, hierarchically regulated by lineage-specific genetic programs. To explore whether the class-specific expression of *beats/sides* defines ORN-PN matching specificity, we perturbed presynaptic *beat-IIa* and postsynaptic *side-IV* in two ORN-PN partners. However, disruption of Beat-IIa-Side-IV interaction did not produce any significant mistargeting in these two examined glomeruli. Our expression mapping revealed that the Beat/Side interactome between ORNs and PNs appears to be error-tolerant, supporting the robust *trans-*synaptic recognition. Though without affecting general glomerular targeting, knockdown of *side* in ORNs leads to the reduction of synaptic development. Interestingly, we found conserved expression patterns of *beat/side* orthologs across ORNs in ants and mosquitoes, indicating the shared regulatory strategies specifying the expression of these duplicated paralogs in insect evolution. This also implies the biological significance of *beats/sides* in ORN circuit development or function, which is preserved under selective pressure across divergent insect lineages. Overall, this comprehensive analysis of expression patterns lays a foundation for in-depth functional investigations into how Beat/Side combinatorial expression contributes to the olfactory circuit assembly.

## Abstract 2

Multi-member gene families encoding cell-surface proteins can contribute to the assembly of complex brain circuits. This study in Drosophila shows two such conserved gene families are expressed combinatorially in fly olfactory circuits, suggesting that they may act as neuron-specific recognition tags to support brain patterning.

## Introduction

The *Drosophila* olfactory system provides an excellent model for understanding the genetic basis of the neuronal class-specific circuit organization and assembly. The first-order sensory neurons in the circuit are olfactory receptor neurons (ORNs), which are housed in the peripheral sensilla covering the antennae [1–5]. Each ORN typically expresses only a single identity-defining chemosensory receptor gene—such as an olfactory receptor (OR), ionotropic receptor (IR), or gustatory receptor (GR) gene—or, in some cases, a unique combination of up to three receptor genes [2]. The cell bodies of ORNs expressing the same chemosensory receptor genes, thus of the same class, are dispersed across the antenna. Yet, their axons converge onto a single, uniquely positioned class-specific glomerulus in the antennal lobe, where they synapse with the second-order projection neurons (PNs) [1,2]. ORNs are thus defined by their chemosensory receptor expression and glomerular target identity, comprising ~60 classes [6]. PNs, born from distinct neuroblast lineages surrounding antennal lobes, are also defined by their unique dendritic targeting of the dedicated glomeruli. At the same time, their axons extend to other higher brain regions, mushroom bodies, and lateral horns, which integrate the odor signal with other sensory cues and internal states to guide the animals’ behaviors [1,7–9]. Importantly, within the antennal lobe, ORNs and PNs form a one-to-one match in the class-specific glomerulus. This precisely controlled organization brings many interesting questions. How are these neurons born? What molecules mediate the communication between the neurons of the same class, different classes, or pre- and post-synaptic partners? How do these neurons acquire the necessary cell-surface signaling to mediate these interactions that eventually ensure the formation of this stereotyped glomerular map? As this one-to-one structure maintains over evolution from insects to mammals [10], the easy and genetically tractable *Drosophila* is a great platform to determine the lineage-specific and combinatorial expression of cell-surface proteins, their establishment, function, and evolution.

Many molecular players have been identified to participate in organizing the *Drosophila* olfactory circuits, ranging from the critical regulatory hubs at the top of the developmental hierarchy, like transcription factors instructing the lineage and/or the expression of a broad range of cell-surface molecules (CSMs) [11–15], to the executors at the bottom of the hierarchy, which are critical CSMs themselves. These include many members of large protein families, like leucine-rich repeat superfamily members Toll-6/Toll-7 [16], Fili [17], and Capricious [18]; Teneurins Ten-a and Ten-m [19,20]; Cadherin superfamily members Ncad [21,22], Flamingo [23], and Fat2 (Kug) [24]; Immunoglobulin Super Family (IgSF) members Dscam with extremely diverse isoform repertoire [25–28] and DIPs/Dprs, which consist of multiple paralogs [29]. Our previous work showed that class-specific combinatorial expression of DIPs/Dprs organizes ORN axons within glomeruli [29]. DIPs/Dprs are of particular interest because they form two multi-member subfamilies (11 DIPs and 21 Dprs), bind one another (Dprs to DIPs) primarily through heterophilic interactions, and exhibit striking cell-type-specific expression [30–36]. Besides the olfactory system, several *trans-*synaptically interacting DIPs and Dprs have been shown to control synapse selectivity and formation in the visual circuits and neuromuscular junction [31,37–45].

**Fig 1:**
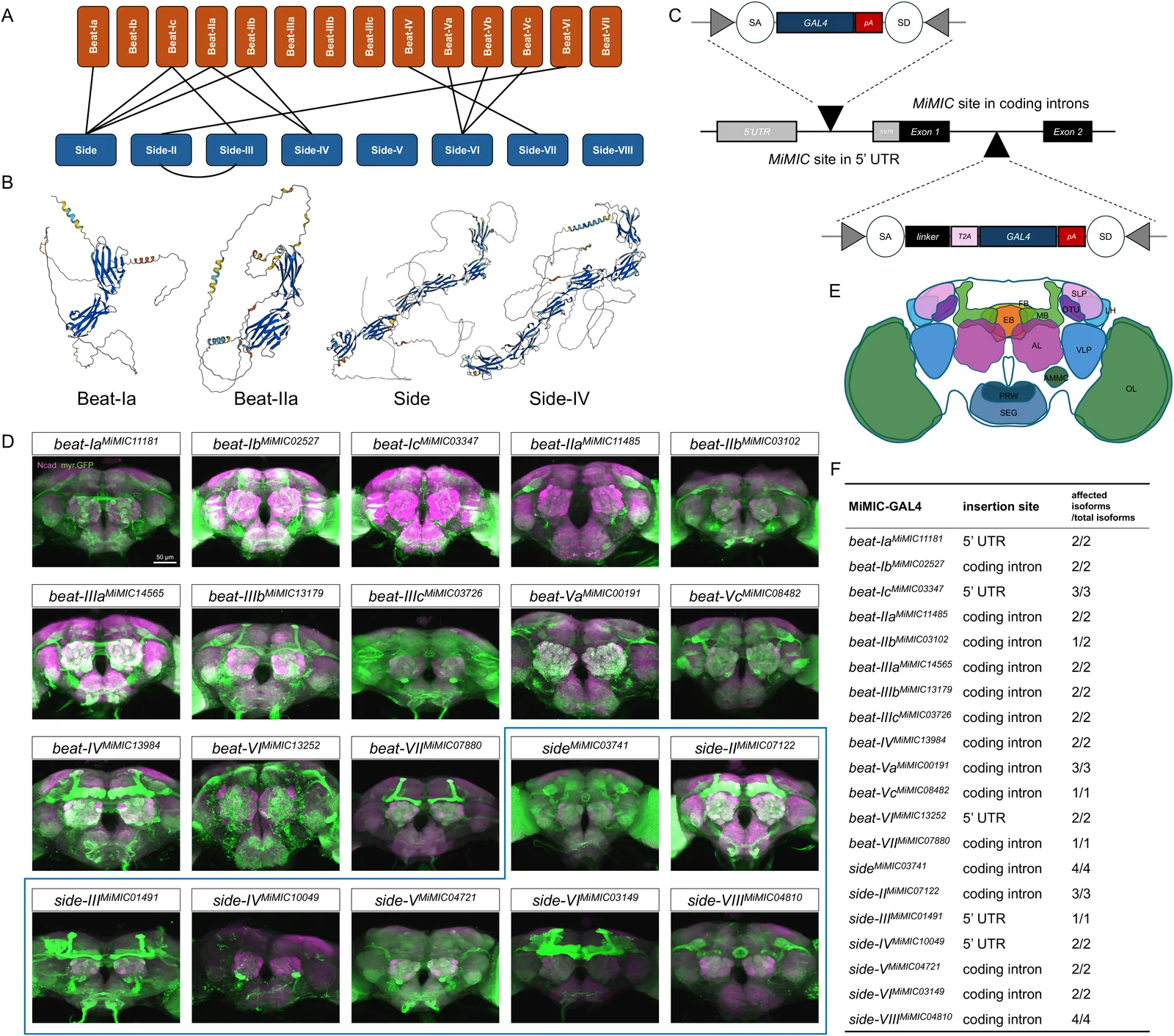
Beats/Sides are heterophilically interacting IgSF proteins. **(A)** Schematics showing the known interactions between Beat and Side proteins. **(B)** AlphaFold-predicted structures of example Beat and Side proteins. Beats have two extracellular Ig domains, while Sides’ extracellular parts consist of five Ig domains and one Fibronectin type III domain. Transmembrane and intracellular domains are poorly predicted. Structures were downloaded from AlphaFold.ebi.ac.uk [61]. **(C)** Schematic showing *MiMIC*-based insertion of the GAL4-coding construct into either the 5′ UTR or the “coding” intron, which will hijack the expression of the host gene. If *GAL4* is inserted into introns within the 5′ UTR, the *GAL4* artificial exon will first be kept from splicing and then will be translated as it has its own start codon. If the in-frame *T2A-GAL4* construct is inserted into an intron between two coding exons, this whole artificial exon will also be prevented from splicing and then translated along with the upstream coding sequence. During translation, the *T2A* sequence causes the ribosome to fail at synthesizing the peptide bond, and thereby, a truncated peptide of the host gene and a GAL4 protein will be produced separately. In both cases, GAL4 is expected to be expressed in the same cell where the native gene is expressed. SA, splice acceptor; SD, splice donor; pA, Hsp70 polyadenylation signal to terminate transcription; linker sequence ensures *T2A-GAL4* will be translated in frame. **(D)** Expression pattern of each *beat/side* in the brain revealed by *beat/side-MiMIC-GAL4*. Green is the GFP signal driven by the gene trap GAL4, and magenta is anti-Ncad staining showing the brain architecture. **(E)** Schematic showing several major anatomical neuropils of the fruit fly brain. AL, antennal lobe; AMMC, antenna-mechanosensory and motor center; FB, fan-shaped body; EB, ellipsoid body; MB, mushroom body; LH, lateral horn; SLP, superior lateral protocerebrum; OTU, optic tubercle; SEG, subesophageal ganglion; PRW, prow; VLP, ventrolateral protocerebrum; OL, optic lobe. **(F)** Table summary of the insertion site and the isoforms labeled by each *beat/side-MiMIC-GAL4.*

There are around 130 IgSF-encoding genes in the *Drosophila* genome [46]. In addition to well-characterized DIPs/Dprs comprised of 32 members, another two families, the Beaten path (Beat) family (14 paralogs) and Sidestep (Side) family (eight paralogs) proteins (Fig 1A), are less studied. They share many features with DIPs/Dprs. They also belong to IgSF, with two or five extracellular Ig domains mediating adhesion (Fig 1B), and form a heterophilic interaction network (Fig 1A) [30,47]. Beats/Sides have been shown to control the neuromuscular junction formation in both larval and adult motor systems [48–56]. Very recently, studies have begun to reveal their roles in synaptic specificity and induction to assemble the adult visual system [45,57–59] and sleep-regulating neuropeptidergic neurons [60]. However, little is known about whether and how they contribute to the olfactory circuit organization. We observed that the expression levels of *beats/sides* in antennal tissues increase over development, and most of them tend to have higher transcriptional levels in the latter half of the pupal stage, from 40h after puparium formation (APF) throughout adulthood (S3A Fig), during which the stereotyped glomerular map is being formed. Given their protein properties and known roles in neural development, we sought to illustrate the *beats/sides*’ expression patterns and test their functions in building the *Drosophila* olfactory circuit.

In this study, we systematically characterized the expression pattern of *beat/side* family genes in both ORNs and PNs at single-cell and single-class levels. By analyzing the previously published single-cell RNA-seq datasets and genetically probing the native expression with *MiMIC*-based *beat/side* gene trap *GAL4* lines, we revealed that each ORN or PN class expresses a unique combination of *beats/sides*, and this *beat/side* profile is likely regulated by the lineage-specific genetic programs in a hierarchical manner. We also tested the functional relevance of one interacting pair, Beat-IIa and Side-IV, based on their matching expression pattern in partner ORNs and PNs. However, perturbation of either one pre- or post-synaptically did not result in apparent glomerular mistargeting. Nonetheless, we found pervasive ORN synapse defects when the *side* was knocked down in ORNs, suggesting its possible role in synaptic development. Interestingly, we found analogous expression principles for *beat/side* orthologs across ORNs in mosquitoes and ants, indicating the shared strategies across evolution for circuit assembly through lineage-specific regulation of cell-surface protein combinations, particularly by coordinating the expression of multiple duplicated paralogs. Overall, our study reveals the combinatorial expression profile of *beats/sides* in the fly olfactory circuit and sets a foundation for further functional investigation of interacting Beat/Side proteins in neuronal circuit assembly and synaptic development.

## Results

### Genetically probing the *beat/side* expression in vivo by a collection of *MiMIC*-based gene trap driver lines

Recently, efforts to profile the transcriptional landscape of each cell across the whole fruit fly body have provided a valuable resource to examine the gene expression patterns in the cell types of interest. However, in the olfactory system, single-cell RNA-seq has only captured a limited portion of ORN or PN classes, leaving the transcriptome of many other ORN and PN classes unknown. To fully reveal the *beat/side* combinatorial profile, we used a *MiMIC*-based gene trap approach to generate a collection of transgenic *beat/side-*specific *GAL4* driver lines [62,63]. By swapping the *GAL4* construct into 5′ UTR-located *MiMIC* sites or the in-frame *T2A-GAL4* construct into introns between two coding exons, we could make GAL4 hijack the expression of the host gene (Fig 1C; Materials and methods). We then obtained *GAL4* driver lines for 13 of the 14 *beat* members and seven of the eight *side* members, except *beat-Vb* and *side-VII* (Fig 1D and 1F). Among these genes, we generated gene trap lines from two independent *MiMIC* insertion sites for five genes. These lines showed consistent labeling patterns in the brain, supporting the reliability of this method in faithfully reporting native gene expression (S1 Fig). Notably, *beat/side* genes generally have one to four annotated isoforms, and our *beat/side-MiMIC GAL4* collection is expected to trap all isoforms of each gene except *beat-IIb*, of which the *GAL4* only captures the expression of one of two isoforms (Fig 1F). This near-complete driver line collection reveals the remarkable enrichment of *beat/side* expression in neurons, from larval, pupal, to adult stages, in both peripheral and central nervous systems (Figs 1D and S2). At the gross brain level, *beats/sides* are differentially expressed across different brain regions, including the antennal lobe, central complex, mushroom body, etc. (Fig 1D and 1E). This driver collection thus provides a valuable toolkit to study *beat/side* functions in diverse neuronal contexts in the olfactory circuits and beyond. Next, we used intersectional genetic strategies to restrict the reporter expression to ORNs or PNs, which allowed us to map the *beat/side* expression across glomeruli innervated by ORNs or PNs, respectively.

### A glomerular map of *beat/side* expression in ORNs

Our antennal bulk RNA-seq through pupal development shows that *beats/sides* are generally expressed at higher levels at later stages of glomerular formation, reaching the adult levels by mid-pupal stages (S3A Fig). To delineate the *beat/side* expression in each ORN class, we examined the publicly available single-ORN RNA-seq datasets from three distinct developmental stages: 24h APF (early pupal stage), 42-48h APF (mid-pupal stage), and adulthood [13,64]. At 24h APF, ORN axons have arrived at the antennal lobes and chosen a medial versus lateral antennal lobe trajectory, which positions them en route to their future glomerular regions. By the mid-pupal stage, the glomerular targeting is almost completed, and ORNs start forming synapses with their matching postsynaptic PNs. By the beginning of the adult stage, a stereotypical and discrete glomerular map has formed, and all glomeruli are now discernible. We visualized these datasets in bubble plots showing the fraction of positive cells and the mean expression of genes of interest of each ORN class (S3B Fig). This reveals that each ORN class possesses a unique signature of *beat/side* combinations throughout the developmental stages (S3B Fig). Combining bulk antennal RNA-seq and ORN single-cell RNA-seq results, we found that *sides* are generally expressed at higher levels in ORNs than *beat*s (S3A and S3B Fig). Some *beats*, including *beat-Va*, *beat-Vb*, and *beat-Vc*, are barely expressed in any cell types within the antenna, whereas *side-VIII* appears to be predominantly expressed in non-neuronal cells of the antenna but not in ORNs (S3A and S3B Fig).

**Fig 2:**
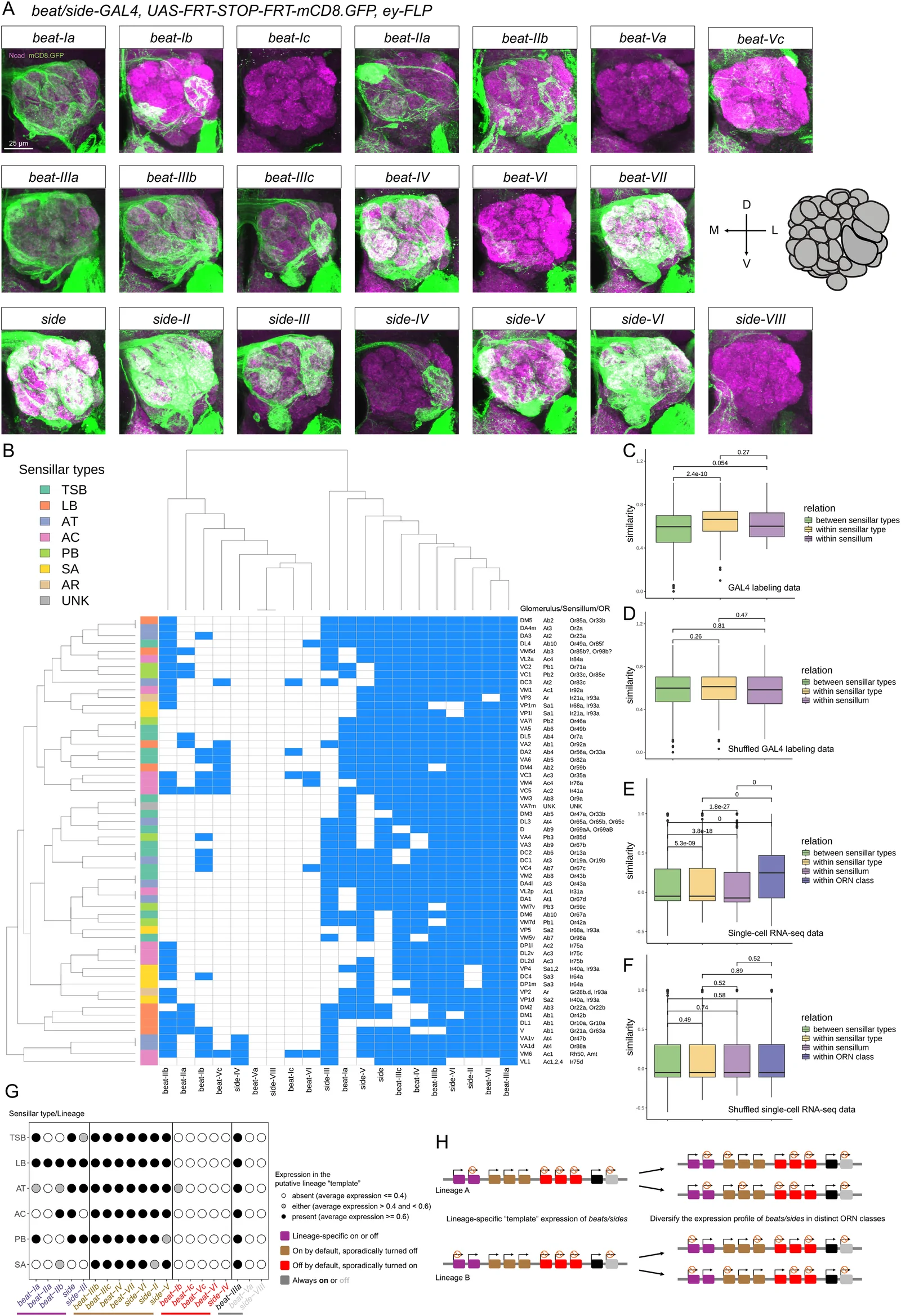
Lineage-dependent combinatorial expression of *beats/sides* across ORN classes. **(A)** A glomerular map of *beat/side* expression in ORNs revealed by transgenic *GAL4* driver lines. Glomerular structures are stained by anti-Ncad (magenta). Anti-GFP staining (green) highlights the glomeruli innervated by the indicated *beat/side*-positive ORNs. All antennal lobes shown are right antennal lobes. M, medial; L, lateral; D, dorsal; V, ventral. Each image is shown as the intensity projection over the Z-axis. **(B)** Hierarchical clustering of *beat/side* expression across ORNs based on the GAL4 labeling analysis. Row and column dendrograms are based on hierarchical clustering results. ORN class (named by its target glomerulus), the sensillum (sensillar subtype) where the ORN is housed, and the corresponding ORs expressed are shown on the right. The left row label is color-coded based on the ORN lineage. Blue means positive for the given gene expression, whereas white denotes negative. TSB, thin and small basiconics; LB, large basiconics; AT, antennal trichoids; AC, antennal coeloconics; PB, palp basiconics; SA, sacculus; AR, arista; UNK, unknown. VM6 glomerulus has been recently shown to be comprised of three subglomeruli, VM6v, VM6m, and VM6l, innervated by ORNs from Ac1 sensillum and Sacculus chamber III, respectively [6]. As it is difficult to distinguish Ac1-originated VM6v from the other two without additional colabeling, we counted VM6 as a single intact glomerulus for simplicity. **(C)** Similarity (Pearson’s correlation) of *beat/side* expression between ORN class pairs based on the expression matrix of (B) in three relation categories. **(D)** Similarity (Pearson’s correlation) of *beat/side* expression between ORN class pairs based on the expression matrix of (B), with their identity labels shuffled. **(E)** Similarity (Spearman’s correlation) of *beat/side* expression between ORN cell pairs based on the single-cell RNA-seq data in four relation categories. **(F)** Similarity (Spearman’s correlation) of *beat/side* expression between ORN cell pairs based on the single-cell RNA-seq data, with their ORN class annotation shuffled. In (C) to (F), *P* values from the Mann–Whitney *U* test of each comparison are shown. **(G)** Dot plot summarizing the putative template expression of *beats/sides* of each sensillar type. **(H)** Schematic showing the proposed model of hierarchically setting up the class-specific combinatorial expression profile of cell-surface molecules. The data underlying this figure can be found in S1, S2, and S4 Data.

As many ORN classes are not captured in the single-cell RNA-seq datasets, we used the *MiMIC-GAL4* lines to probe the ORN expression of different *beats/sides* in vivo in 3–5-day-old adult brains as the proxy of their developmental expression. We leveraged the *eyeless*-driven FLP recombinase to excise the *STOP* cassette from *UAS-FRT-STOP-FRT-mCD8.GFP*, such that membrane-localized GFP expression by *beat/side-GAL4* can be restricted to ORNs*.* This way, we can label any glomeruli innervated by ORNs expressing each *beat/side* while excluding the signal from post-synaptic PNs, where *eyeless* has no expression. As the glomerular identity in the fruit fly antennal lobe is well mapped, we manually examined individual confocal sections and assigned the expression status of each gene to every glomerulus (S4A–S4C Fig). This allowed us to generate a near-complete glomerular *beat/side* expression map across all ORN classes (Fig 2A). Consistent with bulk and single-cell RNA-seq (S3A and S3B Fig), *beat-Ic*, *beat-Va*, and *side-VIII* are not expressed in any ORN classes. Some family members, like *beat-Ib*, *beat-VI*, and *side-IV,* are only sparsely expressed in very few ORN classes (Fig 2A). On the other hand, other *beats/sides* are expressed in a much broader pattern, though at varying levels among different ORN classes (Fig 2A). We also examined the ORN expression map of several genes in the mid pupal brains (~36–48 h APF), including two genes with a restricted glomerular distribution (*beat-IIa*, *beat-IIb*), one gene with a widespread glomerular expression (*side*), and one gene that is only sparsely expressed (*beat-VI*). We found that at an earlier stage (36–48 h APF), the expression of *beat-IIa*, *beat-IIb*, and *beat-VI* in several adult glomeruli has not yet been turned on, whereas *side* is already broadly expressed in the antennal lobe (S6A and S6C Fig).

Based on the GAL4 labeling patterns, we binarized the expression of all examined *beat* and *side* genes, as positive (value = 1) or negative (value = 0) for each gene in each ORN target glomerulus. We summarized the results and hierarchically clustered the genes by their expression pattern and ORN classes by their *beat/side-*expression profile, as shown in Fig 2B. We observed three types of gene expression patterns for *beat* and *side* genes across ORN classes: (1) broadly expressed, (2) expressed at a restricted pattern, or (3) not expressed (Fig 2B). More interestingly, ORN classes from the same lineage tend to be clustered based on the combinatorial *beat/side* expression (Fig 2B). We observed similar clustering results when we semi-quantitatively binned the fluorescence signal intensities of each glomerulus into four categories: no, low, medium, and high expression for each *beat/side* gene (S5A Fig, see also in Materials and methods). This suggests a lineage-specific mechanism in patterning the ORN class-specific combinatorial expression of *beats/sides*. We decided to investigate this further.

### Lineage-regulated genetic programs specify the combinatorial expression of *beats/sides* in ORNs

ORNs differentiate from precursor cells via a multi-step hierarchical genetic program [2,9]. Early larval and pupal patterning factors first prepattern antennal imaginal discs [65], followed by the sensillar type assignment by critical transcription factors, including Lozenge (Lz), Atonal, and Amos (S3C Fig) [66–69]. We first examined the bulk antennal RNA-seq datasets profiling the transcriptional changes in mutants of *amos* (lacking trichoid and basiconic ORNs) or *atonal* (lacking coeloconic ORNs) [70,71]. We indeed found a couple of *beats/sides* that are downregulated in *atonal* and *amos* mutant antennae (S3D Fig), suggesting these *beats/sides* are enriched in the cells housed in Amos/Atonal-specified sensillar types. For example, both *beat-Ia* and *beat-IIa* are predominantly expressed in the Amos lineage-derived ORNs (Antennal Thin and Small Basiconic, TSB; Large Basiconics, LB; Antennal Tricoids, AT) and largely absent in the Atonal lineage-derived ORNs (Antennal Coeloconics, AC); these two genes are significantly downregulated in *amos* mutants (Figs 2B, S3D, and S5A). By contrast, *beat-Ib* and *side-IV* are expressed at higher levels in several AC sensillar types, despite lower-level expression in the Amos lineage, and thus show significant downregulation in *atonal* mutants (S3D and S5A Figs). A few genes are also upregulated in *amos* and *atonal* mutants (*side-VIII* in *amos* mutants, *side-II* in *atonal* mutants; S3D Fig). These *beat* and *side* genes are either transcriptionally upregulated or are now enriched in the mutants due to loss of ORNs and changes in ORN-specific transcript ratios. Together, these results suggest that the specification of *beat/side* expression is downstream of earlier ORN fate determination.

Upon sensillar type selection, sensillar subtype fates are further specified by an additional set of transcription factors like Rotund (Rn), Dachshund (Dac), and Engrailed (En) (S3C Fig) [2,72–75]. Finally, within each sensillar subtype (or sensillum), one multipotent precursor cell undergoes several sequential asymmetric divisions that eventually produce one to four different ORNs expressing distinct ORs and projecting to distinct glomeruli and four non-neuronal supporting cells [2,9]. This step is achieved by iterative recruitment of Notch signaling bifurcating cell fates, coupled with other mechanisms like epigenetic modifiers and late transcription factors [2,76,77] (S3C Fig). We here present a simplified decision tree to illustrate the kinships between ORNs and to compare *beat/side* combinations based on lineage relationships (S3C Fig): ORNs mapped to the same class, defined as “within ORN class”; ORNs of different classes but housed in the same sensillar subtype, described as “within sensillar subtype”; ORNs from different sensillar subtypes but belonging to the same sensillar type, namely, “within sensillar type”; and the furthest kinship, ORNs housed in distinct sensillar types, i.e., “between sensillar types”. We compared the pairwise similarity of *beat/side* combinatorial expression across ORN pairs belonging to these ORN kinship categories to gain insights into the developmental regulation of *beat/side* expression.

Firstly, based on our gene-ORN class binary expression matrix derived from the genetic labeling data (Fig 2B), we calculated the similarity (Pearson’s correlation) of the *beat/side* expression vector between each ORN class pair, and found significantly higher similarity between ORN classes belonging to the same sensillar types than those from different sensillar types (Fig 2C). In contrast, this pattern is not observed from the randomized control, where we shuffled the ORN class identity with the expression vector (Fig 2D). We observed the consistent results with the binned expression matrix (S5B and S5C Fig). Additionally, we found that this difference also holds for the single-cell RNA-seq datasets. By calculating the similarity (measured by Spearman’s correlation) according to gene expression between each single ORN, we found that, as expected, ORNs mapped to the same class display the highest pairwise similarity of *beat/side* profile in all stages (Figs 2E and S3E). Moreover, ORNs within the same sensillar type appear to possess more similar *beat/side* profiles than ORNs from different sensillar types, in contrast to the shuffled control (Fig 2E and 2F), supporting that the lineage-intrinsic mechanisms set the *beat/side* expression. Indeed, some genes exhibit biased expression in ORN classes of particular lineages. For example, *beat-Ia* is absent in 9 of 11 ORN classes of the antennal coeloconic lineage (AC) and 5/7 sacculus (SA) ORNs, but is expressed in the majority of all other sensillar types. On the contrary, *beat-IIa* is primarily expressed in large basiconic (LB) ORNs (6/8) but is very sparsely present in other lineages (Fig 2B). We posit that these genes are turned on as the default lineage-specific “template”. In addition to these, some genes may be poised to be expressed by default but sporadically turned off during ORN fate determination, while some genes are likely to be off by default but sparsely turned on in some terminally differentiated ORN classes (Fig 2G, see also in Materials and methods). Of note, the lineage-biased genes could also sporadically switch the expression status in a few ORNs. We therefore propose that the combinatorial expression profile of *beats/sides* is established by this two-step hierarchical mechanism: lineage-specific designation followed by sub-lineage diversification (Fig 2H).

Interestingly, we found that *beat/side* combinatorial expression tends to diverge between ORNs housed in the same sensillar subtype (S3E Fig). We observed even lower pairwise similarity according to the *beat/side* expression between these cells than between cells from different sensillar subtypes but sharing the identical sensillar type (S3E Fig). This observation stands true for all three stages (S3E Fig). In contrast, at 24h APF and mid-pupal stage, the similarity of the pan-CSM profile and the whole transcriptome linearly increases with closer kinship (S3E Fig). It suggests that the overall transcriptional profile and the cell-surface gene expression patterns are primarily set by lineage-specific factors, such that cells with closer kinship tend to have more similar transcriptomic profiles and overall cell-surface codes. Furthermore, *beats/sides* expression adds new levels of complexity to cell-surface signals, potentially diversifying ORN-specific glomerular decisions, particularly among the most developmentally related ORNs in the same sensillar subtype. Interestingly, at the adult stage, this divergence also exists for overall CSM and cell-specific transcriptional profiles (S3E Fig). These results suggest that at the end of development, ORNs with the closest kinship may have acquired broader transcriptional variance, which may support the establishment of discrete glomerular maps necessary for olfactory transduction, odor discrimination, and processing to drive odor-guided behaviors.

While the single-cell RNA-seq-based analysis only reflects the trend for the limited cell types captured in that dataset, our genetic labeling-based comprehensive analysis of *beat/side* across all ORN classes reveals the existence of both convergence and divergence: ORNs from the same sensillar subtype exhibit binary *beat/side* similarity: they could be clustered together, suggesting their shared *beat/side* combinatorial expression; they could also be segregated, indicating the divergence of *beat/side* profile from genetically very close ORNs (Fig 2B). We therefore propose a model: in some sensillar subtypes, at the step of ORN terminal selection, *beat/side* combinatorial expression is diversified from a putative initial lineage-specific “template” to ensure the closely related ORNs gain more variation in this cell-surface molecular repertoire, whereas in some sensillar subtypes, *beat/side* profile is pre-specified and these closely related ORNs show similar *beat/side* combinations.

Collectively, our thorough characterization of *beat/side* expression from both single-cell RNA-seq and gene trap *GAL4* intersectional labeling suggests that Beats/Sides could be the cell-surface “executors” underlying the hierarchical genetic programs that specify the ORN fate, likely participating in fine-tuning the ORN wiring.

### A glomerular map of *beat/side* expression in PNs

ORNs synapse with dedicated PNs within each glomerulus to assemble the olfactory circuit, and Beats/Sides are involved in synaptogenesis [57,58]. We also need to survey the *beat/side* expression on the PN side, which may inform whether Beats/Sides could function *trans-*synaptically to mediate ORN-PN matching. We started with the bulk PN RNA-seq [78] from the FACS-sorted PNs at 36 h APF and adult stage. In general, *beats/sides* appear to be expressed at commensurate levels between these two time points (S7A Fig). Next, we mined the published single-cell RNA-seq data [47,79] for developing PNs to gain insights into the PN type-specific expression of *beats/sides*. This dataset captured PNs at 0h APF, 24 h APF, 48 h APF (mid-pupal), and the adult stage. Some PNs are born embryonically and are part of the larval olfactory circuit [7,8,80,81]. At the beginning of the pupal stage, these PNs first prune their terminal axons and dendrites and re-extend their neurites to be integrated into the adult olfactory circuit following other larvally born PNs [80]. From 0 to 24 h APF, PNs project their axons to the mushroom body and lateral horn and their dendrites to the antennal lobes. PNs’ dendrites create a prototypical glomerular map before ORN axons arrive in the antennal lobes at around 24 h APF. Thereafter, PNs begin to match their presynaptic ORN partners. And starting from the mid-pupal stage, they build synapses, refine the discrete glomerular organization, and finally mature the olfactory circuit by the end of the pupal stage [7]. In terms of PN class-specific expression of *beats/side*s at different time windows, we found: (1) b*eats/sides* are enriched at 24 h APF and thereafter, while expressed at minor levels at the beginning of metamorphosis (S7B Fig); (2) compared with ORNs, *beats/sides* are generally expressed in a broader pattern, and all *beats/sides* are expressed in PNs while some *beats* and *side-VIII* are undetectable in ORNs (S3B and S7B Figs). Suppose Beats/Side mediate adhesion between ORNs and PNs; this observation raises a hypothetical model where broadly and less specifically located postsynaptic surface “locks” are matched by sparsely and more specifically distributed presynaptic surface “keys”.

**Fig 3:**
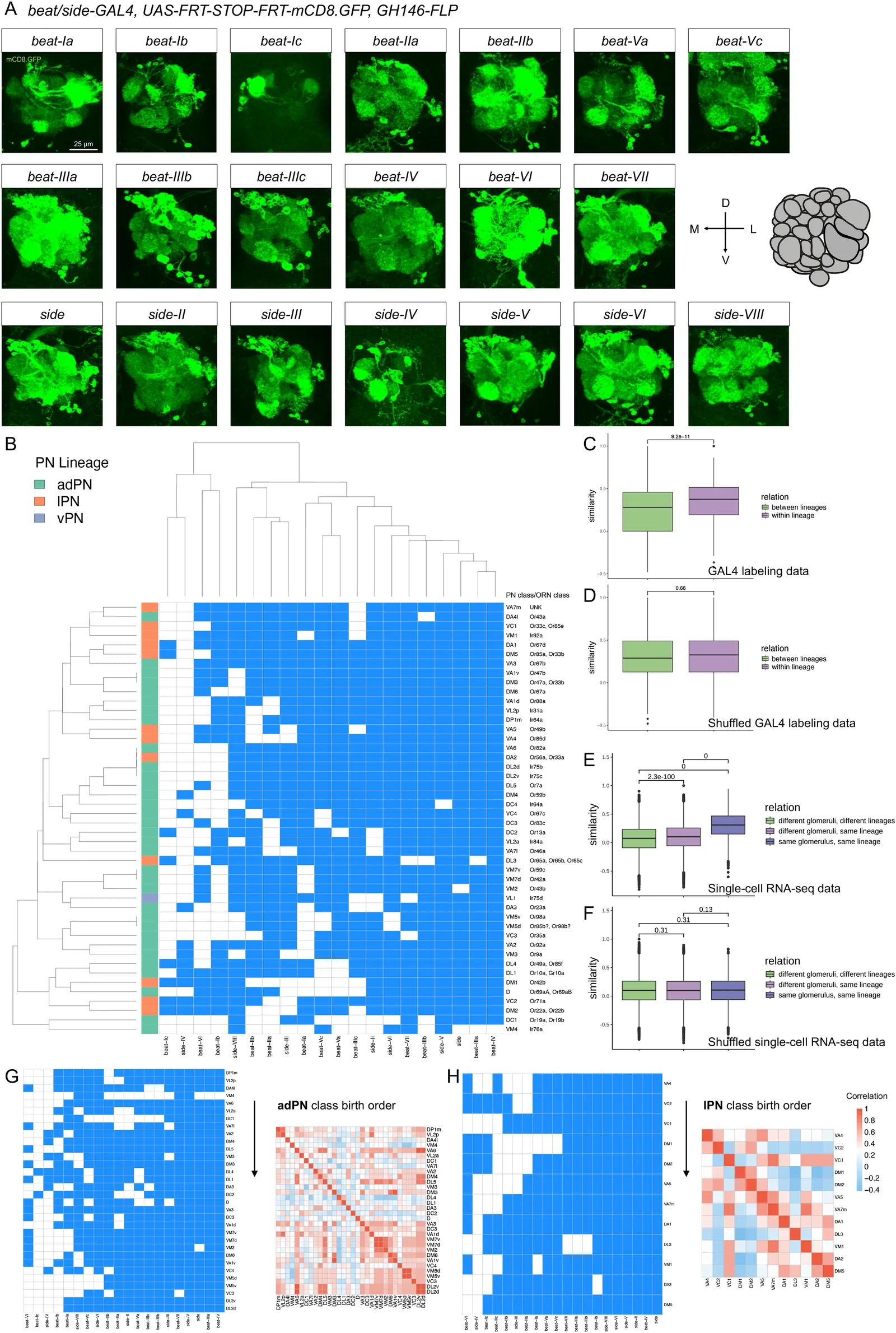
Lineage-correlated combinatorial expression of *beats/sides* across PN classes. **(A)** A glomerular map of *beat/side* expression in PNs revealed by transgenic *GAL4* driver lines. Only the GFP signal (green) highlights the glomeruli innervated by the indicated *beat/side*-positive PNs. Neuropils were stained with anti-Ncad antibody to help determine the glomerular identity but are not shown for visualization contrast. Example single confocal sections showing both the GFP signal and Ncad staining can be found in S4 Fig. All antennal lobes shown are right antennal lobes. M, medial; L, lateral; D, dorsal; V, ventral. Each image is shown as the intensity projection over the Z-axis. **(B)** Hierarchical clustering of *beat/side* expression across PNs based on the GAL4 labeling analysis. Row and column dendrograms are based on hierarchical clustering results. PN class and its matching ORN class (named by corresponding ORs expressed) are shown on the right. The left row label is color-coded based on the uniglomerular PN lineage. Blue indicates positive for the given gene expression, whereas white denotes negative. Only the glomeruli with decoded PN lineage are shown in this figure [8,81]. The DC4 glomerulus was assigned to the adPN lineage based on the FlyWire dataset [82,83]. Several glomeruli, including V, DP1l, and DA4m, among others, are not labeled by the GH146-FLP and are also excluded. Of note, we frequently observed labeling of vPN-like neurites in the VA1v and DA1 glomeruli, with *beat-V*I showing particularly strong vPN-associated signals (see S4 Data for the full annotated expression matrix with lineage details). **(C)** Similarity (Pearson’s correlation) of *beat/side* expression between PN class pairs based on the expression matrix of (B) in two relation categories. **(D)** Similarity (Pearson’s correlation) of *beat/side* expression between PN class pairs based on the expression matrix of (B), with their identity labels shuffled. **(E)** Similarity (Spearman’s correlation) of *beat/side* expression between PN cell pairs based on the single-cell RNA-seq data in three relation categories. **(F)** Similarity (Spearman’s correlation) of *beat/side* expression between PN cell pairs based on the single-cell RNA-seq data, with their ORN class annotation shuffled. In (C) to (F), *P* values from the Mann–Whitney *U* test of each comparison are shown. **(G)** Combinatorial expression matrix (left) of adPN classes and the Pearson’s correlation matrix based on the *beat/side* expression between each PN class pair (right). The expression matrix is replotted from (B), and PN classes are ordered based on the known birth sequence, while *beat/side* genes are ordered based on the hierarchical clustering result. Rows and columns in the correlation matrix are also ordered in the same way. **(H)** Combinatorial expression matrix (left) of lPN classes and the Pearson’s correlation matrix based on the *beat/side* expression between each PN class pair (right). The expression matrix is replotted from (B), and PN classes are ordered based on the known birth sequence, while *beat/side* genes are ordered based on the hierarchical clustering result. Rows and columns in the correlation matrix are also ordered in the same way. The legend depicting the correlation levels also applies to the correlation matrix in (G). The data underlying this figure can be found in S1, S3, and S4 Data.

Next, we used these *GAL4* lines to drive the GFP reporter expression in postsynaptic PNs by removing the *STOP* cassette from *UAS-FRT-STOP-FRT-mCD8.GFP* with the PN-specific *GH146-flippase*. We found generally broader expression of *beats/sides* across PN-labeled glomeruli, with a few sparsely distributed ones, such as *beat-Ic* and *side-IV* (Fig 3A). We validated the PN expression of *side-IV* in the developing brain (36–48 h APF), and found that it is expressed in fewer glomeruli than in the adult brain. Among these, glomeruli positive for *side-IV* in pupal stages appear to maintain expression throughout development, while others seem to be turned on in later stages (S6B and S6C Fig). Using the fully mapped antennal lobe template, we examined individual confocal imaging planes to assign the expression status of each gene to every glomerulus (S4A, S4D, and S4E Fig). We then summarized the binary expression of all *beats/sides* examined across all glomeruli identified (Fig 3B). Similarly to ORNs, we also discretized the expression into four ordinal levels, no, low, medium, and high expression (S8A Fig). Hierarchical clustering of PN types according to their *beat/side* expression shows, in general, a correlation between *beat/side* combination and their PN lineages (Figs 3B and S8A). We therefore further investigated this.

### Lineage-regulated genetic programs specify the combinatorial expression of *beats/sides* in PNs

PN fates are specified separately from ORNs by a sequential genetic program from three distinct neuroblast lineages (S7C Fig) [7,9]. A common neuroblast sequentially produces one ganglion mother cell and a self-renewed neuroblast. The ganglion mother cell further divides into one terminal PN, which innervates a dedicated glomerulus, and another daughter cell, which undergoes cell death [81,84]. The younger neuroblast continues this process. Thus, different PN classes are born in a sequentially stereotyped order [7,8,81]. Based on this, we defined the relationship between PNs into three categories: (1) PNs from the same lineage targeting the same glomerulus, (2) PNs from the same lineage targeting different glomeruli, and (3) PNs from different lineages targeting different glomeruli.

We again asked whether the *beat/side* profile similarity between PN classes is based on their developmental kinship. Calculating the PN class-specific *beat/side* expression similarity based on the *GAL4* labeling expression matrix showed higher similarity between PN classes of the same lineage than of different lineages (Figs 3C, 3D, S8B and S8C). Similarity analysis for *beat/side* expression in the single-PN RNA-seq datasets also confirmed these observations (Figs 3E, 3F, and S7D). In the same lineage, as expected, PNs targeting the same glomeruli also have more similar *beat/side* expression than PNs targeting different glomeruli (S7D Fig). This is also true for the expression of CSMs and the global transcriptome at all four stages (S7D Fig). This suggests that, as part of their cell-surface repertoire, the *beat/side* profiles in PNs are primarily set by the lineage and PN class-specific genetic mechanisms.

As PNs in each lineage are born in a stereotyped order, we also examined whether PN birth order determines its *beat/side* profile. We calculated the correlation between each PN pair. We plotted their *beat/side* profile and sorted the PN classes in each lineage based on the reported birth order (Fig 3G and 3H). We also plotted the Pearson’s correlation coefficient between each pair of PN classes in the respective lineage (Fig 3G and 3H). We indeed found that many adjacently born PNs share a more similar *beat/side* profile in both lineages, though examples of divergence also exist (Fig 3G and 3H). In the adPN lineage, early-born PNs have more divergent *beat/side* profiles while later-born PNs gradually obtain a shared Beat/Side combinatorial “template” which gets less complex as new PN fates are generated (Fig 3G). In the lPN lineage, temporally adjacent PNs appear to acquire a more similar *beat/side* profile than the ones born distantly (Fig 3H). We also replicated this analysis in the binned expression matrix derived from the GAL4 labeling imaging dataset, and observed similar results (S8D and S8E Fig). This trend indicates that PN precursor-specific factors set the *beat/side* expression potential of daughter PNs and undergo a temporal fate specification along with the birth sequence of PNs. It is also interesting that, similar to ORNs (Fig 2B and 2C), closely related PNs can possess shared or divergent *beat/side* combinatorial expression, suggesting that different intrinsic genetic programs may be present in different precursor cells to control the opposing *beat/side* expression similarity in their daughter cells.

### Expression of Beat/Side proteins in the antennal lobe

Recent proximity labeling-based cell-surface proteomic profiling found several Beasts/Sides are among the top proteins enriched on the ORN (Side-VI, Side-V, Side) and PN (Beat-IV, Side, Side-III, Side-VII, Beat-IIIC, Beat-VI) surface [78,85]. To examine the in vivo glomerular expression of Beat/Side proteins, we used *MiMIC*-based protein-trap animals, where, instead of the *GAL4* cassette, a GFP protein tag (the full tag contains EGFP-FlAsH-StrepII-TEV-3xFlag, and here we term GFP for simplicity) is inserted into the *MiMIC* site as an artificial exon [86]. We obtained a collection of available transgenic flies expressing *MiMIC-GFP*-tagged Beats/Sides. In particular, tagged Side protein exhibits very similar brain-wide localization patterns to that of the *side-T2A-GAL4*-driven membrane-bound myr.GFP reporter (S9D Fig), suggesting the reliability of these genetic reagents. Overall, we found signals for Beat/Side proteins in both presynaptic ORNs and postsynaptic PNs or local interneurons within the antennal lobe (S9 Fig). For example, *side-VI* is broadly expressed across ORNs and PNs (Figs 2B and 3B) but is excluded in DM1-innervating ORNs. Concordantly, Side-VI-GFP signal is present in the ORN axonal commissure and shows lower yet still detectable expression in the DM1 glomerulus (S9F Fig). This suggests additional Side-VI protein localization to non-ORN neurites innervating the DM1 region, likely from PN dendrites or local interneurons. Another glomerulus, DC4, which is targeted by *side-VI-*positive ORNs, shows a marked Side-VI protein signal (S9F Fig). Beat-Va is an interacting partner of Side-VI and is not expressed in any ORNs based on the GAL4 labeling or RNA-seq results (Figs 2B and S3B) but is expressed in many PN classes (Fig 3B). We also detected a Beat-Va protein signal across glomeruli, likely reflecting the neurite localization from PN or local interneurons (S9C Fig). Additionally, the *side-V* gene has a characteristic high expression in Gr21a ORNs, and the elevated signal of the tagged Side-V protein could also be detected in the corresponding V glomerulus (S9E Fig). Unlike *beat-Va*, many *beat* genes are expressed in both ORNs and PNs (Figs 2B and 3B). For example, we observed signals of Beat-IIb- and Beat-IV- tagged proteins in ventral PN (vPN) axons projecting to higher brain regions (S9A and S9B Fig). In parallel, the Beat-IV-GFP signal is also detected in ORN axons and pervades the antennal lobe, and is lower yet detectable in the DM1 glomerulus, which is negative for *beat-IV*-expressing ORNs (S9B Fig). It is noteworthy that these tagged proteins may not always reflect the native subcellular localization, depending on where the tag is inserted and whether the insertion interferes with the proper folding, processing, and trafficking of the native protein. We found several *MiMIC-*tagged Beat/Side proteins, including Beat-Ib, Beat-VI, Beat-VII, and Side-III, with GFP-tag signal predominantly localized to cell soma and barely detected in neurites (S9G Fig), likely owing to disrupted protein function by the inserted tag. In summary, we validated the in vivo subcellular localization of some Beat/Side proteins and confirmed their expression across glomeruli in the antennal lobe, supporting their functions as cell adhesion molecules to mediate the interactions between neurites in the antennal lobe.

### Beats/Sides form a robust molecular interaction network between ORN and PN partners

**Fig 4:**
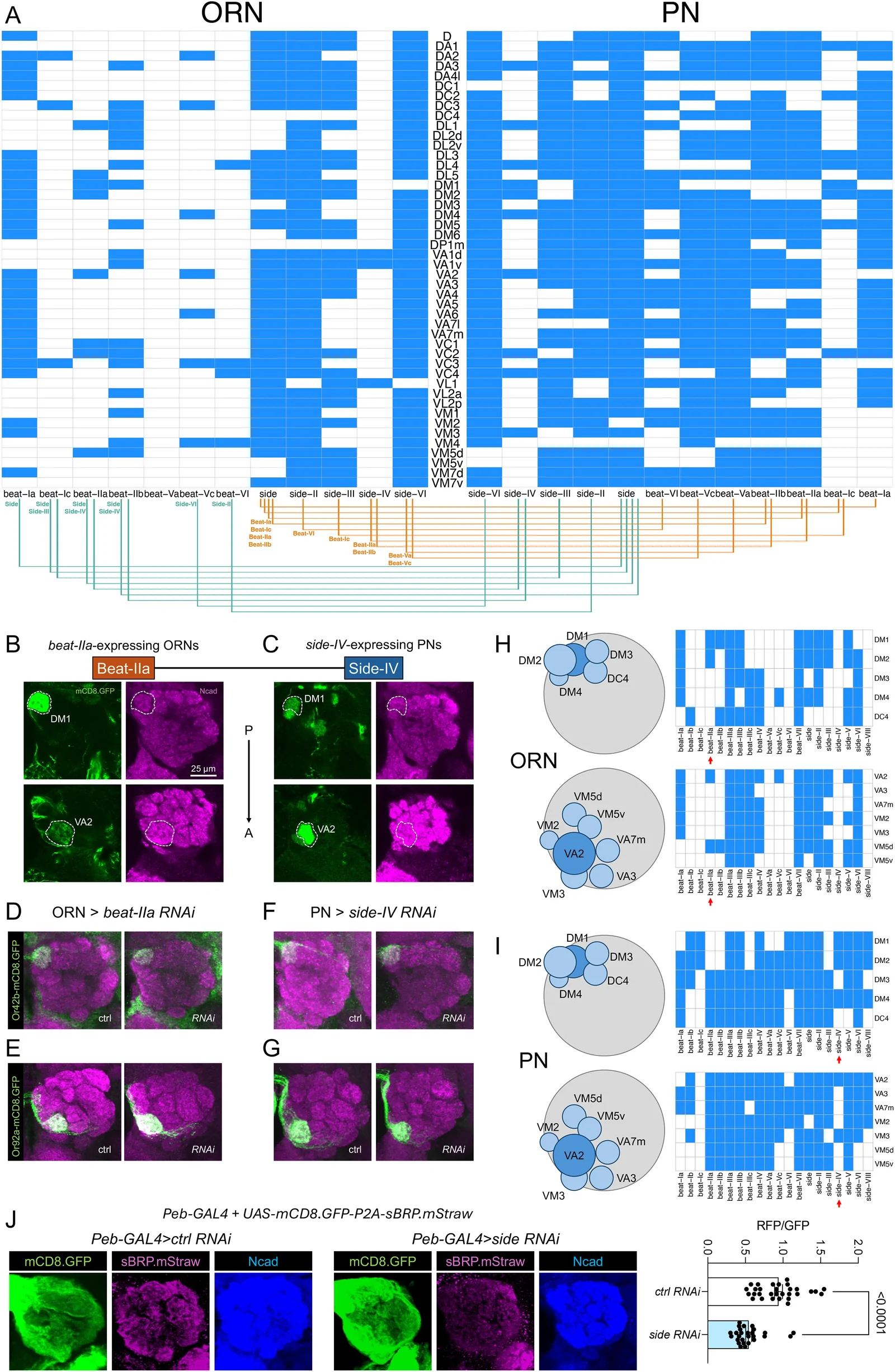
Beats-Sides interactions between matching ORN-PN partners can form a redundant, error-tolerant surface “barcode”, enabling recognition robustness and mediating synaptogenesis. **(A)** Potential *trans-*synaptic Beat/Side interactions between matching ORN-PN classes targeting each glomerulus. The ORN (left) and PN (right) profile of beat/side expression is based on Figs 2B and 3B, but it is replotted to highlight the ORN class and PN class innervating the same glomerulus. Based on in vitro biochemical characterization (Fig 1A), known binding interactions between Beats and Sides are indicated as lines. Green lines represent Beats -> Sides interactions, and red lines represent Sides -> Beats interactions (ORN -> PN). **(B)** ORNs targeting DM1 and VA2 glomeruli expressing *beat-IIa*, labeled by the intersection of *beat-IIa-T2A-GAL4* and *ey-FLP* to drive *UAS>STOP>mCD8.GFP*. Scale bar representing 25 µm also applies to each antennal lobe panel in (C) to (G) and (J). **(C)** PNs targeting DM1 and VA2 glomeruli expressing *side-IV*, labeled by the intersection of *side-IV-GAL4* and *GH146-FLP* to drive *UAS>STOP>mCD8.GFP*. In (B) and (C), the left columns show anti-GFP staining (green) and the right columns show anti-Ncad staining (magenta). **(D, E)** Knockdown of *beat-IIa* in ORNs by a pan-ORN driver, *peb-GAL4,* while labeling Or42b ORNs targeting DM1 glomerulus (D) and Or92a ORNs targeting VA2 glomerulus (E). **(F, G)** Knockdown of *side-IV* in PNs by a PN driver *GH146-GAL4* while labeling Or42b ORNs targeting DM1 glomerulus (F) and Or92a ORNs targeting VA2 glomerulus (G). Neuropils were stained with anti-Ncad antibody, shown in magenta. Representative images from 7 to 12 brains examined in each condition are shown (D–G). **(H)** The Beat/Side “barcode” in ORNs targeting DM1 or VA2 and their neighboring glomeruli, based on the *beat/side-GAL4* labeling results in Fig 2B. **(I)** The Beat/Side “barcode” in PNs targeting DM1 or VA2 and their neighboring glomeruli based on the *beat/side-GAL4* labeling results in Fig 3B. In (H) and (I), the schematics show the antennal lobe (gray circle) and the relative positions of the indicated glomeruli (blue circles). Red arrows indicate the perturbed genes, *beat-IIa* and *side-IV*, respectively. **(J)** Representative antennal lobes and quantification showing the effects of knockdown of *side* on gross glomerular synaptic density, visualized by the membrane marker GFP and presynaptic marker RFP. Each dot is a single antennal lobe. *N* = 30 and 27 antennal lobes, respectively. *P* value from the unpaired *t* test between groups is shown. The data underlying this figure can be found in S1 and S4 Data.

The first identified Beat-Ia and Side pair act *trans-*synaptically to promote synapse formation at the neuromuscular junction [48,50,52–54]. Recent work also identified additional Beat-Side interactions, including Side-IV and Beat-IIa/b, mediating neuronal recognition, connection specificity, and synaptogenesis in the fly visual system [57,58]. We therefore hypothesized that complementary expression of interacting Beat and Side proteins might regulate synaptic matching between ORNs and PNs. To test this hypothesis, we examined the *beat/side* expression between partner ORN and PN targeting the same glomerulus based on our expression maps as well as the known Beat-Side interactions (Figs 1A and 4A). This analysis revealed a putative Beat/Side-mediated synaptic interaction between ORNs and PNs, which is promiscuous, redundant, and complex.

To test whether *beat/side* plays any role in glomerular targeting or organization, we conducted a pan-ORN *peb-GAL4-*driven *UAS-RNAi* screening against *beat* and *side* genes exhibiting decent expression in specific ORNs and examined whether three glomeruli would show any defects as a readout. These include Gr21a ORNs, Or47b ORNs, and Or47a ORNs, targeting V, VA1v, and DM3 glomerulus, respectively (S10A Fig). We included perturbation of *side-VIII*, which is not expressed in any ORNs, as an additional negative control. For each gene perturbed, we used two independent approaches: either two different *RNAi* lines to knock down or one *RNAi* line and one gRNA line inducing a somatic knockout (S10B Fig). First, we noticed that V glomerulus appeared wild-type in all perturbations (S10A Fig). Second, this screen revealed local organizational defects in the VA1v glomerulus by knockdown of *beat-IIb, beat-IIIa/b/c,* and *side-III*. Among these, the VA1v phenotype was reproduced by two independent *RNAi* lines against *beat-IIb*, but the defects in *beat-IIIa/b/c* or *side-III* knockdowns could not be robustly observed in secondary perturbations (S10B Fig). Additional controls showed that the VA1v phenotypes of *beat-IIIa/b/c* knockdowns appeared to arise from the transgenic background that sensitizes the VA1v glomerular disorganization [87] (S4C Fig). Third, we observed a low-penetrance phenotype of split DM3 glomerulus, which appeared in both *RNAi* knockdown and gRNA-mediated ORN-specific knockout of *beat-II*Ic (S10A, S10B, and S10E Fig). Overall, we identified two hits with mild glomerular phenotypes from this targeted screen: *beat-IIb* in Or47b ORNs and *beat-IIIc* in Or47a ORNs. However, Beat-IIb-interacting Side-IV is not expressed in postsynaptic PNs innervating the VA1v glomerulus or nearby regions. Coincidentally, *side-IV* is restrictedly expressed in Or47b and Or88a ORNs, which target a neighboring glomerulus (Fig 2A and 2B), raising the possibility for axon–axon contacts mediated by co-expressed Beat-IIb and Side-IV, which is interesting for future exploration. Additionally, Beat-IIIc is an orphan cell-surface protein with no characterized interacting partners (Fig 1A), which limits our further investigation of its role in glomerular organization. Moreover, we made a preliminary effort with multiplexing perturbation. We found that *beat-IIa* and *beat-IIb* are markedly co-expressed in the V glomerulus-innervating ORNs. Side-IV, which interacts with Beat-IIa and Beat-IIb, appears to be expressed in PNs targeting the V glomerulus as well (S10F Fig). We therefore used ORN-specific CRISPR to simultaneously knock out *beat-IIa* and *beat-IIb* but didn’t find an overtly defective glomerular organization (S10F Fig). In addition, we also performed gain-of-function experiments using pan-ORN overexpression of *beat* genes that are sparsely expressed across ORNs, but did not observe any obvious mistargeting phenotypes (S10G Fig).

We then turned to other glomeruli with the matching expression of interacting Beats and Sides between partner ORN/PN classes. We found one straightforward case: presynaptic Beat-IIa and postsynaptic Side-IV. *beat-IIa* is expressed in just a few ORN classes, including Or42b ORNs targeting one posterior glomerulus DM1 and Or92a ORNs targeting one anterior glomerulus VA2 (Fig 4B). In parallel, *side-IV* expression is restricted in PNs, including PNs projecting to DM1 and VA2 glomeruli (Fig 4C). Next, we tested whether perturbing *beat-IIa* expression in ORNs or *side-IV* expression in PNs results in glomerular targeting defects, using the *RNAi* lines validated with reported phenotypes in previous studies [58,88]. We used *peb-GAL4* to knock down *beat-IIa* in all ORNs while labeling Or42b ORNs or Or92a ORNs as a readout of glomerular targeting. However, we did not observe any gross phenotype in glomerular integrity, morphology, position, or ectopic mistargeting (Fig 4D and 4E). We also used a PN-specific driver, *GH146-GAL4,* to express *side-IV RNAi* and test if Or42b or Or92a ORNs target their glomeruli normally. Again, we found no visible differences between knockdowns and controls (Fig 4F and 4G). These results suggest that either Beat-IIa/Side-IV interaction in matching ORNs and PNs is unnecessary for glomerular targeting or is compensated by other Beat and Side proteins with redundant functions expressed in the same neurons.

Indeed, the combinatorial *beat/side* codes can distinguish ORNs targeting DM1 or VA2 glomeruli from ORNs innervating the neighboring glomeruli (Fig 4H), such that removing *beat-IIa* in ORNs doesn’t ambiguate the CSM repertoire and still preserves a level of diversity among the local axon fibers in a glomerular neighborhood. Similarly, PNs targeting DM1 or VA2 also harness this robust combinatorial “barcode”, and deleting *side-IV* in PNs may not abolish the dendritic diversity in the glomerular neighborhood (Fig 4I). These negative results might be due to the Beat/Side combinatorial expression, which can theoretically act as a robust cell-surface recognition code for each ORN or PN class, given its error-tolerance feature. Nonetheless, the comprehensive Beat-Side interacting atlas we documented here can inform complex multiplexing manipulation experiments to further interrogate the functions of Beats/Sides in ORN circuit assembly.

Given the lack of glomerular targeting defects, we next asked whether *beat* and *side* genes have roles in regulating synapse development and maintenance within glomeruli. For this, we used a previously generated transgenic reporter, *UAS-SynLight* (*UAS-mCD8.GFP-P2A-sBRP.mStraw*), which expresses the presynaptic active zone marker, Bruchpilot-Short (sBRP), and the neurite membrane marker mCD8.GFP in the same transcript [89]. We expressed this transgene in ORNs to label and quantify the synaptic density in the antennal lobes while doing perturbation in ORNs. We calculated the ratio of the mean fluorescence intensity of RFP and GFP in the antennal lobe as a proxy of synaptic density (S11A Fig). This way normalizes the potential variability in transcription levels, imaging conditions, or neuron size across groups. As inhibiting neural activity has been reported to decrease synapse numbers, we first silenced ORN neuronal activity by overexpressing the mutant *shaker* channel EKO. We observed synaptic density reduction globally in the antennal lobe, compared with the control group where an inactive variant of tetanus toxin light-chain (TNT-) was introduced instead (S11B and S11C Fig). This confirmed the feasibility of using this transgenic reporter to measure synapses and the effect of neural activity on synaptogenesis.

We next used the pan-ORN *peb-GAL4* to knock down *beats/sides* that are expressed in ORNs and have known interacting partners. We also knocked down *side-VIII*, which is not expressed in ORNs, to serve as a presumable negative control (Figs 2A, 2B, and S3B). We found a pervasive synaptic reduction in *side* knockdowns compared to the control *RNAi,* but not *side-VIII* knockdowns (Figs 4J, S11D, and S11E). In contrast, knockdown of sparsely expressed genes, including *beat-IIa/b* and *side-IV*, didn’t lead to detectable differences from controls (Figs 4A, S11D, and S11E). Additionally, knockdown of other broadly expressed ones, including *beat-Ia*, *side-II, side-III*, and *side-VI*, also didn’t cause significant changes in synaptic density of ORNs, except *side-VII*, which increased the overall synaptic density in the antennal lobe (Figs 4A, S11D, and S11E). The discrepancy of loss-of-function effects between *side* and other broadly expressed ones could be due to the biochemical property of Side protein, which interacts with four Beat partners, more than any other Side family members (Fig 1A). It is likely that a lack of Side leads to a more substantial reduction in *trans-*synaptic adhesion than the lack of other Side proteins (Fig 4A). Unlike potential functions in synapse induction, *side-VII* might play a role in inhibiting synapse formation. Though we were unable to map the *side-VII* expression pattern in the antennal lobe as the gene trap driver is unavailable, the single-cell RNA-seq datasets show that *side-VII* appears to be broadly expressed across ORN classes and may interact with its cognate partner Beat-IV, which is also broadly expressed across PN classes as well as ORN classes (Figs 2B, 3B, S3B, and S7B). Thus, Side-VII might inhibit synapse formation through either *trans* interaction with post-synaptic Beat-IV or *cis* binding with Beat-IV on the same membrane. Notably, elevated or ectopic synapse formation was observed in the fly visual circuit in *side-VII* mutants [58]. Overall, our results suggest the bidirectional regulatory functions of Beat/Side proteins in ORN synapse formation.

Interestingly, analysis of previously reported antennal RNA-seq data [90] from *Or47b* and *Or67d* mutants revealed differentially expressed *beat* and *side* genes (S11F Fig). We find that certain *beat* and *side* genes are up-regulated while others are down-regulated in olfactory receptor mutants (S11F Fig). These results suggest that these genes are responding to neural activity and might contribute to ORN-specific synapse development and maintenance. Notably, the transcriptional changes induced by OR-dependent neural activity, in addition to lineage factors, can contribute to the final combinatorial expression and synaptic function of Beat and Side proteins.

### Evolutionarily conserved expression of *beat/side* orthologs across insect ORNs

**Fig 5:**
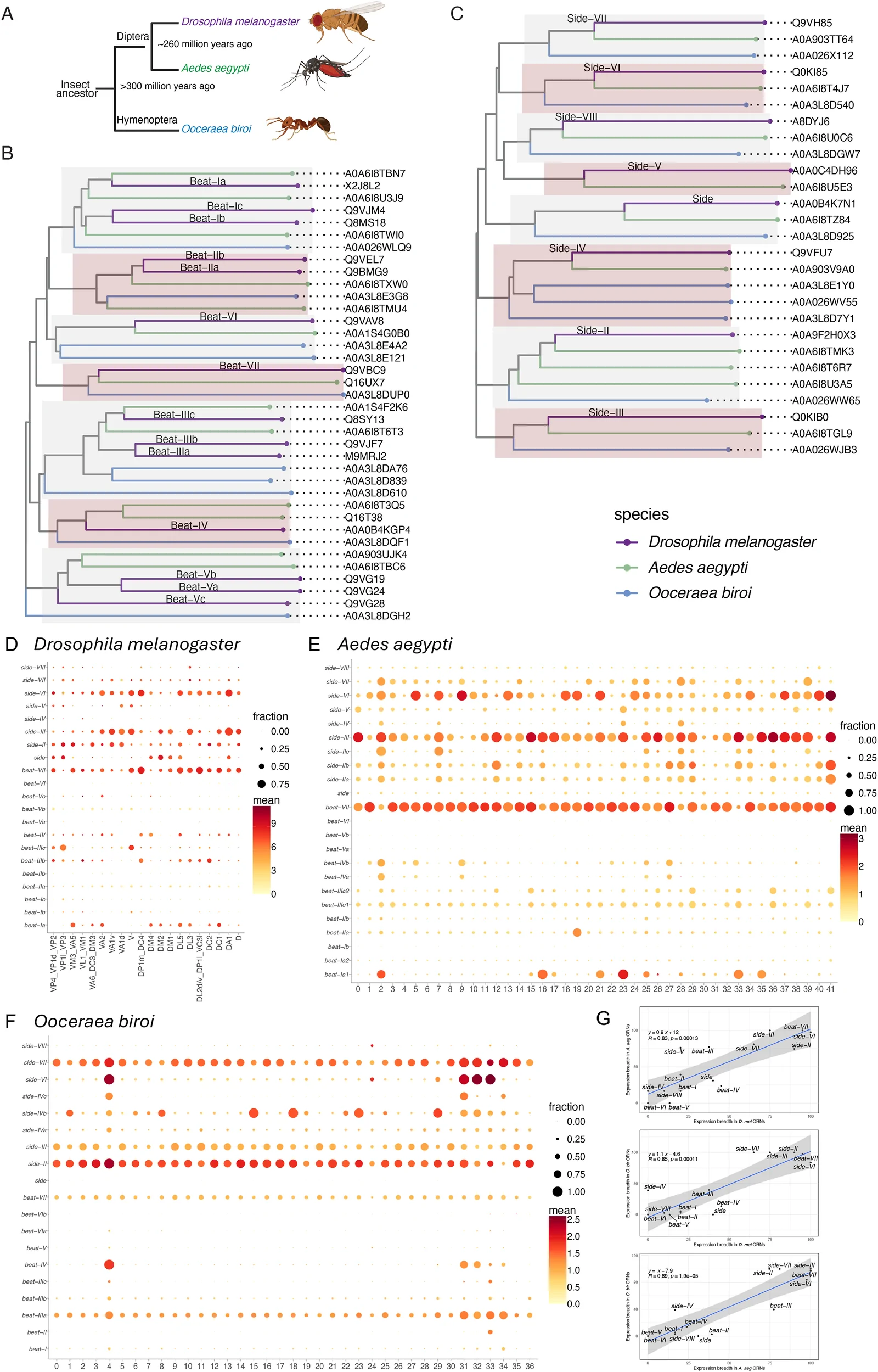
Evolutionarily conserved expression pattern of *beats/sides* across ORNs in insects. **(A)** Schematic phylogeny showing the evolutionary distance among three insect species. Created in BioRender. Duan, Q. (2026) https://BioRender.com/bqpfhvi. **(B)** Phylogeny tree of Beat orthologs in fruit flies *Drosophila melanogaster*, yellow fever mosquitoes *Aedes aegypti*, and clonal raider ants *Ooceraea biroi*. **(C)** Phylogenetic tree of Side orthologs across three insect species. In (B) and (C), each leaf is color-coded by the species, shown in the bottom right legend. Each ortholog is displayed as the UniProt protein ID, while the known *Drosophila* Beat/Side names are labeled on the corresponding branch. **(D)** Expression of *beats/sides* in the adult ORN classes of fruit flies based on the single-cell RNA-seq data from [64]. Each row is a gene, and each column represents an annotated ORN class. The size of each dot represents the percentage of positive cells in the given class (log2(CPM + 1) > 0.5), and the color denotes the mean expression levels. This panel was reproduced from S3B Fig for comparison. **(E)** Expression of *beats/sides* in the adult ORN clusters of yellow mosquitoes based on the single-cell RNA-seq data from [91]. The mosquito *beats/sides* were renamed based on their phylogenetic relations with the fruit fly orthologs. **(F)** Expression of *beats/sides* in the adult ORN clusters of clonal raider ants based on the single-cell RNA-seq data from [92]. The ant *beats/sides* were renamed based on their phylogenetic relations with the fruit fly orthologs. **(G)** Linear regression analyses showing the high correlation of expression breadth (defined by the proportion of ORN clusters in which a gene is expressed based on the single-cell RNA-seq data) of *beat/side* orthologs between insect species. The expression breadth of paralogs in each clade (i.e., *beat-Ia/b/c*, *beat-IIa/b*, etc) is averaged. The data underlying this figure can be found in S1 and S5 Data.

Next, we sought to interrogate the ORN class-specific expression of *beats/sides* in other species to gain some evolutionary insights into the regulation and function of these families of IgSF proteins. Recently reported transcriptome atlases of ORNs in two additional insect species, yellow fever mosquitoes *Aedes aegypti*, and clonal raider ants *Ooceraea biroi*, allowed us to perform comparative analyses of *beat/side* expression in the peripheral olfactory tissue at single-cell resolution. Yellow fever mosquito and clonal raider ants belong to the order Diptera and a distant order Hymenoptera, and diverged from the fruit fly *Drosophila melanogaster*, a Dipteran species, 260 million and 300 million years ago, respectively (Fig 5A). We first set out to identify the bona fide orthologs of *beats/sides* in mosquitoes and ants. To do this, we queried the protein sequence of *Drosophila* Beat-Ia and Side against the proteome database of two other species and ran multiple sequence alignment after selecting the hits with comparable lengths and similar AlphaFold-predicted structures (two Ig domains for Beats; five Ig domains and one Fibronectin domain for Sides, also see in Materials and methods, S12A Fig). We then built the phylogenetic tree of Beat/Side protein orthologs across the three species. This analysis yielded 13 *beat* genes and 10 *side* genes in the yellow fever mosquito, while the clonal raider ant has 10 *beats* and nine *sides* in its genome (Fig 5B and 5C). We observed several notable gene duplication/loss events in these species. For example, there is a single ant gene in the Beat-I clade, whereas there are three copies in mosquitoes and flies (Fig 5B). In contrast, three ant genes encode the Side-IV clade and three mosquito genes encode the Side-II clade, while only one fruit fly gene is in each clade (Fig 5C). Additionally, the *side-V* gene appears to be lost in clonal raider ants (Fig 5C). The phylogenetic tree indicates that: (1) gene duplication events generated ancestral *beat/side* paralogs earlier than insect species divergence; (2) additional gene duplication/loss events also occurred post-divergence of fruit fly, mosquito, and ant species.

Based on the phylogenetic tree, we renamed the mosquito and ant genes according to their sequence similarities with the fruit fly orthologs (Table 3). Recent studies provided the single-cell RNA-seq atlases for ORNs in several mosquito and ant species [91–94]. We therefore examined the expression of mosquito and ant *beats/sides* in ORNs from two of these published single-cell datasets [91,92]. Strikingly, we found that combinatorial expression principles of *beat/side* genes in different ORN classes across the three insect species are conserved (Figs 5D – 5F and S12B): (1) genes that are not expressed in *Drosophila* ORNs, like *beat-Va/b/c*, *beat-VI*, and *side-VIII*, are also not expressed in ORNs of yellow fever mosquitoes (*beat-Va/b*, *beat-VI*, *side-VIII*) or clonal raider ants (*beat-V*, *beat-VIa/b*, *side-VIII*); (2) genes that are broadly expressed in *Drosophila* ORNs, like *beat-VII*, *side-II*, and *side-III*, are also broadly expressed in ORNs of yellow fever mosquitoes (*beat-VII*, *side-IIa/b*, and *side-III*) and clonal raider ants (*beat-VII*, *side-II*, and *side-III*); (3) genes that are expressed in a restricted pattern across *Drosophila* ORNs, like *beat-IIa/b*, beat*-IV*, and *side-IV* (Fig 2A and 2B), are also expressed in only a portion of ORN clusters in mosquitoes (*beat-IIa*, beat*-IVa/b*, and *side-IV*) and ants (*beat-II*, beat*-IV*, and *side-IVb/c*). Linear regression of the expression breadth of these orthologs in ORNs supports the overall high correlation between species (Figs 5G and S12D). Even though there is significant conservation of *beat/side* gene expression patterns across ORNs, there are also a few exceptions. For example, *beat-Ia*, expressed in many ORN classes in fruit flies and at high levels in restricted ORN classes in mosquitoes, is barely expressed in ant ORNs. In addition, *side* is expressed in many *Drosophila* ORN classes but is undetectable in mosquito and ant ORNs (Fig 5D – 5F). These comparative analyses suggest that the conserved and species-specific expression patterns of *beat* and *side* genes in peripheral sensory neurons likely arose through evolutionary events in the genome, including the duplication and loss of *beat/side* family members, as well as the conservation and divergence of their regulatory sequences. Importantly, the striking conservation of *beat* and *side* expression patterns in ORNs across species suggests that these expression programs are under selective pressure, supporting their functional relevance in ORN circuit development and/or operation.

**Table 3:** Orthologs of *beats/sides* in yellow fever mosquitoes *Aedes aegypti* and clonal raider ants *Ooceraea biroi.*

UniProt accession	Gene ID	Length (aa)	*D. mel* orthologs	New gene name
***A. aegypti***				
A0A6I8TBN7	LOC5566816	403	Beat-Ia/Ib/Ic	*beat-Ia1*
A0A6I8U3J9	LOC5572937	329	Beat-Ia/Ib/Ic	*beat-Ia2*
A0A6I8TWI0	LOC110676758	293	Beat-Ia/Ib/Ic	*beat-Ib*
A0A6I8TXW0	LOC5566409	329	Beat-IIa/IIb	*beat-IIa*
A0A6I8TMU4	LOC5564746	296	Beat-IIa/IIb	*beat-IIb*
A0A1S4F2K6	LOC5575664	317	Beat-IIIa/IIIb/IIIc	*beat-IIIc1*
A0A6I8T6T3	LOC5575663	318	Beat-IIIa/IIIb/IIIc	*beat-IIIc2*
Q16T38	LOC5573307	323	Beat-IV	*beat-IVa*
A0A6I8T3Q5	LOC5564316	389	Beat-IV	*beat-IVb*
A0A6I8TBC6	LOC5571213	314	Beat-Va/Vb/Vc	*beat-Va*
A0A903UJK4	LOC5579262	303	Beat-Va/Vb/Vc	*beat-Vb*
A0A1S4G0B0	LOC5578571	372	Beat-VI	*beat-VI*
Q16UX7	LOC5572379	295	Beat-VII	*beat-VII*
A0A6I8TZ84	LOC5565349	824	Side	*side*
A0A6I8TMK3	LOC5571423	895	Side-II	*side-IIa*
A0A6I8T6R7	LOC5573643	975	Side-II	*side-IIb*
A0A6I8U3A5	LOC5572620	901	Side-II	*side-IIc*
A0A6I8TGL9	LOC5569913	1461	Side-III	*side-III*
A0A903V9A0	LOC5570293	996	Side-IV	*side-IV*
A0A6I8U5E3	LOC5566683	960	Side-V	*side-V*
A0A6I8T4J7	LOC5569257	979	Side-VI	*side-VI*
A0A903TT64	LOC5574581	929	Side-VII	*side-VII*
A0A6I8U0C6	LOC5576331	1062	Side-VIII	*side-VIII*
***O. biroi***				
A0A026WLQ9	LOC105277757	303	Beat-Ia/Ib/Ic	*beat-I*
A0A3L8E3G8	LOC105281146	269	Beat-IIa/IIb	*beat-II*
A0A3L8D839	LOC105278748	296	Beat-IIIa/IIIb/IIIc	*beat-IIIa*
A0A3L8DA76	LOC105278750	296	Beat-IIIa/IIIb/IIIc	*beat-IIIb*
A0A3L8D610	LOC105284227	340	Beat-IIIa/IIIb/IIIc	*beat-IIIc*
A0A3L8DQF1	LOC105285218	269	Beat-IV	*beat-IV*
A0A3L8DGH2	LOC105275676	286	Beat-Va/Vb/Vc	*beat-V*
A0A3L8E4A2	LOC105279130	290	Beat-VI	*beat-VIa*
A0A3L8E121	LOC105282898	311	Beat-VI	*beat-VIb*
A0A3L8DUP0	LOC105278517	287	Beat-VII	*beat-VII*
A0A3L8D925	LOC105275893	795	Side	*side*
A0A026WW65	LOC105288222	806	Side-II	*side-II*
A0A026WJB3	LOC105278806	856	Side-III	*side-III*
A0A3L8E1Y0	LOC105274944	920	Side-IV	*side-IVa*
A0A026WV55	LOC105274945	836	Side-IV	*side-IVb*
A0A3L8D7Y1	LOC105275896	802	Side-IV	*side-IVc*
A0A3L8D540	LOC105286893	980	Side-VI	*side-VI*
A0A026X112	LOC105280531	941	Side-VII	*side-VII*
A0A3L8DGW7	LOC105287868	1010	Side-VIII	*side-VIII*

Interestingly, recent single-cell RNA-seq reports from clonal raider ant ORNs revealed an unusual mode of OR gene regulation in which multiple OR transcripts can be detected within individual ORNs. In detail, ORNs that share a more similar transcriptome, and thus are clustered together, usually express *ORs* located in a tandem genomic array. In each ORN, transcription is initiated from a selected upstream start site and can extend through multiple downstream OR genes in the array, while only the most upstream OR mRNA in the transcript is translated into a functional protein [92]. As a result, ORNs within the same transcriptomic cluster can express different functional OR proteins despite sharing highly similar overall gene expression profiles. Thus, the ORNs are transcriptionally similar at the global transcriptome level but heterogeneous in their functional OR identity. To zoom in on ORN classes within each single-cell RNA-seq cluster, we plotted *beat/side* expression and the *ORs* of the tandem expressed in the cluster of each single cell as heatmaps (S12E Fig). We found that each ORN class, defined by the expression of the most upstream *OR*, exhibits class-specific expression of *beat/sides*. For example, in cluster 3, the ORN class defined by the functional expression of Or5-9E109 doesn’t express the ant *side-IVb,* whereas other ORN classes functionally expressing the adjacent Ors, Or5-9E108, Or5-9E107, and Or5-9E110 appear to be positive for this gene (S12E Fig). Similarly, ORN classes defined by the functional expression of Or5-9E151 or Or5-9E150 in cluster 14 don’t express the ant *beat-VII,* whereas many other ORN classes in this cluster do (S12E Fig). While these differences are not absolute, these observations raise the possibility that, upon OR tandem choice, additional developmental mechanisms may couple *OR* transcription initiation and *beat/side* expression specification in the clonal raider ant.

## Discussion

In this study, we comprehensively delineated the expression pattern of *beat/side* family genes in ORNs and PNs at single-cell and single-class levels. We found that each ORN or PN class exhibits a unique combinatorial expression of *beats/sides*, and this *beat/side* profile appears to be specified by the lineage-intrinsic genetic programs. In addition to analyzing the single-cell transcriptomes of ORNs and PNs, we also generated *MiMIC*-based gene trap driver lines to probe the *beat/side* expression across ORNs and PNs in vivo. This approach enabled mapping the *beat/side* expression to additional ORN/PN types not covered in the single-cell RNA-seq datasets. Collectively, we found that *beat/side* profiles can diverge between some closely related ORNs or some closely related PNs, suggesting that the Beat/Side combination may add variability to cell-surface codes, thereby biasing their glomerular targeting. We also investigated one interacting pair, Beat-IIa and Side-IV, based on their matching expression pattern between partner ORNs and PNs, and found no apparent glomerular mistargeting in knockdowns of *Beat-IIa* in ORNs or *Side-IV* in PNs. Yet, knockdown of the *side* gene in ORNs resulted in diminished synapses in the antennal lobes, suggesting a role in synapse development. Moreover, we found evolutionarily conserved expression patterns and ORN-specific combinatorial signature for *beat* and *side* orthologs in mosquitoes and ants, suggesting the shared genetic programs that establish the *beat/side* profile. In sum, our studies implicate ORN/PN class-specific combinatorial Beat and Side protein expression and their roles in olfactory circuit assembly.

### Developmental regulation of *beat/side* expression in ORNs and PNs

We found that the lineage-correlated expression profile of *beats/sides* in ORNs and PNs is a general principle, suggesting that lineage-specific programs set the *beat/side* combinatorial expression. Our genetic labeling results and single-cell RNA-seq analyses also reveal both shared and divergent expression patterns of *beat/side* between closely related ORNs. We found that some sibling ORNs have different *beat/side* profiles and project to distant antennal lobe regions, while some maintain the putative “template” combinations found in many ORNs and project to neighboring or close glomeruli. Adjacently born PNs can also have shared or divergent *beat/side* expression profiles. We therefore propose that: (1) at the higher level of the developmental hierarchy, i.e., in ORN sensillar type decisions or PN lineage specification decisions, the *beat/*side “template” is set by lineage-specific factors; (2) the latest common precursor of ORNs (i.e., sensilla subtype precursors) or PNs may have diverse intrinsic factors that bias the expression of additional *beat/side* combinatorial expression while some do not; (3) such that following the final terminal selection decision, Beat/Side combinations may mediate the synapse recognition and formation programs in closely related ORNs or PNs. Moreover, expression of additional cell-surface proteins can increase the cell-surface protein repertoire, further diversifying neuronal identification tags displayed on neurons utilized during circuit assembly. This two-step model, setting the lineage-specific cell-surface molecular template, followed by within-lineage diversification, seems to be an efficient way to establish the cell-type-specific surface molecular repertoire by genetically deterministic programs. This might be a developmental strategy to coordinate the combinatorial expression of duplicated paralogs.

The two-step model is conceptually supported by other highly diversified neuronal recognition systems, including clustered protocadherins (cPCDH) in mammals and Dscam in fruit flies. Both systems generate enormous molecular diversity through combinatorial paralog expression or extensive alternative splicing and were initially thought to rely largely on stochastic expression mechanisms. However, more recent studies suggest that lineage-dependent developmental programs can bias or propagate specific molecular identities. Neurons with closer developmental origin tend to share significantly more similar cPCDH or Dscam1 isoforms, which further drives homophilic binding-mediated repulsion to finely pattern the mouse neocortex and *Drosophila* medulla columns, respectively [95,96]. Together, these findings support a hierarchical strategy in which lineage-dependent molecular templates are subsequently diversified to establish neuronal identity and circuit specificity.

### Molecular complexity and redundancy of Beats/Sides as cell-surface “codes”

Side was first identified as the postsynaptic ligand, expressed in muscles, to attract motor neurons. Motor neurons expressing the receptor Beat-Ia follow the Side-labeled muscles and leave axonal fasciculations to innervate muscle targets [48–52]. The embryonic expression pattern analysis expanded to other Beat/Side family paralogs also suggests that Beats are neuronal receptors for Sides expressed in peripheral tissues [47]. For example, Side-VI is expressed in muscle fibers while its receptors, Beat-Vs, are expressed in motor neurons [47]. This evidence jointly indicates that Sides are present primarily at postsynaptic sites, signaling to presynaptic Beats to establish recognition and promote synaptogenesis. Outside of the neuromuscular junction, recent reports in the fly visual circuits also suggest presynaptic Side-II and postsynaptic Beat-VI mediate synaptic recognition [57]. Additional studies revealed that presynaptic (photoreceptor-expressed) Side-IV can induce synaptogenesis by acting as both ligands and receptors when interacting with postsynaptic Beat-IIa/b, forming synaptogenic complexes through interactions with Beat-IIa/b and the coreceptor Kirre [58].

In the olfactory circuits consisting of ORNs and PNs, we found that *beat* and *side* genes are expressed in the same neurons. ORNs generally lack Beats but are abundant for Sides. This would point to a model where Sides primarily interact with PN-expressed Beats. However, ORNs do express a few Beats with their binding partner Sides as well as some orphan Beats. And PNs generally express both Sides and Beats. Similarly, DIPs and Dprs can also be expressed in the same cells, like adult ORNs [29] and larval motor/sensory neurons [33]. There are several possible functional explanations based on Beat and Side interactions. First, some Sides and Beats may have different subcellular localizations that position places of protein-protein interactions and cellular functions. For example, Beats might localize to PN dendrites to interact with Sides on ORN axon terminals, while Side proteins localized to PN axon terminals may interact with Beats expressed on the dendrites of third-order neurons. And this asymmetric subcellular preference may vary in different cell types to mediate interactions with other neurons. In motor neurons, Beats are preferentially sorted to axon terminals, whereas in sensory neurons, some Sides are preferentially sorted to axon terminals. Notably, a recent work found that in Kenyon cells of the mushroom body that receive inputs from olfactory PNs, many IgSF genes, including most *beats*, are not expressed, whereas many *sides* are [97]. This raises the possibility that some Beat proteins in PNs might also act on axon terminals to interact with postsynaptic Kenyon cells, while some Side family proteins are also required on the PN dendrites during ORN-PN matching. Second, Beats/Sides can function in both dendrites and axons simultaneously and act as ligands and receptors bidirectionally. This points to a model in which the combinatorial code of multiple surface proteins establishes synaptic specificity recognition, likely by providing differential adhesive forces. Indeed, we observed non-ORN signals for Side-VI and Beat-Va proteins in the antennal lobe, likely reflecting their colocalization on PN dendrites (S9C and S9F Fig). Third, Beats and Sides in the same neuron might interact *in cis* on the same membrane in addition to their interactions *in trans* with Beat/Side proteins on other neurons. Interestingly, a recent study showed that DIP-Dpr interactions favor *cis* over *trans* when present in the same cell [98]. It might also be the case that when Beats/Sides are localized together in the same membrane regions, it is the competition between *cis* and *trans* interactions with all present binding partners in the proximity that sets the “net” Beat/Side surface codes.

Additionally, combinations of cell-surface adhesion molecules can form multimeric complexes, so the absence of any component may not abolish but merely reduce the recognition efficacy or shift these complexes to new stoichiometric configurations [46,99]. Given this sophisticated context, it is not surprising that removing a single gene is insufficient to change glomerular organization. Regarding Beat/Side combinatorial complexity, deleting *side-IV* in PNs or *beat-IIa* in ORNs does not ambiguate DM1 from adjacent glomeruli (Fig 4), and ORN-PN synaptic matching can still occur. Thus, the promiscuous and redundant functions of the Beat/Side family have the capacity to form an error-tolerant adhesion code, enabling recognition robustness. These are supported by our efforts to perturb a single *beat/side* gene that caused subtle to no defects (S10A Fig). Previously reported screens for CSMs mediating ORN-PN synaptic matching also didn’t report any defects in glomerular targeting with any *beat/side* knockdowns [17]. A recent study has shown that collective manipulation of multiple cell-surface molecules substantially rewires an ORN-PN circuit, while loss-of-function of a single molecule causes only minor mistargeting [100,101]. Our expression dataset provides a roadmap for further cracking the combinatorial coding nature of Beats and Sides through multiplexed perturbation. For example, DM1 and DM2 glomeruli are adjacent to each other; DM2 ORNs might distinguish themselves from DM1 ORNs by Side-VI, which interacts with Beat-Va/Vc expressed by DM2 PNs, whereas these interactions don’t exist between DM1 ORNs and PNs (Fig 4H and 4I). In parallel, DM1 ORNs express the additional molecule Beat-IIb, potentially strengthening their interactions with DM1 PNs relative to DM2 ORNs (Fig 4H and 4I). Together, these differential combinatorial interactions may help reinforce the specificity of ORN–PN matching between neighboring glomeruli. Accordingly, combinatorial manipulations that disrupt these biased recognition cues could potentially lead to miswiring between DM1 and DM2 ORN–PN pairs.

Intriguingly, we observed weakened synapses in the antennal lobes when we knocked down the *side* gene*,* which suggests that Side and Beat protein interactions might mediate synapse formation in the olfactory circuits. This is supported by other recent studies in the visual system reporting Side-IV as a synapse-inducing protein [58] and that Beat-VI expression gradients regulate synaptic density gradients through their interactions with Side-II [45]. These results suggest that Beat/Side interactions may inherently have synaptogenic functions and participate in the subglomerular synaptic organization during olfactory circuit development. Furthermore, since many *beats/sides* show persistent high expression levels from the pupal stage to adulthood, they might be recruited for synapse induction, formation, and stabilization, and modulated by OR signaling and/or neuronal activity.

### Evolutionarily conserved expression of *beats/sides* in ORNs of insects

We observed similar combinatorial expression patterns across ORN clusters among recently duplicated paralogs within the same species, as well as orthologs in ants and mosquitoes, suggesting evolutionarily conserved mechanisms that regulate their expression in ORNs. We speculate that ancestral paralogs of *beats/sides* with their regulatory elements acquired cell-type-specific expression patterns, which were generally retained during evolution and shared between species. More recent gene duplication events after speciation generated additional paralogs within each *beat/side* clade, where we observe both shared (*Drosophila beat-IIIa/b/c* and *beat-Va/b/c*, *A. aegypti beat-IVa/b* and *side-IIa/b*, *O. biroi beat-VIa/b*) and divergent expression patterns (*A. aegypti beat-Ia1/a2* and *side-IIb/c*, O*. biroi side-IVa/b/c*). Though some *beat* and *side* genes exhibit evolutionary plasticity in their expression patterns, conservation of the expression of many *beat/side* genes in ORNs appears to be a general rule. This implies that there are selection pressures on the operative roles that these genes play in the ORN development and function.

Interestingly, in clonal raider ants, there appear to be two steps specifying the ORN fate: first, choosing the OR tandem and second, choosing the OR transcription start site within the tandem. Our results suggest that the *beat/side* ortholog expression is also coupled with these two steps. *Drosophila* appears to exploit the genetically deterministic strategy to specify the combinatorial expression of cell-surface proteins to organize diverse ORN classes into the hardwired circuits. However, when neuronal types become more heterogeneous, possibly exceeding a diversity limit, such a solely genetics-dependent program may be incapable. This is likely to be the case in the olfactory systems of ants with many more classes of ORNs. Recent studies suggest ORs themselves may play instructional roles in glomerular targeting in ants, different from fruit flies but similar to mammals [94,102–105]. It is possible that the ORN class-specific expression of *beat/side* is also regulated by ORs in ants.

### Limitations of the study

Many *beat/side* genes transcribe multiple splice isoforms, which adds an additional layer of molecular diversity. This study didn’t examine the expression of *beat* and *side* splice isoforms in the olfactory system. Our transgenic *GAL4* driver lines generally trap the common regions across isoforms and cannot address isoform-specific expression. Single-cell RNA-seq approaches that resolve *beat* and *side* splice isoforms or expression analysis of isoform-specific *T2A-GAL4*s would be needed to this end.

This study only reveals the *beat* and *side* expression at the transcriptional level and does not address the Beat and Side protein-level distributions. Some studies reported large discrepancies between transcripts encoding surface molecules and their protein abundance in developing PNs [78], suggesting regulation at the level of translation, protein stability, modification, sorting, and delivery to the cell surface.

This study did not investigate the expression of *beat/sides* in local interneurons, which also participate in the olfactory circuit and form synaptic connections with both ORNs and PNs within the antennal lobe. Our expression data do suggest that some of them are likely expressed in local interneurons. Several *GAL4-*driven membrane reporters label the neurite structures resembling the local interneuron features in the antennal lobe (*beat-Va, beat-VI*, Figs 1D and S9C). Additionally, *beat-Va* is not expressed in either ORNs or PNs targeting the DM1 glomerulus (Figs 2B and 3B), but the Beat-Va protein signal is still detectable in this region (S9C Fig), suggesting its localization to the local interneurons innervating this glomerulus.

### Future directions

In the future, it will be interesting to investigate how regulatory sequence evolution contributes to the conserved and divergent expression patterns of *beat* and *side* paralogs across species. Additional biochemical characterization of Beats/Sides protein-protein interactions in different insect species will provide novel insights into the co-evolution of coding sequences with regulatory sequences to reconcile their functional evolution in circuit assembly.

Understanding the biochemical features of Beat and Side proteins will greatly inform the functional analysis in the circuit assembly context. This includes developing a more sensitive and specific test to deorphanize Beat-Side interactions, characterizing whether Beats/Sides are transmembrane proteins or membrane-anchored like DIPs/Dprs [106], and testing whether they interact *in trans* and/or *in cis*. If they are transmembrane proteins, then how are their functions mediated by intracellular and extracellular protein domains? Proximity labeling-based proteome profiling, like BIO-ID, can identify these protein interactors, which will inform further functional investigation. A recent study reported that the N-terminal domain of Beat-Ia is localized to the cell surface while the C-terminus accumulates in the nucleus; this implies that Beat-Ia might undergo proteolytic cleavage for proper function [55]. Future biochemical, genetic, and neurophysiological studies will help reveal the function of Beat and Side proteins in recognition specificity and synaptic transmission.

## Materials and methods

### *Drosophila* stocks and genetics

Flies were raised in classic molasses media provided by Archon Scientific. Most crosses were kept at room temperature (23 °C), except the RNAi experiments performed at 28 °C to maximize the knockdown efficiency. For RNAi experiments, male and virgin female flies were mixed at room temperature for three days to facilitate mating. Then flies were raised at a 28 °C incubator until 5–7 days after eclosion before dissection. The transgenic *Drosophila melanogaster* strains used in this study are listed in Table 1. BDSC, Bloomington Drosophila Stock Center; VDRC, Vienna Drosophila Resource Center. The full genotypes related to each figure can be found in S1 Table.

**Table 1:** References and identifiers of the transgenic flies used in this study.

Genotype	Reference	Identifier
*hs-cre, vas-phiC31* (chrX)	[63]	BDSC 60299
*Trojan-Gal4.0; Dr/TM3*	[63]	BDSC 60301
*Sp/CyO; Trojan-Gal4.0*	[63]	BDSC 60302
*Trojan-Gal4.1; Dr/TM3*	[63]	BDSC 60304
*Sp/CyO; Trojan-Gal4.1*	[63]	BDSC 60305
*Trojan-Gal4.2; Dr/TM3*	[63]	BDSC 60307
*Sp/CyO; Trojan-Gal4.2*	[63]	BDSC 60308
*10XUAS-IVS-myr.GFP attP40*		BDSC 32198
*10XUAS-IVS-myr.GFP attP2*		BDSC 32197
*20xUAS-6xGFP VK00018*		BDSC 52261
*beat-Ia-GAL4 [MI11181]* chr2	This study	N.A.
*beat-Ib-T2A-GAL4 [MI02527]* chr2	This study	N.A.
*beat-Ic-GAL4 [MI03347]* chr2	This study	N.A.
*beat-Ic-T2A-GAL4 [MI01467]* chr2	This study	N.A.
*beat-IIa-T2A-GAL4 [MI11485]* chr3	[107]	BDSC 76215
*beat-IIb-T2A-GAL4 [MI03102]* chr3	This study	N.A.
*beat-IIIa-T2A-GAL4 [MI14565]* chr2	This study	N.A.
*beat-IIIb-T2A-GAL4 [MI13179]* chr2	[107]	BDSC 76227
*beat-IIIb-T2A-GAL4 [MI15124]* chr2	This study	N.A.
*beat-IIIc-T2A-GAL4 [MI03726]* chr2	This study	N.A.
*beat-IIIc-T2A-GAL4 [MI09457]* chr2	This study	N.A.
*beat-IV-T2A-GAL4 [MI13984]* chr3	This study	N.A.
*beat-IV-T2A-GAL4 [MI05715]* chr3	This study	N.A.
*beat-Va-T2A-GAL4 [MI00191]* chr3	This study	N.A.
*beat-Vc-T2A-GAL4 [MI08482]* chr3	This study	N.A.
*beat-VI-GAL4 [MI13,252]* chr3	This study	N.A.
*beat-VII-T2A-GAL4 [MI07880]* chr3	This study	N.A.
*side-T2A-GAL4 [MI03741]* chr3	This study	N.A.
*side-II-T2A-GAL4 [MI07122]* chr2	This study	N.A.
*side-III-GAL4 [MI01491]* chr3	This study	N.A.
*side-IV-GAL4 [MI10049]* chr3	This study	N.A.
*side-V-T2A-GAL4 [MI04721]* chr2	This study	N.A.
*side-VI-T2A-GAL4 [MI03149]* chr3	This study	N.A.
*side-VI-T2A-GAL4 [MI12999]* chr3	This study	N.A.
*side-VII-GAL4 [MB10368]* chr3		BDSC 29116
*side-VIII-T2A-GAL4 [MI04810]* chr2	This study	N.A.
*ey-FLP* (chrX)	[108]	BDSC 5580
*GH146-FLP* (chr2)	[18]	BDSC 81291
*UAS>STOP>mCD8.GFP* (chr2)	[18]	BDSC 30125
*UAS>STOP>mCD8.GFP* (chr3)	[18]	BDSC 30032
*beat-Ib* ^*MI02527-GFP*^ */SM6a*		BDSC 60507
*beat-VI* ^*M03969-GFP*^ */TM3*		BDSC 61773
*beat-VII* ^*MI02091-GFP*^ */TM6c*		BDSC 60178
*side-III* ^*MI01139-GFP*^ */TM6c*		BDSC 60162
*beat-IIb* ^*MI03102-GFP*^		BDSC 59406
*beat-IV* ^*MI05715-GFP*^		BDSC 66506
*beat-Va* ^*MI00191-GFP*^		BDSC 60146
*side* ^*MI00149-GFP*^		BDSC 60507
*side-V* ^*MI04721-GFP*^		BDSC 63174
*side-VI* ^*MI01052-GFP*^		BDSC 60515
*peb-GAL4* (chrX)	[109]	N.A.
*GH146-GAL4* (chrX)		BDSC 91812
*UAS-Dcr* (chrX)		BDSC 58726
*Or42b-mCD8.GFP* (chr2)		BDSC 52648
*Or92a-mCD8.GFP* (chr2)		BDSC 52646
*Or47a-syt.GFP, Or47b-syt.GFP, Gr21a-syt.GFP* (chr2)	Volkan lab stock	N.A.
*UAS-Luc RNAi attP2*		BDSC 31603
*attP40*		BDSC 36304
*UAS-myr.tdTomato attP40*		BDSC 32222
*UAS-RFP RNAi attP2*		BDSC 35785
*UAS-beat-Ia RNAi attP40*		BDSC 64938
*UAS-beat-Ia RNAi GD* (chr2)		VDRC 4544
*UAS-beat-Ib RNAi attP40*		BDSC 57157
*UAS-beat-Ib RNAi KK* (chr2)		VDRC 101662
*UAS-beat-IIa RNAi attP2*	[88]	BDSC 28072
*UAS-beat-IIb RNAi attP40*		BDSC 57157
*UAS-beat-IIb RNAi KK* (chr2)		VDRC 104935
*UAS-beat-IIIa RNAi attP40*		BDSC 64526
*UAS-beat-IIIa RNAi GD* (chr2)		VDRC 45866
*UAS-beat-IIIb RNAi attP40*		BDSC 56984
*UAS-beat-IIIb RNAi KK* (chr2)		VDRC 107521
*UAS-beat-IIIc RNAi attP40*		BDSC 50941
*UAS-beat-IIIc RNAi KK* (chr2)		VDRC 109015
*UAS-beat-IV RNAi attP40*		BDSC 56981
*UAS-beat-VII RNAi attP40*		BDSC 60056
*UAS-beat-VII RNAi KK* (chr2)		VDRC 102329
*UAS-side RNAi attP2*		BDSC 50642
*UAS-side-II RNAi KK* (chr2)		VDRC 107512
*UAS-side-III RNAi KK* (chr2)		VDRC 103669
*UAS-side-IV RNAi GD* (chr2)	[58]	VDRC 16636
*UAS-side-V RNAi attP40*		BDSC 61953
*UAS-side-VI RNAi KK* (chr2)		VDRC 103456
*UAS-side-VII RNAi KK* (chr2)		VDRC 106353
*UAS-side-VIII RNAi attP40*		BDSC 62897
*UAS-side-VIII RNAi KK* (chr2)		VDRC 104814
*UAS-SynLight attP2*	[89]	BDSC 602367
*UAS-TNT (-)* (chr2)	[110]	BDSC 28840
*2xUAS-EKO* (chr2)	[111]	BDSC 40974
*UAS-Cas9.C attP2*		BDSC 54595
*U6-QUAS gRNA attP40*		BDSC 67539
*U6-beat-IIIc gRNA attP40*	Gift from Yu-Chieh David Chen	N.A.
*U6-side gRNA attP40*	Gift from Yu-Chieh David Chen	N.A.
*U6-side-II gRNA attP40*	Gift from Yu-Chieh David Chen	N.A.
*U6-side-III gRNA attP40*	Gift from Yu-Chieh David Chen	N.A.
*U6-side-IV gRNA attP40*	Gift from Yu-Chieh David Chen	N.A.
*U6-side-V gRNA attP40*	Gift from Yu-Chieh David Chen	N.A.
*U6-side-VI gRNA attP40*	Gift from Yu-Chieh David Chen	N.A.
*U6-side-VII gRNA attP40*	Gift from Yu-Chieh David Chen	N.A.
*U6-side-VIII gRNA attP40*	Gift from Yu-Chieh David Chen	N.A.
*UAS-beat-IIa gRNA+beat-IIb gRNA attP40*	Gift from Matthew Y Pecot	N.A.
*UAS-beat-Ib 86FB*	FlyORF	F002855
*UAS-beat-Vc 86FB*	FlyORF	F004239
*UAS-beat-VI 86FB*	FlyORF	F002906

### Generating *T2A-GAL4* transgenic flies from *MiMIC* lines

We used the in vivo genetic cross-based method [63] to swap the *T2A-GAL4* construct with the *MiMIC* cassette. Briefly, by multiple crosses, the dual-recombinase (*hs-cre* + *vas-phiC31*) helper component, the in-frame *T2A-GAL4* donor component, and the target *MiMIC* locus in the genes of interest were introduced together into one parental animal, where the *T2A-GAL4* donor sequence was excised out by Cre recombinase and inserted into the *MiMIC* docking site by germline-expressed phiC31 integrase. When this parental animal was crossed to the *UAS-myr.GFP* reporter line, the recombinant progeny would have GFP expression driven by the *MiMIC-GAL4* if *T2A-GAL4* was inserted in the correct frame and orientation. The recombinant was crossed with a double-balancer line to make a stable stock. The flanking sequence of the insertion was later amplified by PCR and sequenced for verification. Specifically, we also found that the *T2A-GAL4* construct could be used in any 5′ UTR-located *MiMIC* sites in a frame-independent manner. In this case, the *GAL4* coding sequence with ATG start codon in the 5′ UTR would hijack the translation of the native gene as an upstream open reading frame, and the *linker-T2A* sequence could be neglected. As the *GAL4* open reading frame is within the whole transcript of the native gene, it is still expected to faithfully represent the transcription levels and patterns of the native gene. We name the driver line *gene-GAL4* if *GAL4* is within 5′ UTR of the host gene (Table 1). For example, we generated two lines trapping *beat-Ic*, one inserted in 5′ UTR (MiMIC03347) and one inserted into a coding intron (MiMIC01467), and they label the same population of PNs (S1D Fig).

### Whole animal fluorescence imaging of *beat/side* expression

The whole animal fluorescence imaging was performed on an Olympus BX51WI upright scope equipped with a C11440-36U camera. Larvae and adults were killed by 70% ethanol first and mounted on slides without coverslips. Pupae were directly mounted on slides without coverslips. Larvae and pupae were dorsal side up, whereas adults were mounted with the lateral side up. Images were acquired under blue light and the GFP channel, with manually adjusted exposure time.

### Immunohistochemistry

Flies were first killed with 70% ethanol. Then, brains were dissected in PBST buffer (0.2% Triton X-100 in 1X PBS), fixed in 200 μL centrifuge tubes with 4% paraformaldehyde for 30 min, followed by three 10-minute washes in PBST. Primary antibody mix (150 μL) was then added to the tubes, incubating brains on the orbital shaker at 4 °C overnight. Brains were washed three times for 20 min each with PBST at room temperature before secondary antibody staining. Secondary antibody mix (150 μL) was then added to the tubes, and the brains were incubated on the orbital shaker at 4 °C overnight. Brains were washed three times for 20 min each with PBST at room temperature again before being mounted on the imaging slides. Natural goat serum (1%) was added to the primary and secondary antibody mix for blocking. The following primary antibodies with dilution ratios were used: rabbit anti-GFP (Invitrogen, 1:1,000), rat anti-Ncad (DSHB, 1:20), rabbit anti-DsRed (TaKaRa Bio, 1:250); the following secondary antibodies with dilution ratio were used: Alexa Fluor 488 goat anti-rabbit IgG (Invitrogen, 1:1,000), Alexa Fluor 647 goat anti-rat IgG (Invitrogen, 1:200), Alexa Fluor Cy3 goat anti-rabbit IgG (Invitrogen, 1:200). Both primary and secondary antibody cocktails were diluted in PBST.

Specifically, for Brp synaptic analysis, we only stained RFP (mStraw) and Ncad without staining GFP for two reasons. First, the transgenic mStraw fluorescence of the synaptic marker is very low and needs a very high laser power to detect the signal, while the natural GFP fluorescence is bright enough. Second, because we later normalized the RFP intensity to the GFP intensity as the proxy for synaptic density in the region of interest, no staining of GFP can reduce the variability introduced during immunohistochemistry.

### Confocal imaging

To prepare brain samples for imaging, a mounting solution (Flouromount-G, SouthernBiotech) was added to the brains on the glass slide before the cover slip was mounted. Confocal images were acquired by either an Olympus Fluoview FV1000 microscope or a Zeiss 880 microscope with a 40× or 60× objective lens. Brains were scanned through the Z-axis from the posterior side to the most anterior side of the antennal lobes. For *beat/side-GAL4*-based glomerular innervation analysis, imaging parameters were adjusted across different *GAL4* drivers to highlight some glomeruli with very low expression (see details below). For phenotypic determination, imaging parameters were kept consistent across the experimental and control groups.

### *beat/side* glomerular expression pattern analysis of *beats/sides*

For *ey-FLP*-induced labeling of each *beat/side*-expressing ORN class, we found *ey-FLP* expression appears leaky in germline and thus crossing *ey-FLP* first with *UAS>STOP>mCD8.GFP* may lead to the removal of the *STOP* sequence constitutively and thereafter fail to restrict the GAL4-driven GFP expression solely in ORNs. To overcome this issue, we first crossed each *GAL4* driver line to the *ey-FLP* line and then crossed to the *UAS>STOP>mCD8.GFP* conditional reporter line. For *GH146-FLP*-induced labeling of each *beat/side*-expressing PN class, each *GAL4* driver line can be directly crossed to the *GH146-FLP*; *UAS>STOP>mCD8.GFP* line. For several *MiMIC-GAL4-*labeled brains, we examined both female and male brains, but didn’t find any salient discrepancies between sexes. So only the results from female animals were reported in this paper.

As we aimed to clearly determine whether each glomerulus is positive or negative for each gene, we didn’t use the constant parameters to acquire the images for different *GAL4* driver lines. We adjusted the acquisition parameters in order to clearly reveal glomeruli with very low expression. After scanning each brain from the most posterior end to the most anterior end of the antennal lobe, we referred to the previously characterized glomerular map [6] and manually determined the glomerular identity and the corresponding *beat/side* expression (S4 Fig), assigning 0 to negative expression and 1 to positive expression. At least three brains were examined for each *GAL4* driver line. Notably, for the *GH146-FLP-*based labeling, we occasionally found inconsistent glomerular innervation patterns between individuals and even right and left lobes of the same brain (S4F Fig). This is likely due to the incomplete excision of the *STOP* cassette in a subset of PNs. As each glomerulus could be innervated by as few as one PN, it is possible that some glomeruli are not labeled in an antennal lobe. We thus determined these glomeruli to be positive if we observed the GFP signal for that glomerulus in two or more antennal lobes.

Given that inconsistent imaging parameters were used between different driver lines, quantifying and comparing the gene expression level of a particular glomerulus across different genes based on the fluorescence intensity is not appropriate. However, for each individual gene (i.e., the same *GAL4* driver line) consistent imaging settings were kept across brains, allowing relative comparison of expression levels between glomeruli for that gene. We therefore also adopted a semi-quantitative scoring approach in which the expression level of each gene in each glomerulus was binned into four discrete categories—no, low, medium, and high expression—based on *GAL4*-labeled fluorescence intensity. We assigned values of 0, 1, 2, and 3, respectively. This approach was chosen for two reasons. First, fluorescence intensity measurements can be influenced by variability in staining quality, brain mounting, tissue morphology, and segmentation accuracy, making precise grayscale-based quantification less robust across samples. Second, we also found the presence of occasional outlier glomeruli with exceptionally strong fluorescence signals (e.g., V glomerulus ORNs for *beat-IIIc* and *side-III*, VL1 glomerulus ORNs for *side-IV*, Fig 2A). Normalizing expression values to the brightest glomerulus for each gene would compress the dynamic range of the remaining glomeruli, causing many moderately or robustly labeled glomeruli to be represented by disproportionately small normalized values. As a result, continuous intensity-based normalization could distort the relative expression spectrum and reduce the interpretability of biologically meaningful differences between glomeruli. Binning expression into discrete levels instead preserves overall and reproducible relative differences in expression while reducing sensitivity to technical variability and extreme outliers. Notably, for three genes with regard to the ORN expression map, *beat-Vc*, *beat-Ic*, *beat-VI*, even the most strongly labeled glomeruli exhibit substantially lower fluorescence intensity compared with the typical expression levels observed for other genes; therefore, to preserve the relative expression scale across the dataset, we did not assign any glomeruli for these genes to the “high” expression category and instead classified them only as medium or low expression. The binary expression matrix and the binned matrix were then input into the downstream hierarchical clustering analysis using the heatmap.2 function with the default settings in R (based on the pairwise Euclidean distance). The ORN and PN expression matrices can be found in S4 Data.

Specifically, to determine whether a gene is in the ORN lineage-specific putative template, we calculated its mean expression across ORN classes in each lineage. If the mean expression is equal to or greater than 0.6, this gene is considered present in the default template; If the mean expression is equal to or less than 0.4, this gene is considered to be absent in the default template. For the genes with mean expression between 0.4 and 0.6, this suggests that nearly half of ORN classes in that lineage express the gene (e.g., 5/9 of AC ORN classes express *beat-IIb*), and this gene could be present or absent in the default template with comparable likelihood.

### Phenotype quantification

The glomerular phenotype was measured as the proportion of antennal lobes showing morphological or positional abnormalities across all studied brains in each group, compared with the controls. The *P* value was determined using the two-tailed Fisher’s exact test, using the integrated features in the GraphPad Prism 9.

To quantify synaptic density at the gross antennal lobe level, we first obtained the Z-stack of confocal sections by maximum projection. Then, we manually selected the regions of interest (ROI) according to the Ncad channel, namely the right and left antennal lobes. We also selected a control region in the dorsal part of the brain where the driver *peb-GAL4* is not expressed. We then subtracted the average intensity of the RFP channel of the control region from that of the ROI. This is the adjusted mean RFP intensity of the ROI. We did the same thing to get the adjusted mean GFP intensity of the ROI. We finally calculated the ratio between the adjusted mean intensity of RFP and the adjusted mean intensity of GFP as the readout for each antennal lobe. An unpaired *t* test or multiple comparisons post-ordinary one-way ANOVA was used to compare the average RFP/GFP ratio between groups.

### Bulk tissue RNA-seq and single-cell RNA-seq analysis from previously published datasets for *Drosophila melanogaster*

The publicly available datasets used are listed in Table 2. For bulk tissue RNA-seq datasets, the author-processed datasets (expression matrec and differentially expressed gene analyses using either EdgeR or DESeq) were directly used for customized analyses and visualization in this study. For single-cell RNA-seq datasets, the author-annotated expression matrices were directly used for similarity analysis (see details below).

**Table 2:** References and identifiers of the sequencing datasets used in this study.

Dataset	Reference	Identifier
Antennal WT bulk RNA-seq of *Drosophila melanogaster*	[29]	GSE75986
PN WT bulk RNA-seq of *Drosophila melanogaster*	[78]	GSE140093
ORN single-cell RNA-seq of *Drosophila melanogaster*	[13,64]	GSE162121 GSE143038
PN single-cell RNA-seq of *Drosophila melanogaster*	[79,112]	GSE161228 GSE100058
*Amos* mutant vs. WT antennal RNA-seq of *Drosophila melanogaster*	[71]	PRJNA532415
*Atonal* mutant vs. WT antennal RNA-seq of *Drosophila melanogaster*	[70]	N.A.
*Or* mutant vs. WT antennal RNA-seq of *Drosophila melanogaster*	[90]	GSE179213
ORN single-cell RNA-seq of *Aedes aegypti*	[91]	GSE192978
ORN single-cell RNA-seq of *Ooceraea biroi*	[92]	SRX21504666, SRX21504667

### Pairwise similarity analysis for gene combinatorial expression in ORNs and PNs

The ORN single-cell RNA-seq dataset was first filtered to keep cells annotated to a single ORN class. Then, for cells in each stage, we extracted their *beat/side* expression profile, i.e., the vector of which each element is the expression value of each *beat/side* gene. We further filtered out the cells with zero expression for all *beat/side* genes. The remaining cells were used to calculate the expression correlation for *beat/side* genes and other gene combinations, e.g., IgSF- or CSM-encoding genes. We first calculated the Spearman’s correlation matrix for *beat/side* expression across the selected cells in each stage [96]. Then, we assigned the kinship for each cell-cell pair based on the annotated ORN/glomerular identity, sensillum where they are housed, and the sensillar type [6,64,113–117]. Specifically, if two cells are annotated as the same ORN class, this pair is defined as “within ORN class”; if two cells are annotated as different ORN classes but both in the same sensillar subtype, this pair is defined as “within sensillar subtype”; if two cells are annotated as different ORN classes from different sensillar subtypes, but belong to the common sensillar type, this pair is defined as “within sensillar type”; if two cells are annotated as different ORN classes from different sensillar types, the pair is defined as “between sensillar types”.

The PN single-cell dataset was first filtered to retain cells annotated to a single PN class (excluding a small subset of anterior paired lateral (APL) neurons) and their corresponding lineage identity. We only kept cells annotated to either adPN or lPN lineage (removing multiglomerular ventral PNs). Then, we extracted their *beat/side* expression vector for cells in each stage. We further filtered out the cells with zero expression for all *beat/side* genes. The remaining cells were used to analyze different gene combinations at the same stage. We first computed the Spearman’s correlation matrix of *beat/side* expression across the selected cells in each stage. Then, we assigned the kinship for each cell-cell pair based on the annotated PN/glomerular identities and lineage identities. Specifically, if two cells are annotated as the same PN class, this pair is defined as “same glomerulus, same lineage”; if two cells are annotated as different PN classes but in the same PN lineage, this pair is defined as “different glomeruli, same lineage”; if two cells are annotated as different PN classes from different PN lineages, this pair is defined as “different glomeruli, different lineages”. Cell–cell pairs belonging to “same glomerulus, different lineages” were rare and removed prior to statistical tests.

We finally plotted the distribution of the correlation coefficient of each kinship category and compared the mean between the two categories using the Mann–Whitney *U* test. This analysis was expanded to the correlation of IgSF and CSM genes, randomly selected genes, and the whole transcriptome (all genes) at each stage. As the number of pairs (*n*) in each kinship category vary dramatically, we also randomly selected the same number of pairs in each kinship category. Then, we performed statistical tests on the resampled data and found that the results also hold true. Therefore, all related figures in this paper were plotted based on the original data without resampling.

Shuffled controls in Figs 2F and 3F were calculated under the same pipeline as the abovementioned, except the annotations of each cell, i.e., ORN/PN class identity and corresponding lineage information were shuffled. We shuffled 1,000 times with different random seeds and plotted one example of each randomization in these figures.

A similar analysis, including the shuffled controls, was also applied to the *beat/side* genetic labeling results, using the binary and binned expression matrices as the input. Pearson’s correlation coefficient was computed between each cell type pair. In this case, as each glomerulus represents the whole ORN/PN classes, we would not have the “within ORN class” or “same glomerulus, same lineage” category.

### Ortholog identification and phylogenetic analysis of *beats* and *sides* in yellow fever mosquitoes and clonal raider ants

To identify the orthologs of *Drosophila melanogaster beat/side* genes in *Aedes aegypti* (yellow fever mosquitoes) and *Ooceraea biroi* (clonal raider ants), we used two ways to cross-validate the candidate list. For the database-guided way, we first queried the *Drosophila* genes one by one to get the putative orthologs in the other two species on https://www.orthodb.org [118]. Then, we queried the mosquito and ant genes to get the fruit fly orthologs. This reciprocal approach gave rise to a primary list of candidate *beats/sides* in mosquitoes and ants. We also did an unbiased homology search. We used the blastp tool on NCBI (https://blast.ncbi.nlm.nih.gov/Blast.cgi?PAGE=Proteins) to find the orthologous sequences encoded by the mosquito and ant genomes of *Drosophila* Beat-Ia and Side proteins. We applied additional filters, including protein size and AlphaFold-predicted structures (https://alphafold.ebi.ac.uk). We only kept candidates with comparable amino acid lengths to *Drosophila* homologs and similar protein structures (two Ig domains for Beats; five Ig domains and one fibronectin domain for Sides). This gave rise to a longer list of candidates. We compared these two lists and found that the first is contained in the second one. We then ran multiple sequence alignments of all Beat candidate and Side candidate proteins, together with the fruit fly proteins, to build the phylogenetic trees, respectively, using the Clustal Omega program [119] with the default parameters on https://www.uniprot.org. The multiple sequence alignment files and the phylogenetic trees can be found in S5 Data.

### Single-cell RNA-seq analysis of *beat/side* ortholog expression in the ORNs of yellow fever mosquitoes and clonal raider ants

Author-annotated single-cell RNA-seq datasets of *Aedes aegypti* and *Ooceraea biro* from two prior studies (Table 2) were directly used for downstream analyses and visualization. We extracted the mosquito and ant *beat/sides* genes from the original expression matrices. Based on the phylogenetic reconstruction described earlier, we renamed them to reflect their phylogenetic relationships with analogous genes in fruit flies (Table 3).

### Linear regression for the ORN expression breadth of *beat/side* orthologs between species

We defined the expression breadth as the percentage of ORN classes, i.e., ORN clusters in the single-cell RNA-seq datasets, that express the given gene (above the threshold). The threshold to produce the results in Figs 5G and S12E is 15%, 20%, and 20% cells expressing the gene (log-normalized expression >0.5) for the fruit fly, yellow fever mosquito, and clonal raider ant, respectively. These thresholds were determined based on the distribution of the fraction of positive cells across all clusters in each dataset. We also tested more stringent (25% for all) or more relaxed (10% for all) thresholds and observed that the high and significant correlations all hold true. The paralogs from more recent duplications in each clade, *beat-Ia*, *beat-Ib*, *beat-Ic*, for example, are averaged as *“beat-I”* for cross-species comparison.

### Statistics

R 4.3.1 and the necessary packages were used for all the abovementioned analyses and visualization. Statistical tests were performed using the built-in functions of R or through GraphPad Prism 9. The codes for statistics related to quantitative figure panels can be found in https://github.com/volkanlab/beats_sides_olfactory_circuit_Drosophila_Duan_PlosBio2026 and are archived in https://doi.org/10.5281/zenodo.21652764.

## Supporting information

S1 Data Underlying data for quantitative figure panels, related to Figs 2, 3, 4, 5, S3, S5, S7, S8, S10, S11, and S12. (XLSX)

S2 Data Underlying data for Figs 2E, 2F, and S3E. (ZIP)

S3 Data Underlying data for Figs 3E, 3F, and S7D. (ZIP)

S4 Data GAL4-labeling-based expression matrices of *beat/side* across glomeruli, related to Figs 2, 3, 4, S5, and S8. (XLSX)

S5 Data Multiple sequence alignment and phylogenetic tree of *beat* and *side* paralogs and orthologs across insect species. Related to Figs 5 and S12.
(ZIP)

S1 Table Full genotype of animals in each figure. (XLSX)

S1 Fig Consistent expression patterns across different *MiMIC* driver lines for the same gene. Related to Fig 1. **(A–E)** Representative images showing brains labeled by two different *MiMIC*-based gene trap drivers targeting the same gene. Scale bar in (A) applies to images in (A) to (D). Insertion site for these additional *MiMIC* swap lines: *beat-IIIb*^*MI15124*^: coding intron, targeting 2/2 isoforms; *beat-IIIc*^*MI09457*^: coding intron, targeting 2/2 isoforms; *side-VI*^*MI12999*^: coding intron, targeting 2/2 isoforms; *beat-Ic*^*MI01467*^: coding intron, targeting 3/3 isoforms; *beat-IV*^*MI05715*^: coding intron, targeting 2/2 isoforms.
(TIF)

S2 Fig *beat* and *side* expression are enriched in the nervous system throughout development, revealed by *MiMIC*-based gene trap drivers. Related to Fig 1. Whole-animal fluorescence images of each *beat/side* gene trap GAL4 driving myr.GFP in the 3rd instar larval stage, mid-pupal stage (~48 h APF), and adult.
(TIF)

S3 Fig Characterization of *beat/side* expression in the published datasets of bulk antenna and single-cell RNA-seq of ORNs. Related to Fig 2. **(A)** Dynamic *beats/side* expression in antennal tissues over development. Heatmap showing the temporal dynamic expression of *beats/sides* from third-instar larval antennal discs (larval), 8 h APF pupal antennal discs, 40 h APF antennae, and adult antennae. The color indicates the log-scaled relative expression values (base mean) of each gene at each stage from DESeq results. Reanalyzed from the previously published bulk data [29]. **(B)** *beat/side* genes are differentially expressed across ORN classes at single-cell resolution. Each column is an annotated ORN class, and each dot represents the expression level of the row gene in the given ORN class, summarizing from the single-cell (24 h APF, mid-pupal stage) or single-nucleus RNA-seq datasets (adult). The size of the dot denotes the fraction of “positive” cells in the given ORN class (log2(CPM + 1) > 0.5), and the color denotes the mean expression of the positive cells. Note that this single-nucleus RNA-seq dataset generally detects fewer transcripts than the single-cell RNA-seq dataset. Reanalyzed from the previously published data [64]. **(C)** Hierarchical genetic programs control ORN lineage and glomerular targeting. A simplified model illustrating the ORN-ORN kinship. ORNs are housed in sensillar subtypes within each sensillar type, representing different genetic lineages. ORNs mapped to the same class are defined as “within ORN class”, like ORN A1a and ORN A1a; ORNs of different classes but housed in the same sensillar subtype are defined as “within sensillar subtype”, like ORN A1a and ORN A1b; ORNs from different sensillar subtypes but belonging to the same sensillar type, are defined as “within sensillar type”, like ORN A1a and ORN A2a; and the furthest kinship is ORNs housed in distinct sensillar types, i.e., “between sensillar types”, like ORN A1a and ORN B1a. Schematic was created in BioRender. Duan, Q. (2026) https://BioRender.com/ds2elzi and https://BioRender.com/4rwwtaa. **(D)** *beat/side* expression in the antenna is regulated by sensillar type-specification factors *amos* and *atonal.* Volcano plot showing the differentially expressed genes in *amos* mutant antennae compared with wild-type antennae (left panel) or *atonal* mutant antennae compared with wild-type antennae (right panel). Significantly downregulated *beat/side* genes are colored in blue, and significantly upregulated *beat/side* genes are colored in red. Significance is determined by FDR < 0.05 from EdgeR results. The horizontal dashed line in each plot is FDR = 0.05. Two vertical dashed lines in each plot are log2FC = −0.5 and 0.5, respectively. Gray dots are all other genes detected. Reanalyzed from the previously published data [70,71]. **(E)** Pairwise correlation between ORNs at three stages reveals the *beat/side* combinatorial expression is correlated with the ORN kinship. Top row in each stage: boxplot showing the similarity measured by Spearman’s correlation between two cells from the indicated stage. The pairwise relation is categorized into four groups depending on the annotated ORN class and the corresponding sensillar type/subtype lineage. Bottom row in each stage: each dot is the mean similarity of the corresponding category in the top boxplot. The Spearman’s correlation was calculated based on the expression of all beat/side genes, all CSM-encoding genes, or the whole transcriptome (all genes). *P* values are from Mann–Whitney *U* tests without multiple comparison adjustments. Fig 2E is reproduced here for comparison. **(F)** *beat/side* combinatorial expression is more cell-population specific than pan-IgSF or pan-CSM genes across ORNs. Heatmap showing the Spearman’s correlation of combinatorial gene expression between the row cell and the column cell across three developmental stages (24 h APF, mid-pupal, and adult). The correlation was computed based on the combinatorial expression of *beat-side* genes, IgSF-encoding genes, or CSM-encoding genes. Cells are hierarchically clustered, as shown in the phylogeny tree in each heatmap. Correlation is shown as a spectrum from blue (−1) to red (1). The diagonal is the similarity of each cell to itself, and thus the correlation always equals 1. Analyses in (E) and (F) are derived from the same datasets used in (B). The data underlying this figure can be found in S3 Data.
(TIF)

S4 Fig Example single confocal sections showing the GAL4-labeled glomeruli across ORNs or PNs. Related to Figs 2 and 3. **(A)** Schematic of olfactory glomeruli in each layer of the antennal lobe, showing the relative position and stereotyped morphology of each glomerulus. The antennal lobe planes are organized from the posterior side to the anterior side. **(B–E)** Example single confocal section of the ORN or PN expression map of the indicated gene. The glomerular identity is assigned based on the Ncad staining compared with the template antennal lobe in (A). The glomeruli where the given gene is not expressed are highlighted in solid lines. V, DP1l, and DA4m glomeruli, shown in white line in (D), appear not to be labeled by GH146-FLP, serving as a negative control. **(F)** An example single confocal section showing a brain labeled by PN-specific *side-II* expression. Note that the *side-II* signal is present in VA2 and VA6 glomeruli of the right antennal lobe but absent in the left lobe.
(TIF)

S5 Fig Replicate the analyses in Fig 2 with the binned expression matrix of ORNs. Related to Fig 2. **(A)** Hierarchical clustering of *beat/side* expression across ORN classes based on the GAL4 labeling data. ORN lineages/sensillar types are color-coded. TSB, thin and small basiconics; LB, large basiconics; AT, antennal trichoids; AC, antennal coeloconics; PB, palp basiconics; SA, sacculus; AR, arista; UNK, unknown. For each gene, the expression in every glomerulus is binned into four relative expression levels: no expression (0), low (1), medium (2), or high expression (3). But for three genes, *beat-Vc*, *beat-Ic*, *beat-VI*, as all labeled glomeruli show significantly lower fluorescence intensity compared with the typical expression levels of glomeruli positive for other genes, no labeled glomerulus for these three genes was assigned to the “high” expression category, and instead classified only as medium or low expression. **(B)** Similarity (Pearson’s correlation) of *beat/side* expression between ORN class pairs based on the non-binary expression matrix of (A) in three relation categories. **(C)** Similarity (Pearson’s correlation) of *beat/side* expression between ORN class pairs based on the expression matrix of (A), with their identity labels shuffled. The data underlying this figure can be found in S1 and S4 Data.
(TIF)

S6 Fig Characterization of the expression of several *beat/side MiMIC* drivers in the mid pupal stage. Related to Figs 2 and 3. **(A)** ORN expression map of four *beat/side* genes in the developing brain. Single confocal sections are organized from the posterior side to the anterior side, with positive glomeruli highlighted by dashed lines. For *side*, which is expressed broadly across glomeruli, the outline of the whole antennal lobe is shown in dashed lines. **(B)** PN expression map of *side-IV* in the developing brain. Single confocal sections are organized from the posterior side to the anterior side, with positive glomeruli highlighted by dashed lines. **(C)** Comparison of the glomerular map of *beat/side* expression in mid-pupal and adult brains. ✓, detectable/expressed; ✗, undetectable/not expressed. * Full list of ORN glomeruli expressing *beat-IIb* in the adult but not mid-pupal brains: VP1m, VP2, VP3, VP4, DM1, DC4, DL1, DP1l, VC5, VM1, VL2a, DL2d, DL2v, VC3, VC1, VC2, VM4, DL4, DC3, DM5, VA1d, VA1v, VM5d, DA3, DA4m.
(TIF)

S7 Fig Characterization of *beat/side* expression in the published datasets of bulk and single-cell RNA-seq of PNs. Related to Fig 3. **(A)** *beat/side* expression in bulk PNs over development. Point plots showing the *beat/side* expression in the sorted bulk PNs from 36 h APF and the adult stage. The shape of each point denotes the biological replicate. The expression value is log2(CPM + 1). Reanalyzed from the previously published data [78]. **(B)** *beat/side* genes are differentially expressed across PN classes at single-cell resolution. Each column is an annotated PN class, and each dot represents the expression level of the row gene in the given PN class, summarizing from the single-cell RNA-seq datasets. The size of the dot denotes the fraction of “positive” cells in the given PN class (log2(CPM + 1) > 0.5), and the color denotes the mean expression of the positive cells. APL, anterior paired lateral neurons. vPN, ventral PN. APL and vPN clusters were not used for the correlation analysis in (D). Reanalyzed from the previously published data [79]. **(C)** Sequential genetic programs control PN lineage and glomerular targeting. We only used PNs mapped to adPN and lPN lineage for correlation analysis. PNs from these two lineages are excitatory and project to a single glomerulus, whereas the third lineage, vPN neurons, which were removed for downstream analysis, are inhibitory GABAergic and can project to multiple glomeruli [120,121]. In the adPN lineage, one of the two post-mitotic neurons survives and develops into a PN. In the lPN lineage, both post-mitotic neurons survive, one becoming a PN and the other becoming a local interneuron [81,84]. Schematic was created in BioRender. Duan, Q. (2026) https://BioRender.com/9ixpuo3. **(D)** Pairwise correlation between PNs at four stages reveals the *beat/side* combinatorial expression is correlated with the PN kinship. Top row in each stage: boxplot showing the similarity measured by Spearman’s correlation between two cells from the indicated stage. The pairwise relation is categorized into three groups depending on the annotated PN class identity and the corresponding lineage identity. Bottom row in each stage: each dot is the mean similarity of the corresponding category in the top boxplot. The Spearman’s correlation was calculated based on the expression of all *beat/side* genes, all cell surface molecule (CSM)-encoding genes, or the whole transcriptome (all genes). *P* values are from Mann–Whitney *U* tests without multiple comparison adjustments. Fig 3E is reproduced here for comparison. **(E)** *beat/side* combinatorial expression is more cell-population specific than pan-IgSF or pan-CSM genes across PNs. Heatmap showing the Spearman’s correlation of combinatorial gene expression between the row cell and the column cell across four developmental stages (0 h, 24 h APF, mid-pupal, and adult). The correlation was computed based on the combinatorial expression of *beat-side* genes, IgSF-encoding genes, or cell surface molecule-encoding genes. Correlation is shown as a spectrum from blue (−1) to red (1). Cells are hierarchically clustered, as shown in the phylogeny tree in each heatmap. The diagonal is the similarity of each cell to itself, and thus the correlation always equals 1. Analyses in (D) and (E) are derived from the same datasets used in (B). The data underlying this figure can be found in S3 Data.
(TIF)

S8 Fig Replicate the analyses in Fig 3 with the binned expression matrix of PNs. Related to Fig 3. **(A)** Hierarchical clustering of *beat/side* expression across PN classes based on the GAL4 labeling data. Uniglomerular PN lineages are color-coded. For each gene, the expression in every glomerulus is binned into four relative expression levels: no expression (0), low (1), medium (2), or high expression (3). Of note, we frequently observed labeling of vPN-like neurites in the VA1v and DA1 glomeruli, with *beat-V*I showing particularly strong vPN-associated signals. Because VA1v and DA1, in addition to a few other glomeruli, are innervated by projection neurons derived from vPN and uniglomerular adPN/lPN lineages, the presence of strong vPN labeling makes it difficult to unambiguously assess the expression status of the corresponding adPN/lPN-derived PNs (see S4 Data for PN lineage details). Therefore, signals observed in these glomeruli should be interpreted with caution, as they may predominantly reflect expression in vPNs rather than adPN/lPN-derived PNs. **(B)** Similarity (Pearson’s correlation) of *beat/side* expression between PN class pairs based on the expression matrix of (A) in two relation categories. **(C)** Similarity (Pearson’s correlation) of *beat/side* expression between PN class pairs based on the expression matrix of (A), with their identity labels shuffled. **(D)** Pearson’s correlation matrix based on the *beat/side* expression between each PN class pair in adPN lineage. PN classes are ordered according to the known birth sequence. **(E)** Pearson’s correlation matrix based on the *beat/side* expression between each PN class pair in lPN lineage. PN classes are ordered according to the known birth sequence. The data underlying this figure can be found in S1 and S4 Data.
(TIF)

S9 Fig Characterization of the in vivo Beats/Side protein expression. Related to Figs 2 and 3. **(A–F)** Example brain images showing the expression of the indicated Beat/Side protein, revealed by the MiMIC-based knock-in GFP tags. Images of *MiMIC-T2A-GAL4* driver-labeled brains are also shown for comparison. Scale bar (50 μm) in (A) applies to all rectangular brain images in (A) to (G). Scale bar (25 μm) in (B) applies to all square brain images in (B), (F), and (E). Yellow arrows in (A) denote axons of ventral PNs. Yellow arrows in (B) denote ventral PN axons, ORN axonal commissures, and the DM1 glomerulus, respectively, in *beat-IV*^*MI05715-GFP*^ brains (from top to bottom). DM1 glomerulus, negative for *beat-IV* in ORNs, is also highlighted by dashed circles in other panels. Yellow arrows and dashed circles in (C) denote the DM1 glomerulus, which is negative for *beat-Va* in either ORN or PN. Yellow arrows in (D) denote two apparent brain structures that are consistently labeled by in vivo tagged Side proteins and *MiMIC* GAL4-driven membrane marker. Yellow arrows in (E) denote the V glomerulus that is labeled by *side-V-*expressing ORNs. V glomerulus is also highlighted by dashed circles in other panels. Yellow arrows in (F) denote ORN axonal commissures in the upper panels and DM1 and DC4 glomeruli in the lower panels showing the single confocal section of *side-VI*^*MI01052-GFP*^ brains. DM1 (negative for *side-VI* in ORNs) and DC4 (positive for *side-VI* in ORNs) glomeruli are also highlighted by dashed circles in other panels. **(G)** Four representative fly brains carrying *MiMIC*-based GFP knock-in alleles, which do not rescue the loss-of-function phenotype and are therefore maintained as heterozygotes. The GFP signal is predominantly restricted to cell bodies. Upper right: AlphaFold-predicted structure of Beat-VI protein and the position where the *MiMIC*-based GFP tag is inserted. As the GFP tag is inserted inside the first Ig domain of Beat-VI, it very likely misfolds the protein and triggers ER retention. Lower left: AlphaFold-predicted structure of Side-V protein and the position where the *MiMIC*-based GFP tag is inserted. The GFP tag is inserted between two independently folded domains of Side-V (Ig5 and the fibronectin domain), which doesn’t seem to interfere with the function and localization of the Side-V protein.
(TIF)

S10 Fig Perturbation of *beats/sides* in ORNs leads to little to no defect in glomerular organization. Related to Fig 4. **(A)** Representative antennal lobes of controls and knockdowns of *beat/side* genes. A split phenotype of the DM3 glomerulus is highlighted with a solid circle. **(B)** Quantification of the percentage of lobes showing glomerular defects of VA1v and DM3 glomeruli in each genetic condition. The number of antennal lobes examined is shown in each bar. *P* values were determined by comparison to control groups. * *p* < 0.05. n.s., not significant. **(C)** Quantification of the percentage of lobes showing glomerular defects of VA1v glomerulus of *beat-IIIa/b/c* perturbation with additional *RNAi* transgenic background controls. The number of antennal lobes examined is shown in each bar. n.s., not significant. **(D)** Summary of expression of each gene in ORNs regarding to VA1v and DM3, respectively. * Knockdown of *beat-IV* or *side-V* with this *peb-GAL4* driver caused lethality. ^ Knockdown of *beat-IIa* was preliminarily tested for DM3 phenotype, but all nine brains examined appeared normal. **(E)** Representative antennal lobes showing the split DM3 glomerulus in the conditional knockout of *beat-IIIc* in ORNs, consistent with the *beat-IIIc RNAi* perturbation. **(F)** Representative antennal lobes showing that simultaneous knockout of *beat-IIa* and *beat-IIb* in ORNs didn’t cause a gross targeting phenotype of V glomerulus. A single confocal section of *side-IV MiMIC* GAL4-driven GFP expression in the antennal lobe is also shown to indicate that *side-IV* is likely to be expressed in PNs innervating the V glomerulus, comparable to the positive glomeruli DM1 and DM4 (Figs 4A, 4C, and S6B). **(G)** Representative antennal lobes showing that the ectopic expression of three *beats* didn’t produce an apparent glomerular targeting defect. These three *beat* genes, *beat-Ib*, *beat-Vc*, and *beat-VI*, are either barely expressed in ORNs or expressed only in a few ORN classes. The data underlying this figure can be found in S1 Data.
(TIF)

S11 Fig The effects of *beat/side* knockdown on synaptic density in ORNs. Related to Fig 4. **(A)** Synaptic density is quantified by the ratio of the average fluorescence intensity of RFP and GFP in the region of interest, i.e., the antennal lobe. Synaptic active zone marker, short BRP protein.mStraw, and membrane marker, mCD8.GFP, are translated from the single transcript but localized in different subcellular regions (axon terminals vs. general neurites). **(B)** Representative antennal lobes showing the effects of blocking neuronal activity of ORNs on gross glomerular synaptic density, visualized by the membrane marker GFP and presynaptic marker RFP. Scale bar representing 25 µm also applies to each panel in (D). **(C)** Quantification of (B). Each dot is a single antennal lobe. *N* = 43 and 13 antennal lobes, respectively. *P* value from the unpaired *t* test between groups is shown. **(D)** Representative antennal lobes showing the effects of knockdown of *beats/sides* on gross glomerular synaptic density, visualized by the membrane marker GFP and presynaptic marker RFP. Knockdown of *beat-IV* or *side-V* with this *peb-GAL4* driver caused lethality, and thus the data were missing. There is little to no expression of *beat-Ib/c*, *beat-Va/b/c*, or *beat-VI* across ORNs; *beat-IIIa/b/c*, though expressed broadly, are orphans. These genes were not tested for their loss-of-function effects on synaptic density. **(E)** Quantification of (D). Each dot is a single antennal lobe. *N* = 30, 14, 7, 26, 24, 27, 24, 20, 29, 20, 27, 32 antennal lobes, from left to right. Only *P* values that are significant or near the edge from multiple comparisons post-ordinary-one-way ANOVA are shown. The dashed horizontal line indicates the mean values in the *ctrl RNAi* group. Groups of *ctrl RNAi* and *side RNAi* are the same samples as in Fig 4J. **(F)** Volcano plot showing the differentially expressed genes in *Or47b* mutant antennae (top) and *Or67d* mutant antennae (bottom) compared with wild-type antennae. Significantly downregulated *beat/side* genes are colored in blue, and significantly upregulated *beat/side* genes are colored in red. Significance is determined by FDR < 0.05 from DESeq2 results. The horizontal dashed line in each plot is FDR = 0.05. Two vertical dashed lines in each plot are log2FC = −0.5 and 0.5, respectively. Gray dots are all other genes detected. Reanalyzed from previously published data [90]. The data underlying this figure can be found in S1 Data.
(TIF)

S12 Fig Additional analyses of *beat/sides* expression in mosquitoes and ants. Related to Fig 5. **(A)** AlphaFold-predicted structures of example Beat and Side orthologs in fruit flies *Drosophila* melanogaster (DROME), yellow fever mosquitoes *Aedes aegypti* (AEDAE), and clonal raider ants *Ooceraea biroi* (OOCBI). **(B)** Expression of *beats/sides* in the adult maxillary palp-housed ORN clusters of yellow mosquitoes based on the previously published single-cell RNA-seq data [91]. **(C)** Bulk RNA-seq showing the expression of *beats/sides* in developing antennal tissues of clonal raider ants, spanning from day 0 after pupal formation to the end of the pupal stage (day 14), reanalyzed from the data previously published [105]. **(D)** Linear regression analyses showing the high correlation of expression breadth in ORNs of *beat/side* orthologs between insect species. Our GAL4 labeling data were used and the finding in Fig 5G still holds true. The expression breadth of paralogs in each clade (i.e., *beat-Ia/b/c*, *beat-IIa/b*, etc) is averaged. **(E)** Heatmap showing the expression of *beats/sides* and *OR* genes in all ORN clusters of clonal raider ants that exhibit the ladder expression pattern of *OR* genes from 5′ end to 3′ end along a tandem, based on the single-cell RNA-seq data from [92]. *OR* genes are ordered from 5′ end to 3′ end in tandem in each cluster. Reanalyzed from the previously published data [92]. The data underlying this figure can be found in S1 Data.
(TIF)

## Abbreviations

AC: antennal coeloconic
APF: after puparium formation
APL: anterior paired lateral
cPCDH: clustered protocadherins
CSMs: cell-surface molecules
GR: gustatory receptor
IgSF: Immunoglobulin Super Family
IR: ionotropic receptor
LB: large basiconic
OR: olfactory receptor
ORNs: olfactory receptor neurons
PNs: projection neurons
ROI: regions of interest
SA: sacculus
