# Supplementary material for: Beat- and Side-family cell-surface molecules are expressed combinatorially in the partner neurons of the olfactory circuit in Drosophila
Source: PLoS Biol. 2026 Aug 27;24(8):e3003955. doi: 10.1371/journal.pbio.3003955 (PMC13521578; doi:10.1371/journal.pbio.3003955)
Supplement: Supplementary file 2 [file pbio.3003955.s020.pdf]

Reviewer #1:

In this manuscript PBIOLOGY-D-25-03410_R1 titled "Deciphering the combinatorial expression and genetic regulatory mechanisms of Beats and Sides in the olfactory circuits of Drosophila", the authors used MiMIC and previously published RNA-seq data to analyze the expression profile of beats/sides in ORNs, PNs and their synapses in AL glomeruli, and found a pattern of combinatorial expression. Functional analysis of one pair - Beat-IIa and Side-IV - suggested that they are not required for neurite targeting. Further knockdown suggested the role of some side genes in synaptic strength. Their homologs in mosquitoes and ants may have similar expression profiles.

The authors presented a substantial body of descriptive analysis using powerful genetic and imaging tools in Drosophila, complemented by functional and evolutionary investigations. Overall, the study provided valuable insights into the expression and function of adhesion molecules in neural development. As listed below, some key weaknesses remain — particularly regarding the depth of mechanistic validation. Addressing these issues will be essential for publication.

A major suggestion is to do more functional analyses by mutating/silencing other beat/side genes and generate at least double knockout/knockdown. In addition, for RNAi that do not show strong phenotypes, measure target gene expression level to evaluate RNAi knockdown efficiency.

We thank this reviewer's acknowledgement on the value and impact of our work. The primary motivation for this work is to provide a high-quality, high-resolution map of the expression patterns of beat/side genes in the olfactory circuit. The dataset generated in this study will serve as a valuable resource for developing new hypotheses, enabling more specific mechanistic investigations, particularly the combinatorial perturbation of multiple beat/side family members in specific ORN or PN types. As an initial step to interrogate the functions of these genes in ORN-PN circuit wiring, we screened a panel of these genes using a pan-ORN driver. We also utilized our expression atlas to focus on a specific pair, Beat-IIa and Side-IV, in matched ORN-PN pairs. We intended to follow up on positive hits with more detailed analyses and validation, including whole-animal mutant or MARCM studies. However, despite multiple RNAi and gRNA lines we screened for many of the genes, we did not observe an obvious wiring phenotype to pursue.

Taking reviewers' suggestions, we added additional functional investigation data to the revised paper:

(1) For our initial screening on glomerular targeting (V, VA1v, DM3 glomeruli), we used two different approaches to perturb each gene in ORNs, either by two independent RNAi lines or one RNAi line and one gRNA line, which induces somatic knockout. This screen revealed mild defects in the DM3 glomerulus, due to loss of *beat-IIIc*, and in the VA1v glomerulus, due to loss of *beat-IIb*.

(2) We also tried simultaneous knockout of two co-expressed genes, *beat-IIa* and *beat-IIb*, in ORNs using tissue-specific CRISPR and examined the corresponding glomerular targeting (the V glomerulus), though we didn't observe any obvious defect in that glomerulus.

(3) We tried over-expression of three sparsely or minimally expressed genes, *beat-Ib*, *beat-Vc*, *beat-VI*, respectively, in ORNs and examined glomerular targeting (V, VA1v, DM3 glomeruli). Unfortunately, these perturbations didn't give rise to any apparent wiring defects.

(4) For the Beat-IIa/Side-IV pair in DM1 and VA2 glomeruli, the RNAi reagents we used were also used with reported phenotypes in other published studies in the Drosophila visual system.

While these observations apply to the limited glomerular types we examined, it is entirely possible that stronger or more specific defects may emerge in other ORN/PN types with different combinations of *beat/side* perturbations. Indeed, our expression dataset suggests several intriguing candidate combinatorial manipulations of multiple *beat/side* genes in specific glomeruli. However, systematically testing these possibilities is technically demanding, which requires significant time to generate new transgenic reagents and extensive genetic crosses to assemble complex perturbation genotypes. We feel like this is beyond the scope of this paper.

Instead, we followed the reviewers' suggestions and further strengthened the expression and comparative analyses in this manuscript, which we believe provide a valuable resource and conceptual framework for future mechanistic investigations.

Please see our point-to-point responses for details in our revision.

Line-by-Line Comments and Suggestions

Line 76: "ORNs are thus defined by their OR expression and glomerular target identity, comprising ~60 classes (Task et al., 2022)." This is not accurate as 60 classes include 1/3 of IR-expressing neurons and projected glomeruli. There are likely 42 OR classes.

We thank the reviewer for pointing this out and we have now reworded this sentence, and it now reads as:

"Each ORN typically expresses only a single identity-defining chemosensory receptor gene — such as an olfactory receptor (OR), ionotropic receptor (IR), or gustatory receptor (GR) gene — or, in some cases, a unique combination of up to three receptor genes (Barish and Volkan, 2015). The cell bodies of ORNs expressing the same chemosensory receptor genes, thus of the same class, are dispersed across the antenna. Yet, their axons converge onto a single, uniquely positioned class-specific glomerulus in the antennal lobe, where they synapse with the second-order projection neurons (PNs) (Barish and Volkan, 2015; Hong and Luo, 2014). ORNs are thus defined by their chemosensory receptor expression and glomerular target identity, comprising ~60 classes (Task et al., 2022)."

Line 63-71 in the change-tracked version.

Line 160-164: Is there any antibody staining available for comparison with the GAL4 lines? While not strictly required, such data would help validate the expression patterns and ensure consistency.

We added several lines of evidence to support the reliability of GAL4 lines.

(1) For five genes, we happen to have gene trap lines from two independent MiMIC insertion sites. These lines showed consistent labeling patterns in the brain, which validates our method. We added a new supplementary figure (**new Figure S1**) to present these results.

(2) We also tested several MiMIC-based protein trap lines in which a GFP tag is inserted into MiMIC sites within the coding introns, allowing direct visualization of those Beat/Side proteins. We also found a comparable labeling pattern between MiMIC-GFP and MiMIC-GAL4. For example, Side-GFP and side-T2A-GAL4-driven membrane marker show similar brain-wide expression patterns (**Reviewer Figure 1-1B, C; new Figure S9D**). These data are added to a new supplementary figure (**new Figure S9**).

However, when we tested the only existing antibody for the Side protein, we found a discrepancy. A recent study (Thornton-Kolbe et al., 2025) reported that many IgSF genes, including *side*, are not expressed in Kenyon cells of the Mushroom Body (Reviewer Figure 1D). This is consistent with both our MiMIC-GAL4 and MiMIC-GFP data for *side*, where the signal is absent in the Mushroom Body lobes (**Reviewer Figure 1-1B, C**). But anti-Side antibody detects an apparent signal across the Mushroom Body (**Reviewer Figure 1-1A**), suggesting nonspecific staining of this antibody. We therefore didn't include the antibody staining data in our manuscript.

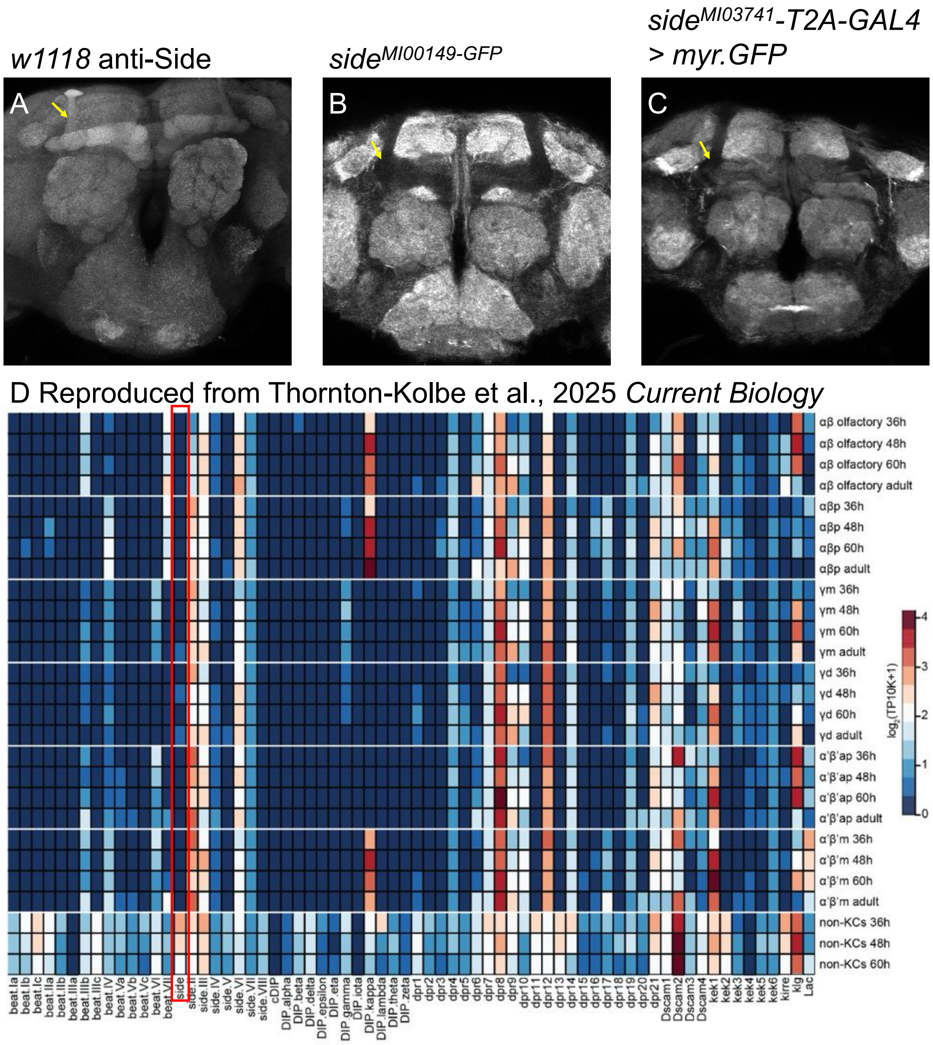

Reviewer Figure 1-1. (A) Anti-Side staining for a w1118 brain. (B) Anti-GFP staining for a *side*$^{MI00149\text{-}GFP}$ brain. (C) Anti-GFP staining for a *side-T2A-GAL4*-driven membrane-bound GFP brain. Yellow arrows in (A), (B), and (C) indicate the mushroom body lobes where Kenyon cells' neurites innervate. (D) Heatmap

showing the expression of IgSF genes in the Mushroom Body neurons throughout development. Note that the *side* gene is largely depleted in all Kenyon cells except the γ subtype (red rectangle).

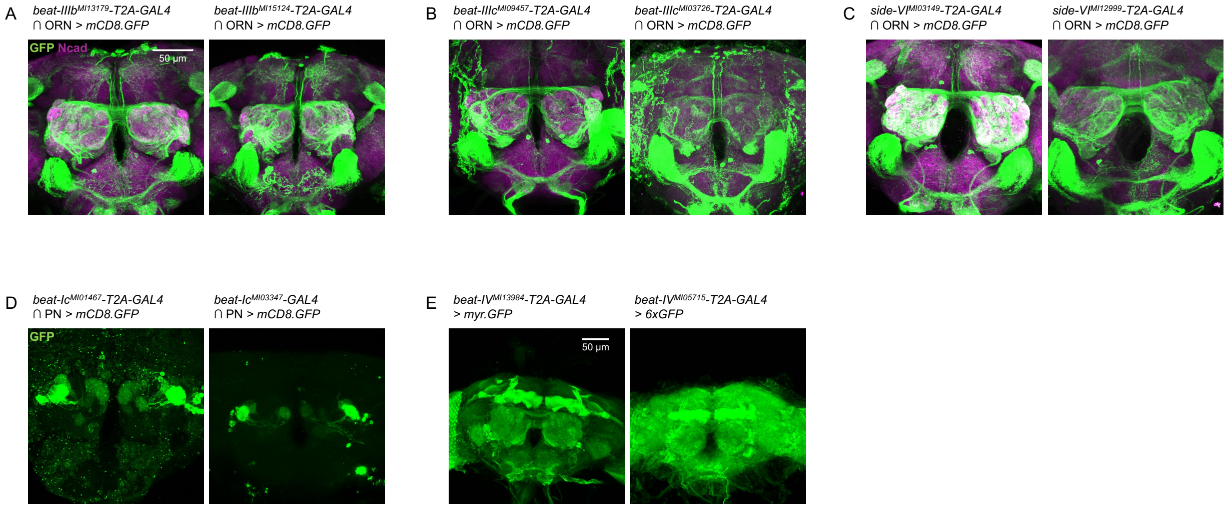

Reviewer Figure 1-2 (new Figure S1). Consistent expression patterns across different *MiMIC* driver lines for the same gene. Related to Figure 1. (A-E) Representative images showing brains labeled by two different *MiMIC*-based gene trap drivers targeting the same gene. Scale bar in (A) applies to images in (A) to (D). Insertion site for these additional *MiMIC* swap lines: *beat-IIIb*^{MI15124}: coding intron, targeting 2/2 isoforms; *beat-IIIc*^{MI09457}: coding intron, targeting 2/2 isoforms; *side-VI*^{MI12999}: coding intron, targeting 2/2 isoforms; *beat-Ic*^{MI01467}: coding intron, targeting 3/3 isoforms; *beat-IV*^{MI05715}: coding intron, targeting 2/2 isoforms.

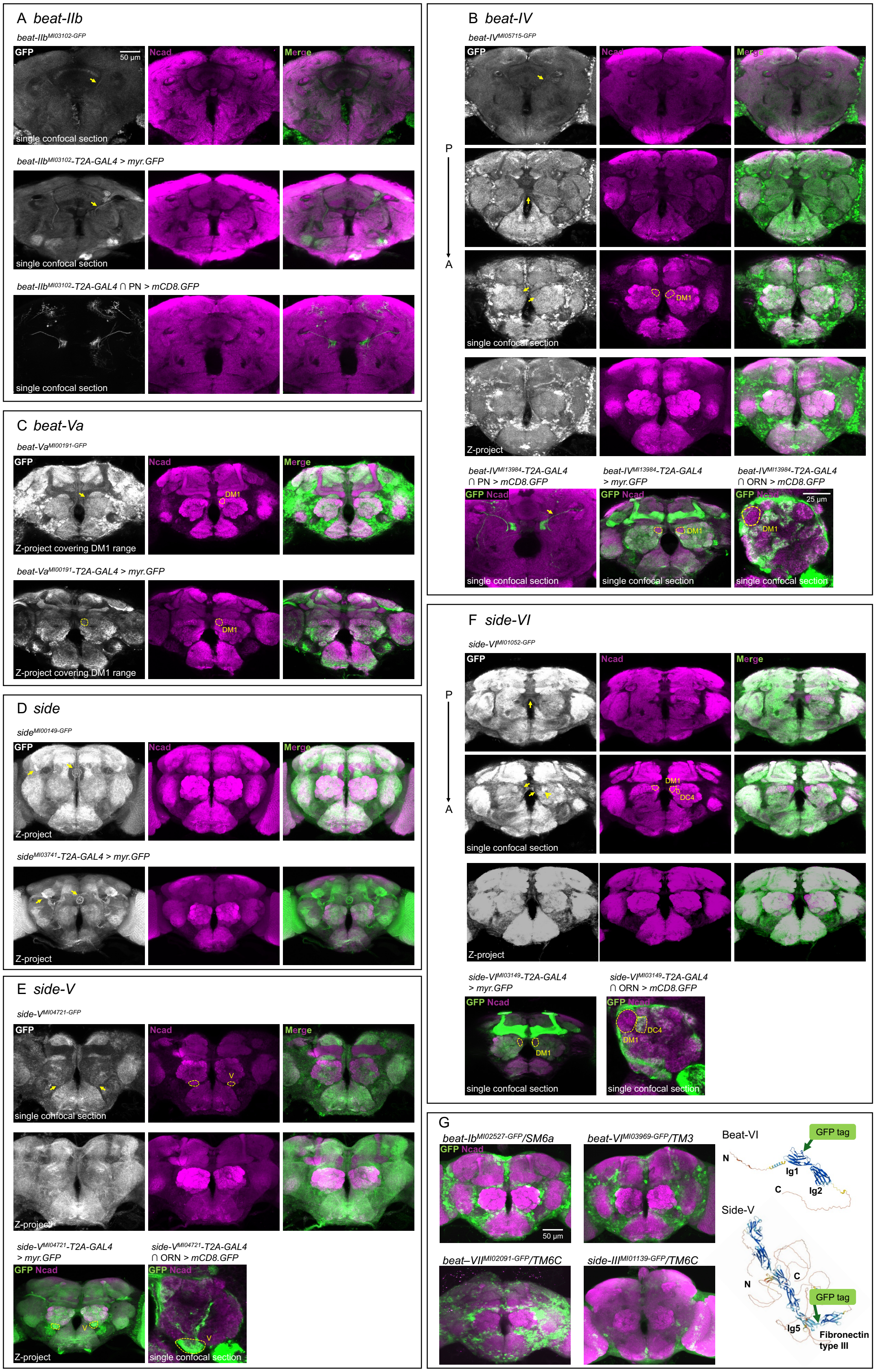
A beat-IIb
beat-IIbMI03102-GFP
GFP
50 µm
Ncad
Merge
single confocal section
beat-IIbMI03102-T2A-GAL4 > myr.GFP
single confocal section
beat-IIbMI03102-T2A-GAL4 ∩ PN > mCD8.GFP
single confocal section
B beat-IV
beat-IVMI05715-GFP
GFP
Ncad
Merge
P
A
DM1
single confocal section
Z-project
beat-IVMI13984-T2A-GAL4 ∩ PN > mCD8.GFP
beat-IVMI13984-T2A-GAL4 > myr.GFP
beat-IVMI13984-T2A-GAL4 ∩ ORN > mCD8.GFP
GFP Ncad
25 µm
DM1
single confocal section
C beat-Va
beat-VaMI00191-GFP
GFP
Ncad
Merge
DM1
Z-project covering DM1 range
beat-VaMI00191-T2A-GAL4 > myr.GFP
DM1
Z-project covering DM1 range
D side
sideMI00149-GFP
GFP
Ncad
Merge
Z-project
sideMI03741-T2A-GAL4 > myr.GFP
Z-project
E side-V
side-VMI04721-GFP
GFP
Ncad
Merge
V
single confocal section
Z-project
side-VMI04721-T2A-GAL4 > myr.GFP
side-VMI04721-T2A-GAL4 ∩ ORN > mCD8.GFP
GFP Ncad
V
Z-project
single confocal section
F side-VI
side-VIMI01052-GFP
GFP
Ncad
Merge
P
A
DM1
DC4
single confocal section
Z-project
side-VIMI03149-T2A-GAL4 > myr.GFP
side-VIMI03149-T2A-GAL4 ∩ ORN > mCD8.GFP
GFP Ncad
DM1
DC4
single confocal section
G
beat-IbMI02527-GFP/SM6a
beat-VIMI03969-GFP/TM3
GFP Ncad
50 µm
beat–VIIMI02091-GFP/TM6C
side-IIIMI01139-GFP/TM6C
Beat-VI
GFP tag
N
Ig1
C
Ig2
Side-V
C
N
GFP tag
Ig5
Fibronectin type III

Reviewer Figure 1-3 (new Figure S9). Characterization of the *in vivo* Beats/Side protein expression. Related to Figures 2, 3. (A-F) Example brain images showing the expression of the indicated Beat/Side protein, revealed by the MiMIC-based knock-in GFP tags. Images of *MiMIC-T2A-GAL4* driver-labeled brains are also shown for comparison. Scale bar (50 μm) in (A) applies to all rectangular brain images in (A) to (G). Scale bar (25 μm) in (B) applies to all square brain images in (B), (F), and (E). Yellow arrows in (A) denote axons of ventral PNs. Yellow arrows in (B) denote ventral PN axons, ORN axonal commissures, and the DM1 glomerulus, respectively, in *beat-IV*$^{MI05715\text{-}GFP}$ brains (from top to bottom). DM1 glomerulus, negative for *beat-IV* in ORNs, is also highlighted by dashed circles in other panels. Yellow arrows and dashed circles in (C) denote the DM1 glomerulus, which is negative for *beat-Va* in either ORN or PN. Yellow arrows in (D) denote two apparent brain structures that are consistently labeled by *in vivo* tagged Side proteins and *MiMIC* GAL4-driven membrane marker. Yellow arrows in (E) denote the V glomerulus that is labeled by *side-V*-expressing ORNs. V glomerulus is also highlighted by dashed circles in other panels. Yellow arrows in (F) denote ORN axonal commissures in the upper panels and DM1 and DC4 glomeruli in the lower panels showing the single confocal section of *side-VI*$^{MI01052\text{-}GFP}$ brains. DM1 (negative for *side-VI* in ORNs) and DC4 (positive for *side-VI* in ORNs) glomeruli are also highlighted by dashed circles in other panels. **(G)** Four representative fly brains carrying *MiMIC*-based GFP knock-in alleles, which do not rescue the loss-of-function phenotype and are therefore maintained as heterozygotes. The GFP signal is predominantly restricted to cell bodies. Upper right: AlphaFold-predicted structure of Beat-VI protein and the position where the *MiMIC*-based GFP tag is inserted. As the GFP tag is inserted inside the first Ig domain of Beat-VI, it very likely misfolds the protein and triggers ER retention. Lower left: AlphaFold-predicted structure of Side-V protein and the position where the *MiMIC*-based GFP tag is inserted. The GFP tag is inserted between two independently folded domains of Side-V (Ig5 and the fibronectin domain), which doesn't seem to interfere with the function and localization of the Side-V protein.

Line 194: "Consistent with bulk and single-cell RNA-seq" - if they were previous data, add citation.

This is now fixed. We refer to the **new Figure S3**, and its legend cites the source data.

Line 204-205: "More interestingly, ORN classes from the same lineage tend to be clustered based on the combinatorial beat/side expression (Figure 2B)." It is not clear, as even Ir and Or seem somewhat clustered.

It is indeed the case. As shown in **Figure 2B**, Ir-expressing glomeruli tend to be clustered together (see the row label for the tuning receptors expressed by each glomerulus) based on the collective expression of *beat/side* genes. This is not unexpected, as IRs are selectively expressed in AC-lineage-derived and Sacculus/Arista-housed ORNs. Therefore, the clustering of Ir glomeruli is coupled with the clustering of AC/SA/AR sensillar types.

Line 240-262: The correlation data are not strong. Suggest to genetically track lineage during development to show "two-step hierarchical mechanism", or at least discuss it.
Also, in this analysis, would be interesting to see how transcription factors turn on and off and their correlations with beat/side expression.

In this study, we found that ORNs within the same sensillar type have more similar *beat/side* combinatorial profiles, which holds true across all three different datasets: binary GAL4 labeling matrix, binned GAL4 labeling matrix, and the single-cell RNA-seq. We noticed that the effect size in the single-cell RNA-seq data is relatively small, likely reflecting the inherent technical noise associated with this dataset. However, the same correlation trend is more pronounced in our GAL4 labeling dataset, which is cleaner and more comprehensive. Together, these observations support the robustness of our conclusion.

We take the reviewer's suggestion to elaborate on the description and discussion of how lineage-defining transcription factor expression correlates with *beat/side* expression. This part now reads as:

"For example, both *beat-Ia* and *beat-IIa* are predominantly expressed in the Amos lineage-derived ORNs (Antennal Thin and Small Basiconic, TSB; Large Basiconics, LB; Antennal Tricoids, AT) and largely absent in the Atonal lineage-derived ORNs (Antennal Coeloconics, AC); these two genes are significantly downregulated in *amos* mutants (Figure 2B; Figure S3D, S5A). By contrast, *beat-Ib* and *side-IV* are expressed at higher levels in several AC sensillar types, despite lower-level expression in the Amos lineage, and thus show significant downregulation in *atonal* mutants (Figure S3D, S5A). A few genes are also upregulated in *amos* and *atonal* mutants (*side-VIII* in *amos* mutants, *side-II* in *atonal* mutants; Figure S3D). These *beat* and *side* genes are either transcriptionally upregulated or are now enriched in the mutants due to loss of ORNs and changes in ORN-specific transcript ratios. Together, these results suggest that the specification of *beat/side* expression is downstream of earlier ORN fate determination."

Line 228-238 in the change-tracked version.

Line 284-288: This is interesting and would merit further discussion. In Discussion (Line 531-549), the template - variation idea is expanded. Were there any similar studies on other adhesion molecules, which support this idea?

We thank the reviewer for acknowledging this highlight. We added a new paragraph in the **Discussion** to elaborate on the idea:

"The two-step model is conceptually supported by other highly diversified neuronal recognition systems, including clustered protocadherins (cPCDH) in mammals and Dscam in fruit flies. Both systems generate enormous molecular diversity through combinatorial paralog expression or extensive alternative splicing and were initially thought to rely largely on stochastic expression mechanisms. However, more recent studies suggest that lineage-dependent developmental programs can bias or propagate specific molecular identities. Neurons with closer developmental origin tend to share significantly more similar cPCDH or Dscam1 isoforms, which further drives homophilic binding-mediated repulsion to finely pattern the mouse neocortex and *Drosophila* medulla columns, respectively (Liu et al., 2020; Lv et al., 2022). Together, these findings support a hierarchical strategy in which lineage-dependent molecular templates are subsequently diversified to establish neuronal identity and circuit specificity."

Line 665-675 in the change-tracked version.

Line 377: Not clear why choose this pair. There seem other cases for "expressing in a few classes". Also, would the genes with expression in more classes possibly lead to stronger phenotypes?

To test the function of Beats/Sides in the olfactory circuit wiring, we started with a screening focused on the targeting and organization of three glomeruli (V, VA1v, DM3) by knocking down the *beat/side* genes expressed in ORNs innervating these or nearby glomeruli. We also tested the overexpression of a few *beat* genes and multiplexed perturbation of two co-expressed *beats*. However, we didn't find any striking glomerular phenotype. We therefore turned to additional Beat-Side and ORN-PN pairs. Compared with many other candidate interactions, beat-IIa is expressed in only a few ORN classes and side-IV is similarly restricted in PNs, making this pair experimentally tractable and easier to interpret. Also, these two interacting partners have been shown to instruct synaptic recognition in the Drosophila visual system. The RNAi reagents we used were also validated in previous studies (Jain et al., 2022; Osaka et al., 2024). We have revised the manuscript to clarify the rationale underlying our experimental design and candidate selection:

"To test whether *beat/side* plays any role in glomerular targeting or organization, we conducted a pan-ORN *peb-GAL4*-driven *UAS-RNAi* screening against *beat* and *side* genes exhibiting decent expression in specific ORNs and examined whether three glomeruli would show any defects as a readout. These include Gr21a ORNs, Or47b ORNs, and Or47a ORNs, targeting V, VA1v, and DM3 glomerulus, respectively (Figure S10A). We included perturbation of *side-VIII*, which is not expressed in any ORNs, as an additional negative control. For each gene perturbed, we used two independent approaches: either two different *RNAi* lines to knock down or one *RNAi* line and one gRNA line inducing a somatic knockout (Figure S10B). First, we noticed that V glomerulus appeared wild-type in all perturbations (Figure S10A). Second, this screen revealed local organizational defects in the VA1v glomerulus by knockdown of *beat-IIb, beat-IIIa/b/c,* and *side-III*. Among these, the VA1v phenotype was reproduced by two independent *RNAi* lines against *beat-IIb*, but the defects in *beat-IIIa/b/c* or *side-III* knockdowns could not be robustly observed in secondary perturbations (Figures S10B). Additional controls showed that the VA1v phenotypes of *beat-IIIa/b/c* knockdowns appeared to arise from the transgenic background that sensitizes the VA1v glomerular disorganization (Duan et al., 2023)(Figure S4C). Third, we observed a low-penetrance phenotype of split DM3 glomerulus, which appeared in both *RNAi* knockdown and gRNA-mediated ORN-specific knockout of *beat-IIIc* (Figure S10A, B, E). Overall, we identified two hits with mild glomerular phenotypes from this targeted screen: *beat-IIb* in Or47b ORNs and *beat-IIIc* in Or47a ORNs. However, Beat-IIb-interacting Side-IV is not expressed in postsynaptic PNs innervating the VA1v glomerulus or nearby regions. Coincidentally, *side-IV* is restrictedly expressed in Or47b and Or88a ORNs, which target a neighboring glomerulus (Figure 2A, B), raising the possibility for axon-axon contacts mediated by co-expressed Beat-IIb and Side-IV, which is interesting for future exploration. Additionally, Beat-IIIc is an orphan cell-surface protein with no characterized interacting partners (Figure 1A), which limits our further investigation of its role in glomerular organization. Moreover, we made a preliminary effort with multiplexing perturbation. We found that *beat-IIa* and *beat-IIb* are markedly co-expressed in the V glomerulus-innervating ORNs. Side-IV, which interacts with Beat-IIa and Beat-IIb, appears to be expressed in PNs targeting the V glomerulus as well (Figure S10F). We therefore used ORN-specific CRISPR to simultaneously knock out *beat-IIa* and *beat-IIb but* didn't find an overtly defective glomerular organization (Figure S10F). In addition, we also performed gain-of-function experiments using pan-ORN overexpression of *beat* genes that are sparsely expressed across ORNs, but did not observe any obvious mistargeting phenotypes (Figure S10G).

We then turned to other glomeruli with the matching expression of interacting Beats and Sides between partner ORN/PN classes. We found one straightforward case: presynaptic Beat-IIa and postsynaptic Side-IV. *beat-IIa* is expressed in just a few ORN classes, including Or42b ORNs targeting one posterior glomerulus DM1 and Or92a ORNs targeting one anterior glomerulus VA2 (Figure 4B). In parallel, *side-IV* expression is restricted in PNs, including PNs projecting to DM1 and VA2 glomeruli (Figure 4C). Next, we tested whether perturbing *beat-IIa* expression in ORNs or *side-IV* expression in PNs results in glomerular targeting defects, using the *RNAi* lines validated with reported phenotypes in previous studies (Jain et al., 2022; Osaka et al., 2024)."

Line 440-479 in the change-tracked version.

Line 376-389: This is just single knockdown/knockout, suggesting redundancy. Try to make at least double knockout. For example, knockout of beat-IIa and beat-IIb in ORN (both linked to side-IV in PN); knockout of side-IV and side in PN (both linked to beat-IIa in ORN).

We agree with the reviewer that functional redundancy is likely an important feature of the Beat/Side system. Motivated by this possibility, we performed an additional multiplex perturbation experiment by simultaneously knocking out *beat-IIa* and *beat-IIb* in ORNs using ORN-specific CRISPR. These two genes are markedly co-expressed in V glomerulus-innervating ORNs, and their interacting partner Side-IV is also expressed in the corresponding PNs. However, even this double perturbation did not produce any overt glomerular organization defect **(new Figure S10F)**. We have now incorporated these additional data and discussion into the revised manuscript:

"Moreover, we made a preliminary effort with multiplexing perturbation. We found that *beat-IIa* and *beat-IIb* are markedly co-expressed in the V glomerulus-innervating ORNs. Side-IV, which interacts with Beat-IIa and Beat-IIb, appears to be expressed in PNs targeting the V glomerulus as well (Figure S10F). We therefore used ORN-specific CRISPR to simultaneously knock out *beat-IIa* and *beat-IIb but* didn't find an overtly defective glomerular organization (Figure S10F)."

Line 463-467 in the change-tracked version.

We agree that these two genes may be important for glomerular targeting in other ORN/PN classes, and that testing additional co-expressed Beat/Side combinations in specific glomeruli would be valuable. However, a systematic exploration of numerous combinatorial perturbations is beyond the scope of the current study.

Line 376-438: For RNAi knockdown of some genes, which showed either no or mild phenotypes, it is important to check RNAi efficiency, e.g. using FISH or other methods. Otherwise, it is hard to interpret the results.

We agree that RNAi efficiency is an important consideration when interpreting negative or mild phenotypes. In this study, we attempted to mitigate this concern by using two independent perturbation strategies for each candidate gene whenever possible, including either **two independent RNAi lines** or **one RNAi line together with a gRNA-mediated somatic knockout** approach. In addition, for the Beat-IIa/Side-IV experiments, we specifically selected RNAi lines that had previously been validated and shown to produce phenotypes in published studies. We mentioned this in the text.

"Next, we tested whether perturbing *beat-IIa* expression in ORNs or *side-IV* expression in PNs results in glomerular targeting defects, **using the *RNAi* lines validated with reported phenotypes in previous studies** (Jain et al., 2022; Osaka et al., 2024)."

Line 477-479 in the change-tracked version.

We acknowledge that directly measuring knockdown efficiency using FISH or related methods would further strengthen the interpretation of individual perturbations. However, given the screening-based nature of this study and the large number of candidate genes examined, systematically validating the efficiency of all perturbations that produced negative results would be technically challenging and beyond the scope of the current work. Instead, our strategy was to prioritize positive hits for follow-up analyses.

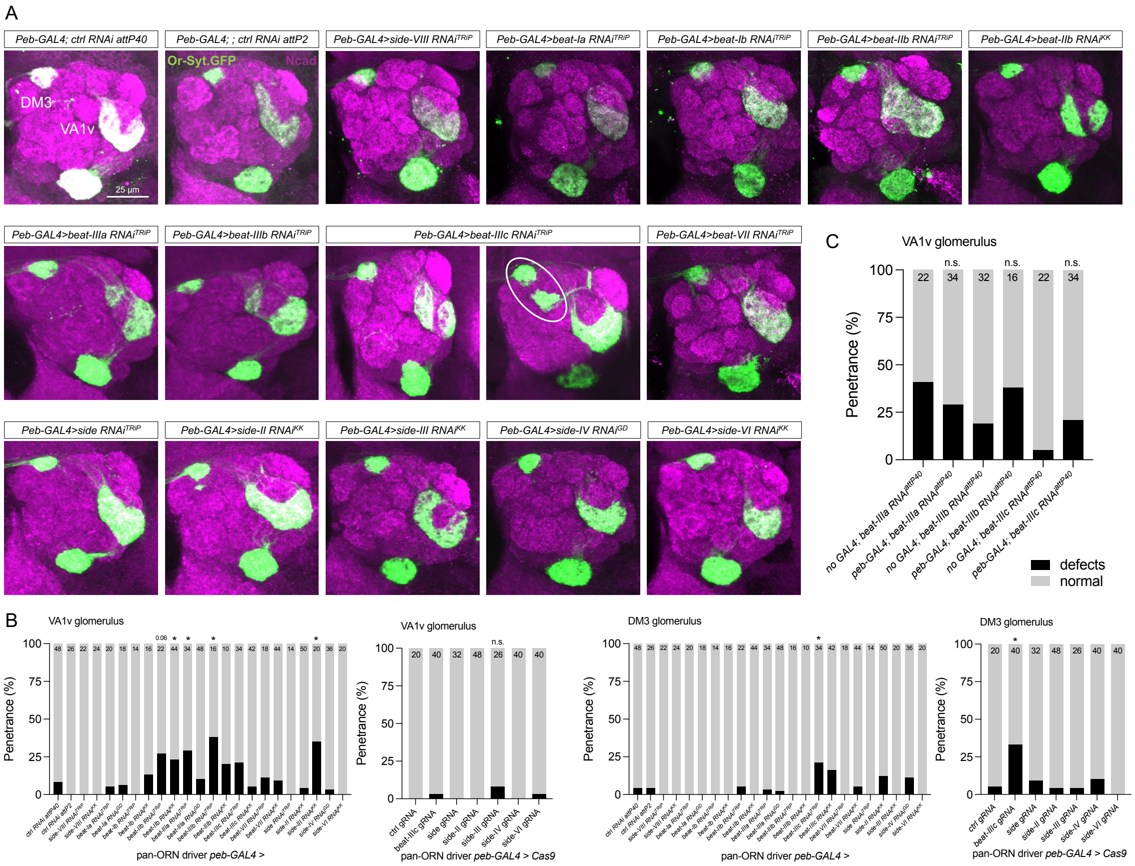

D

Expression pattern	Gene
Not expressed anywhere (negative control)	*side-VIII*
Not expressed in VA1v but expressed in neighboring glomeruli of VA1v	*beat-Ia*
Expressed in VA1v	*beat-Ib, beat-IIb, beat-IIIa, beat-IIIb, beat-IIIc, beat-IV*, beat-VII, side, side-II, side-III, side-IV, side-V*, side-VI*
Not expressed in VA1v or any nearby glomeruli (not tested)	*beat-Ic, beat-IIa^, beat-Va, beat-Vc, beat-VI*

Expression pattern	Gene
Not expressed anywhere (negative control)	*side-VIII*
Not expressed in DM3 but expressed in neighboring glomeruli of DM3	*beat-IIa^, beat-IIb, beat-Vc, side, side-III, side-V**
Expressed in DM3	*beat-Ia, beat-IIIa, beat-IIIb, beat-IIIc, beat-IV*, beat-VII, side-II, side-VI*
Not expressed in DM3 or any nearby glomeruli	*beat-Ib, beat-Ic, beat-Va, beat-VI, side-IV*

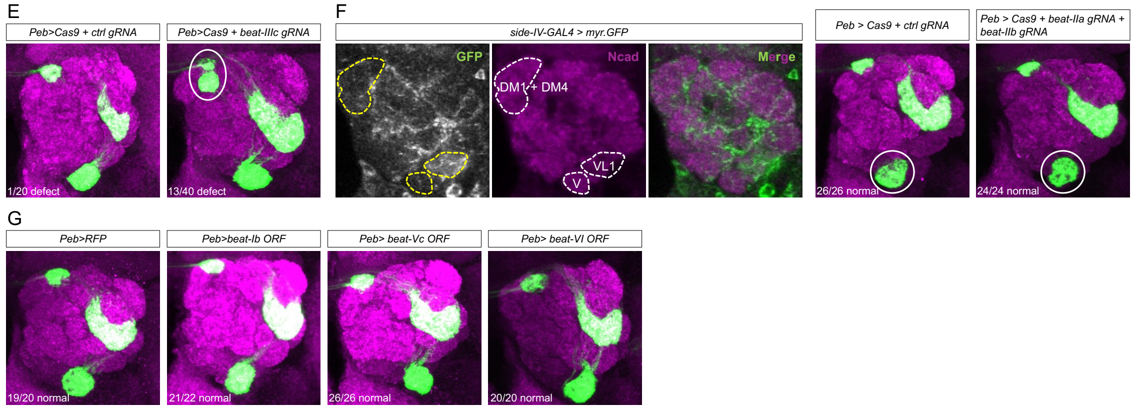

Reviewer Figure 1-4 (new Figure S10). Perturbation of *beats/sides* in ORNs leads to little to no defect in glomerular organization. Related to Figure 4. (A) Representative antennal lobes of controls and knockdowns of *beat/side* genes. A split phenotype of the DM3 glomerulus is highlighted with a solid circle. **(B)** Quantification of the percentage of lobes showing glomerular defects of VA1v and DM3 glomeruli in each genetic condition. The number of antennal lobes examined is shown in each bar. P

values were determined by comparison to control groups. *, p<0.05. n.s., not significant. **(C)** Quantification of the percentage of lobes showing glomerular defects of VA1v glomerulus of *beat-IIIa/b/c* perturbation with additional *RNAi* transgenic background controls. The number of antennal lobes examined is shown in each bar. n.s., not significant. **(D)** Summary of expression of each gene regarding to VA1v and DM3, respectively. * Knockdown of *beat-IV* or *side-V* with this *peb-GAL4* driver caused lethality. ^ Knockdown of *beat-IIa* was preliminarily tested for DM3 phenotype, but all nine brains examined appeared normal. **(E)** Representative antennal lobes showing the split DM3 glomerulus in the conditional knockout of *beat-IIIc* in ORNs, consistent with the *beat-IIIc RNAi* perturbation. **(F)** Representative antennal lobes showing that simultaneous knockout of *beat-IIa* and *beat-IIb* in ORNs didn't cause a gross targeting phenotype of V glomerulus. A single confocal section of *side-IV MiMIC* GAL4-driven GFP expression in the antennal lobe is also shown to indicate that *side-IV* is likely to be expressed in PNs innervating the V glomerulus, comparable to the positive glomeruli DM1 and DM4 (Figure 4A, C; Figure S6B). **(G)** Representative antennal lobes showing that the ectopic expression of three *beats* didn't produce an apparent glomerular targeting defect. These three *beat* genes, *beat-Ib*, *beat-Vc*, and *beat-VI*, are either barely expressed in ORNs or expressed only in a few ORN classes.

Line 498-499: This sentence is confusing: "… the ORNs in each cluster are transcriptionally heterogeneous, containing multiple ORN classes defined by the functional expression of a singleton OR protein". Does it mean ORNs that express OR genes of a cluster (array)? Or ORNs in a cell cluster in a sensillum? The meaning of "transcriptionally heterogeneous" is not clear here. Singleton refers to OR gene that is not in a gene cluster (array) - It does not fit the context.

We thank the reviewer for pointing this out and have reworded it more precisely:

"Interestingly, recent single-cell RNA-seq reports from clonal raider ant ORNs revealed an unusual mode of OR gene regulation in which multiple OR transcripts can be detected within individual ORNs. In detail, ORNs that share a more similar transcriptome, and thus are clustered together, usually express *ORs* located in a tandem genomic array. **In each ORN, transcription is initiated from a selected upstream start site and can extend through multiple downstream OR genes in the array, while only the most upstream OR mRNA in the transcript is translated into a functional protein (Brahma et al., 2023). As a result, ORNs within the same transcriptomic cluster can express different functional OR proteins despite sharing highly similar overall gene expression profiles. Thus, the ORNs are transcriptionally similar at the global transcriptome level but heterogeneous in their functional OR identity.**"

Line 601-611 in the change-tracked version.

Line 502-509: It is nice to do this comparison. But in Figure S6E, there are many variations even within the same ORN classes (the ones expressing the same most upstream OR genes), so not sure whether comparison between ORN classes is conclusive.

We thank the reviewer for pointing this out and we agree that there is variability within ORN classes. A more comprehensive investigation of this question using additional single-cell RNA-seq datasets from different ant species and insect taxa would be valuable, but is beyond the scope of the current study. We therefore toned down our interpretation in the revised manuscript:

"To zoom in on ORN classes within each single-cell RNA-seq cluster, we plotted *beat/side* expression and the *ORs* of the tandem expressed in the cluster of each single cell as heatmaps (Figure S12E). We found that each ORN class, defined by the expression of the most upstream *OR*, exhibits class-specific expression of *beat/sides*. For example, in cluster 3, the ORN class defined by the functional expression of Or5-9E109 doesn't express the ant *side-IVb,* whereas other ORN classes functionally expressing the adjacent Ors, Or5-9E108, Or5-9E107, and Or5-9E110 appear to be positive for this gene (Figure S12E). Similarly, ORN classes defined by the functional expression of Or5-9E151 or Or5-9E150 in cluster 14 don't express the ant *beat-VII,* whereas many other ORN classes in this cluster do (Figure S12E). **While these differences are not absolute, these observations raise the possibility that,** upon OR tandem choice, additional developmental mechanisms may couple *OR* transcription initiation and *beat/side* expression specification in the clonal raider ant."

Line 613-624 in the change-tracked version.

Before Figure 1D, provide a structure of Drosophila brain indicating the locations of the antennal lobe, central complex, mushroom body...

We updated **Figure 1** with a new panel depicting several key anatomical regions of the fly brain.

Figure S2B, Nice dotplots. Describe and explain why there is a significant change of expression profiles from mid-pupal to adult stage in S2B? Do they switch their adhesion molecules to maintain synapses?

This is now the **new Figure S3B**.

The difference likely arises because the adult dataset was generated using single-nucleus RNA-seq (due to technical difficulties in isolating intact high-quality adult ORNs from hardened cuticle), whereas the mid-pupal data are from single-cell RNA-seq. Importantly, to benchmark these methods, the original study performed both single-nucleus and single-cell RNA-seq on 24h-APF ORNs and conducted a direct comparison. They reported that, in general, the single-nucleus approach detects fewer transcripts than single-cell per cell/nucleus. Consistent with this technical limitation, most discrepancies between the mid-pupal and adult datasets involve the reduced expression levels in adult ORNs. In contrast, a subset of genes showed higher expression in adults (*beat-Ia*, *beat-VII*), consistent with the bulk RNA-seq data (Figure S3A), suggesting that at least some of the observed differences likely reflect genuine biological changes. Overall, direct quantitative comparison between the mid-pupal and adult datasets should be interpreted with caution, as differences may partly reflect technical distinctions between the two methods rather than biological changes alone. Since this technical difference is not central to our study, we chose, for simplicity and consistency, to use the "single-cell RNA-seq" term throughout the manuscript. We explicitly noted this difference in the legend of the supplementary figure:

"**(B)** *beat/side* genes are differentially expressed across ORN classes at single-cell resolution. Each column is an annotated ORN class, and each dot represents the expression level of the row gene in the given ORN class, summarizing from the single-cell (24h APF, mid-pupal stage) or single-nucleus RNA-seq datasets (adult). The size of the dot denotes the fraction of "positive" cells in the given ORN class (log2(CPM+1) > 0.5), and the color denotes the mean expression of the positive cells. **Note that this**

single-nucleus RNA-seq dataset generally detects fewer transcripts than the single-cell RNA-seq dataset. Reanalyzed from the previously published data (McLaughlin et al., 2021).”

Line 999-1001 in the change-tracked version.

Figure S3B, similar changes observed from mid-pupal to adult as in ORNs, but also appears to have more extensive expression than ORNs. Why? Is this a general case and what is the biological significance: "broadly and less specifically located postsynaptic surface locks are matched by sparsely and more specifically distributed presynaptic surface keys" (Line 318)?

This is now presented as the **new Figure S7B**.

In contrast to the ORN datasets, both the mid-pupal and adult PN datasets were generated using the same single-cell RNA-seq approach and are therefore more directly comparable technically. However, the adult dataset captured fewer PN classes than the mid-pupal dataset, likely reflecting differences in sampling or recovery efficiency. Nevertheless, for clusters confidently assigned to the same single PN class identity, *beat/side* expression patterns are largely stable between these two developmental stages (new Figure S7B). This developmental stability is also consistent with the expression pattern of many other cell adhesion molecules reported in the original study (Figure 9—figure supplement 2 therein).

It is also true that PNs generally express a broader repertoire of *beat/side* genes than ORNs. Interestingly, a recent study showed that Kenyon cells of the mushroom body, which are postsynaptic to PNs, similarly express much fewer IgSF molecules (Reviewer Figure 1D), resembling ORNs that are presynaptic to PNs. Together, these observations suggest that PNs, as the middle layer of the olfactory circuit, may require more diverse cell-surface repertoires to interact with numerous upstream and downstream partners, whereas their synaptic partner populations employ comparatively sparser and more selective molecular codes. However, because we have not systematically examined additional neural circuits, we do not wish to overgeneralize this observation as a universal organizational principle and therefore only discuss the new finding in Kenyon Cell briefly in the revised manuscript:

“Notably, a recent work found that in Kenyon cells of the mushroom body that receive inputs from olfactory PNs, many IgSF genes, including most *beats*, are not expressed, whereas many *sides* are (Thornton-Kolbe et al., 2025). This raises the possibility that some Beat proteins in PNs might also act on axon terminals to interact with postsynaptic Kenyon cells, while some Side family proteins are also required on the PN dendrites during ORN-PN matching.”

Line 705-710 in the change-tracked version.

Figure S2C, the antenna with sensilla of varied types is nice, but too small - enlarge it substantially.

We have made it larger and confirmed that it is sufficiently clear when readers open the full-resolution version we uploaded and zoom in **(new Figure S3C)**.

Figure 4A, the connection lines below plot are hard to track. Revise it with some differences (color or thickness or dash) between adjacent lines.

We found that using different colors would make the figure even busier. Alternatively, we added texts of the PN interacting partners underneath the ORN ones **(new Figure 4A)**.

Reviewer #2:

In this study, Duan and colleagues address a fundamental question in neuroscience which is how specific targeting is achieved. They focus on the Beats and Sides, a network of interacting IgSF proteins in Drosophila that were implicated in neuromuscular junction and visual system development. However, unlike the similar network of Dpr/DIPs, Beat/Sides remain poorly characterized in terms of biochemical properties, expression pattern and, most importantly, functional relevance within developing neural circuits. Here, the authors mainly focus on expression - either using published RNA-seq datasets or via a nice collection of beat- and side-Gal4s, and use the ORN-PN targeting within the antennal lobe as a model. I think this is an extremely useful and appropriate model as approximately 50 types of ORNs synapse on 50 PNs on a stereotypic and well structured antennal lobe where their synapses form distinct glomeruli (although additional LNs are completely dismissed from their analysis). This relatively small yet complex structure offered the authors the ability to perform a near-comprehensive analysis of the expression of all beat/side members - 14 and 8 parlors, respectively - within the 50 types of ORNs and 50 types of matching PNs. Their analysis of the existing transcriptional datasets is impressive, and their work provides reliable and important data, especially given our very limited knowledge of Beat/Side expression in developing circuits. However, this work is based on three pillars - (1) expression analysis, (2) mechanism, (3) evolution - unfortunately, each one of these is underdeveloped and together their current status lacks coherence, is unconvincing and is therefore not ripe for publication in a high impact journal. In fact, I see potential for three independent stories, and I encourage the authors to delve deep in each one of these directions. Below are just a few directions that could improve each one of the pillars - but if you decided to keep all three together then your major task is to tell a coherent, clear, and well supported story.

We thank the reviewer for recognizing the significance and potential of this work, and we appreciate the insightful perspective that the expression, mechanistic, and evolutionary aspects could each potentially develop into independent studies. Our primary goal in this study was to establish a high-quality, high-resolution atlas of *beat/side* expression in the olfactory circuit, which we believe provides an important foundational resource for the field. Given the limited prior knowledge of Beat/Side expression patterns in the olfactory circuit, we felt that systematically defining their cellular and glomerular specificity across ORNs and PNs was a necessary first step toward future functional dissection.

As an initial effort to investigate the functional roles of Beats/Sides in ORN–PN wiring, we performed a targeted perturbation screen using both RNAi- and gRNA-based approaches, followed by additional multiplex perturbation and gain-of-function experiments (though

preliminary). While we identified several mild phenotypes, we did not observe strong or penetrant wiring defects that would justify a substantially deeper mechanistic study within the timeframe of this revision.

We agree with the reviewer that more extensive combinatorial manipulations across additional ORN/PN classes could reveal stronger functions. Indeed, our expression dataset suggests several intriguing candidate combinatorial manipulations of multiple *beat/side* genes in specific glomeruli. However, systematically pursuing these possibilities would require the generation of new transgenic reagents and complex genetic crosses to assemble the perturbation genotypes and is therefore beyond the scope of the current work.

Following this reviewer's many constructive suggestions, we therefore chose to substantially strengthen the expression component of the manuscript, which we believe now forms a more coherent central narrative. We reanalyzed our original imaging datasets to generate the binned expression matrices of *beats/sides*, including four categorical expression levels, in ORNs and PNs, in addition to the binary matrices. We found that our major conclusions hold true with the new datasets. We examined the glomerular expression data for several key genes in the developing brain. We also provided the data showing the *in vivo* protein expression of several Beats/Sides using the MiMIC-GFP protein trap reagents. We believe these additions considerably reinforce the expression "pillar" of the study and provide an important conceptual framework for future mechanistic investigations.

Importantly, we also view the evolutionary analyses as closely connected to the central story rather than as an independent direction. The conservation of *beat/side* expression patterns across divergent insect species suggests that the regulation of these genes is likely under evolutionary selection pressure, which in turn implies functional relevance in ORN development and/or function. In this sense, the evolutionary conservation provides indirect evidence supporting functional roles of Beats/Sides, complementing the relatively mild or negative phenotypes observed in our current perturbation experiments. Together with the lineage-correlated combinatorial organization observed in Drosophila, these findings support the idea that developmental programs coordinately regulate duplicated cell-surface recognition molecules during neural circuit assembly.

Please see our point-by-point responses below for details of the revisions.

Expression pillar:

While this is the most developed part of the paper, there are still directions to improve the existing data and major directions to delve deeper.

1) the binarization of the expression data to - expressed and not expressed - was obviously an efficient way to simplify it but in my opinion also flattened it in an exaggerated manner. For example, Side-V is expressed broadly in all ORNs - but clearly, there are ORNs that express Side-V in a higher level than others - is this not important? This is true for all the broadly expressed Gal4s. The reason I am worried by this extreme flattening of the data is that it makes the hierarchical clustering unconvincing (BTW - I could not find the details on how this was done) - if half of the beats and sides are broadly expressed, three - five almost absent, the the clustering is done based on four Gal4 lines plus very little input of those all or none Gal4 lines. At the very least adding a few expression levels (even just three, in addition to not expressed) would, in my opinion, open new avenues to analysis.

We thank the reviewer for this insightful suggestion and agree that binary classification may oversimplify the richness of the expression patterns. We now provide semi-quantitative expression matrices binning expression in each glomerulus for each gene into four discrete levels: no, low, medium, and high. We observed similar clustering results in both ORNs and PNs and found that the lineage-correlated conclusion remains robust (**new Figure S5, S8**). We added these results to the manuscript accordingly (Line 211-214, 261-262, 346-350, 381-385 in the change-tracked version).

We would also like to clarify that we largely agree with the reviewer's interpretation that hierarchical clustering is primarily driven by a subset of differentially expressed *beat/side* genes rather than by uniformly broad expression across the family. In fact, this observation motivated an important part of our proposed model. As discussed in the manuscript, some *beat/side* genes exhibit strong lineage-biased expression patterns and may function as lineage-specific "templates," whereas other genes appear to be sporadically activated or repressed during later ORN fate specification steps. For example, *beat-Ia* is largely excluded from AC and sacculus ORNs, while *beat-IIa* is preferentially enriched in large basiconic (LB) ORNs. We therefore proposed a two-step hierarchical model in which broad lineage-dependent programs establish a core combinatorial template that is subsequently diversified among related ORN classes. In this framework, the clustering structure emerging from a subset of lineage-biased genes is not merely a technical artifact of binarization, but rather reflects an underlying biological organization principle that we now discuss more explicitly in the revised manuscript (Line 268-279 in the change-tracked version).

We explained the details of the binned expression level analysis in the **Method** part:

"As we aimed to clearly determine whether each glomerulus is positive or negative for each gene, we didn't use the constant parameters to acquire the images for different *GAL4* driver lines. We adjusted the acquisition parameters in order to clearly reveal glomeruli with very low expression. After scanning each brain from the most posterior end to the most anterior end of the antennal lobe, we referred to the previously characterized glomerular map (Task et al., 2022) and manually determined the glomerular identity and the corresponding *beat/side* expression (Figure S4), assigning 0 to negative expression and 1 to positive expression. At least three brains were examined for each *GAL4* driver line. Notably, for the *GH146-FLP*-based labeling, we occasionally found inconsistent glomerular innervation patterns between individuals and even right and left lobes of the same brain (data not shown). This is likely due to the incomplete excision of the *STOP* cassette in a subset of PNs. As each glomerulus could be innervated by as few as one PN, it is possible that some glomeruli are not labeled in an antennal lobe. We thus determined these glomeruli to be positive if we observed the GFP signal for that glomerulus in two or more antennal lobes.

Given that inconsistent imaging parameters were used between different driver lines, quantifying and comparing the gene expression level of a particular glomerulus across different genes based on the fluorescence intensity is not appropriate. However, for each individual gene (i.e., the same *GAL4* driver line) consistent imaging settings were kept across brains, allowing relative comparison of expression levels between glomeruli for that gene. We therefore also adopted a semi-quantitative scoring approach in which the expression level of each gene in each glomerulus was binned into four discrete categories — no, low, medium, and high expression — based on *GAL4*-labeled fluorescence intensity. We assigned values of 0, 1, 2, and 3, respectively. This approach was chosen for two reasons. First, fluorescence intensity measurements can be influenced by variability in staining quality, brain mounting, tissue morphology, and segmentation accuracy, making precise grayscale-based quantification less robust across samples. Second, we also found the presence of occasional outlier glomeruli with exceptionally strong fluorescence signals (e.g., V glomerulus ORNs for *beat-IIIc* and *side-III*, VL1 glomerulus ORNs for *side-IV*, Figure 2A). Normalizing expression values to the brightest glomerulus for each gene would compress the dynamic range of the remaining glomeruli, causing many moderately or robustly labeled glomeruli to be represented by disproportionately small normalized values. As a result, continuous intensity-based normalization could distort the relative expression spectrum and reduce the

interpretability of biologically meaningful differences between glomeruli. Binning expression into discrete levels instead preserves overall and reproducible relative differences in expression while reducing sensitivity to technical variability and extreme outliers. Notably, for three genes with regard to the ORN expression map, *beat-Vc*, *beat-Ic*, *beat-VI*, even the most strongly labeled glomeruli exhibit substantially lower fluorescence intensity compared with the typical expression levels observed for other genes; therefore, to preserve the relative expression scale across the dataset, we did not assign any glomeruli for these genes to the "high" expression category and instead classified them only as medium or low expression. The binary expression matrix and the binned matrix were then input into the downstream hierarchical clustering analysis using the heatmap.2 function with the default settings in R (based on the pairwise Euclidean distance)."

Line 1327-1366 in the change-tracked version.

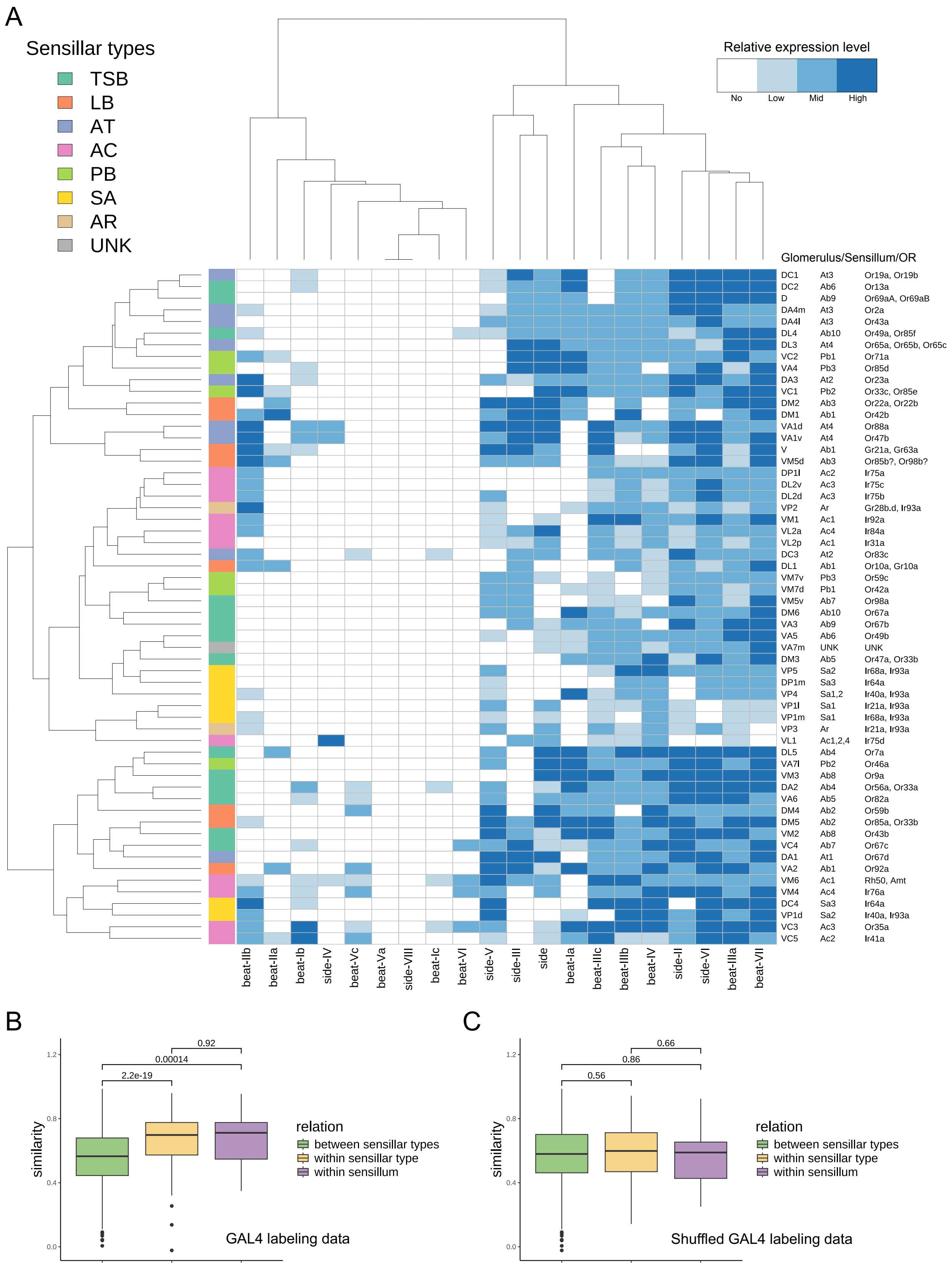
A
Sensillar types
TSB
LB
AT
AC
PB
SA
AR
UNK
Relative expression level
No
Low
Mid
High
Glomerulus/Sensillum/OR
DC1 At3 Or19a, Or19b
DC2 Ab6 Or13a
D Ab9 Or69aA, Or69aB
DA4m At3 Or2a
DA4l At3 Or43a
DL4 Ab10 Or49a, Or85f
DL3 At4 Or65a, Or65b, Or65c
VC2 Pb1 Or71a
VA4 Pb3 Or85d
DA3 At2 Or23a
VC1 Pb2 Or33c, Or85e
DM2 Ab3 Or22a, Or22b
DM1 Ab1 Or42b
VA1d At4 Or88a
VA1v At4 Or47b
V Ab1 Gr21a, Gr63a
VM5d Ab3 Or85b?, Or98b?
DP1l Ac2 Ir75a
DL2v Ac3 Ir75c
DL2d Ac3 Ir75b
VP2 Ar Gr28b.d, Ir93a
VM1 Ac1 Ir92a
VL2a Ac4 Ir84a
VL2p Ac1 Ir31a
DC3 At2 Or83c
DL1 Ab1 Or10a, Gr10a
VM7v Pb3 Or59c
VM7d Pb1 Or42a
VM5v Ab7 Or98a
DM6 Ab10 Or67a
VA3 Ab9 Or67b
VA5 Ab6 Or49b
VA7m UNK UNK
DM3 Ab5 Or47a, Or33b
VP5 Sa2 Ir68a, Ir93a
DP1m Sa3 Ir64a
VP4 Sa1,2 Ir40a, Ir93a
VP1l Sa1 Ir21a, Ir93a
VP1m Sa1 Ir68a, Ir93a
VP3 Ar Ir21a, Ir93a
VL1 Ac1,2,4 Ir75d
DL5 Ab4 Or7a
VA7l Pb2 Or46a
VM3 Ab8 Or9a
DA2 Ab4 Or56a, Or33a
VA6 Ab5 Or82a
DM4 Ab2 Or59b
DM5 Ab2 Or85a, Or33b
VM2 Ab8 Or43b
VC4 Ab7 Or67c
DA1 At1 Or67d
VA2 Ab1 Or92a
VM6 Ac1 Rh50, Amt
VM4 Ac4 Ir76a
DC4 Sa3 Ir64a
VP1d Sa2 Ir40a, Ir93a
VC3 Ac3 Or35a
VC5 Ac2 Ir41a
beat-IIb
beat-IIa
beat-Ib
side-IV
beat-Vc
beat-Va
side-VIII
beat-Ic
beat-VI
side-V
side-III
side
beat-Ia
beat-IIIc
beat-IIIb
beat-IV
side-II
side-VI
beat-IIIa
beat-VII
B
similarity
1.2
0.8
0.4
0.0
0.92
0.00014
2.2e-19
relation
between sensillar types
within sensillar type
within sensillum
GAL4 labeling data
C
similarity
1.2
0.8
0.4
0.0
0.66
0.86
0.56
relation
between sensillar types
within sensillar type
within sensillum
Shuffled GAL4 labeling data

Reviewer Figure 2-1 (new Figure S5). Replicate the analyses in Figure 2 with the binned expression matrix of ORNs. Related to Figure 2. (A) Hierarchical clustering of *beat/side* expression across ORN classes based on the GAL4 labeling data. ORN lineages/sensillar types are color-coded. TSB, thin and small basiconics; LB, large basiconics; AT, antennal trichoids; AC, antennal coeloconics; PB, palp basiconics; SA, sacculus; AR, arista; UNK, unknown. For each gene, the expression in every glomerulus is binned into four relative expression levels: no expression (0), low (1), medium (2), or high expression (3). But for three genes, *beat-Vc*, *beat-Ic*, *beat-VI*, as all labeled glomeruli show significantly lower fluorescence intensity compared with the typical expression levels of glomeruli positive for other genes, no labeled glomerulus for these three genes was assigned to the "high" expression category, and instead classified only as medium or low expression. **(B)** Similarity (Pearson's correlation) of *beat/side* expression between ORN class pairs based on the non-binary expression matrix of (A) in three relation categories. **(C)** Similarity (Pearson's correlation) of *beat/side* expression between ORN class pairs based on the expression matrix of (A), with their identity labels shuffled.

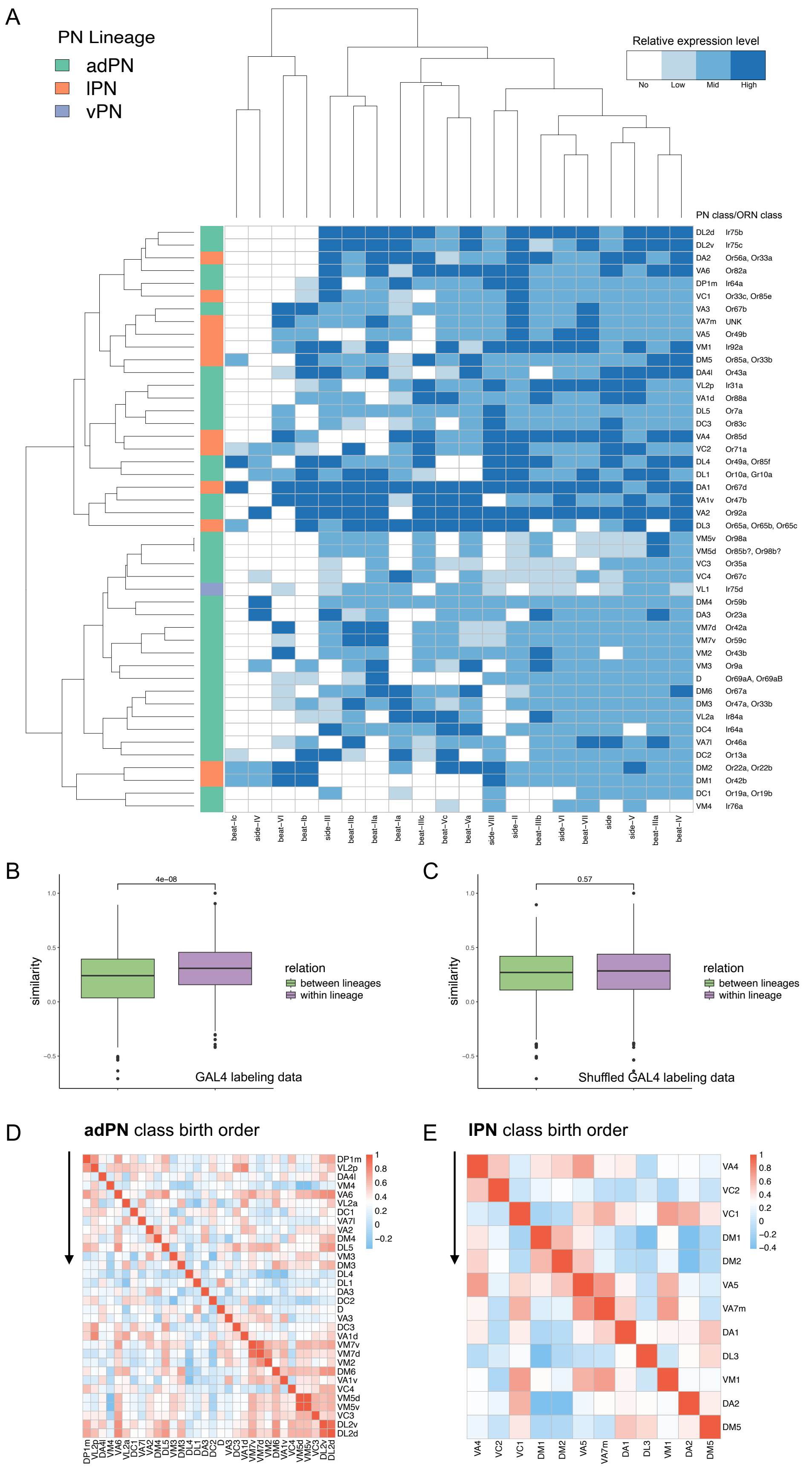
A
PN Lineage
adPN
lPN
vPN
Relative expression level
No
Low
Mid
High
PN class/ORN class
DL2d Ir75b
DL2v Ir75c
DA2 Or56a, Or33a
VA6 Or82a
DP1m Ir64a
VC1 Or33c, Or85e
VA3 Or67b
VA7m UNK
VA5 Or49b
VM1 Ir92a
DM5 Or85a, Or33b
DA4l Or43a
VL2p Ir31a
VA1d Or88a
DL5 Or7a
DC3 Or83c
VA4 Or85d
VC2 Or71a
DL4 Or49a, Or85f
DL1 Or10a, Gr10a
DA1 Or67d
VA1v Or47b
VA2 Or92a
DL3 Or65a, Or65b, Or65c
VM5v Or98a
VM5d Or85b?, Or98b?
VC3 Or35a
VC4 Or67c
VL1 Ir75d
DM4 Or59b
DA3 Or23a
VM7d Or42a
VM7v Or59c
VM2 Or43b
VM3 Or9a
D Or69aA, Or69aB
DM6 Or67a
DM3 Or47a, Or33b
VL2a Ir84a
DC4 Ir64a
VA7l Or46a
DC2 Or13a
DM2 Or22a, Or22b
DM1 Or42b
DC1 Or19a, Or19b
VM4 Ir76a
beat−Ic
side−IV
beat−VI
beat−Ib
side−III
beat−IIb
beat−IIa
beat−Ia
beat−IIIc
beat−Vc
beat−Va
side−VIII
side−II
beat−IIIb
side−VI
beat−VII
side
side−V
beat−IIIa
beat−IV
B
4e−08
similarity
relation
between lineages
within lineage
GAL4 labeling data
C
0.57
similarity
relation
between lineages
within lineage
Shuffled GAL4 labeling data
D
adPN class birth order
DP1m
VL2p
DA4l
VM4
VA6
VL2a
DC1
VA7l
VA2
DM4
DL5
VM3
DM3
DL4
DL1
DA3
DC2
D
VA3
DC3
VA1d
VM7v
VM7d
VM2
DM6
VA1v
VC4
VM5d
VM5v
VC3
DL2v
DL2d
E
lPN class birth order
VA4
VC2
VC1
DM1
DM2
VA5
VA7m
DA1
DL3
VM1
DA2
DM5

Reviewer Figure 2-2 (new Figure S8). Replicate the analyses in Figure 3 with the binned expression matrix of PNs. Related to Figure 3. (A) Hierarchical clustering of *beat/side* expression across PN classes based on the GAL4 labeling data. Uniglomerular PN lineages are color-coded. For each gene, the expression in every glomerulus is binned into four relative expression levels: no expression (0), low (1), medium (2), or high expression (3). Of note, we frequently observed labeling of vPN-like neurites in the VA1v and DA1 glomeruli, with *beat-V*I showing particularly strong vPN-associated signals. Because VA1v and DA1, in addition to a few other glomeruli, are innervated by projection neurons derived from vPN and uniglomerular adPN/lPN lineages, the presence of strong vPN labeling makes it difficult to unambiguously assess the expression status of the corresponding adPN/lPN-derived PNs (See Table S1 for PN lineage details). Therefore, signals observed in these glomeruli should be interpreted with caution, as they may predominantly reflect expression in vPNs rather than adPN/lPN-derived PNs. **(B)** Similarity (Pearson's correlation) of *beat/side* expression between PN class pairs based on the expression matrix of (A) in two relation categories. **(C)** Similarity (Pearson's correlation) of *beat/side* expression between PN class pairs based on the expression matrix of (A), with their identity labels shuffled. **(D)** Pearson's correlation matrix based on the *beat/side* expression between each PN class pair in adPN lineage. PN classes are ordered according to the known birth sequence. **(E)** Pearson's correlation matrix based on the *beat/side* expression between each PN class pair in lPN lineage. PN classes are ordered according to the known birth sequence.

2) some data is difficult for us to follow - for example, Beat-VII in Fig. 2 appears to be all over the place yet they claim it is excluded from two specific glomeruli - they need to provide support as to how they reached this conclusion (for example show the single confocal slices zoomed in on these glomeruli, or however they determined this). Same for Side-II in Figure 3 - how did they conclude it's excluded from seven specific glomeruli?

We thank the reviewer for pointing out this. In the revision, we provide a section-by-section image with glomeruli identity indicated for these two genes in ORNs and PNs, respectively (**new Figure S4**). We mentioned in the manuscript:

"As the glomerular identity in the fruit fly antennal lobe is well mapped, we manually examined the single confocal section and assigned the expression status of each gene to every glomerulus (Figure S4A-C). … Using the fully mapped antennal lobe template, we examined individual confocal imaging planes to assign the expression status of each gene to every glomerulus (Figure S4A, D, E)."

Line 192-194, 343-345 in the change-tracked version.

Additionally, for other genes, we can share the original imaging datasets, including individual optical sections, upon request or through an appropriate public repository capable of hosting large imaging files.

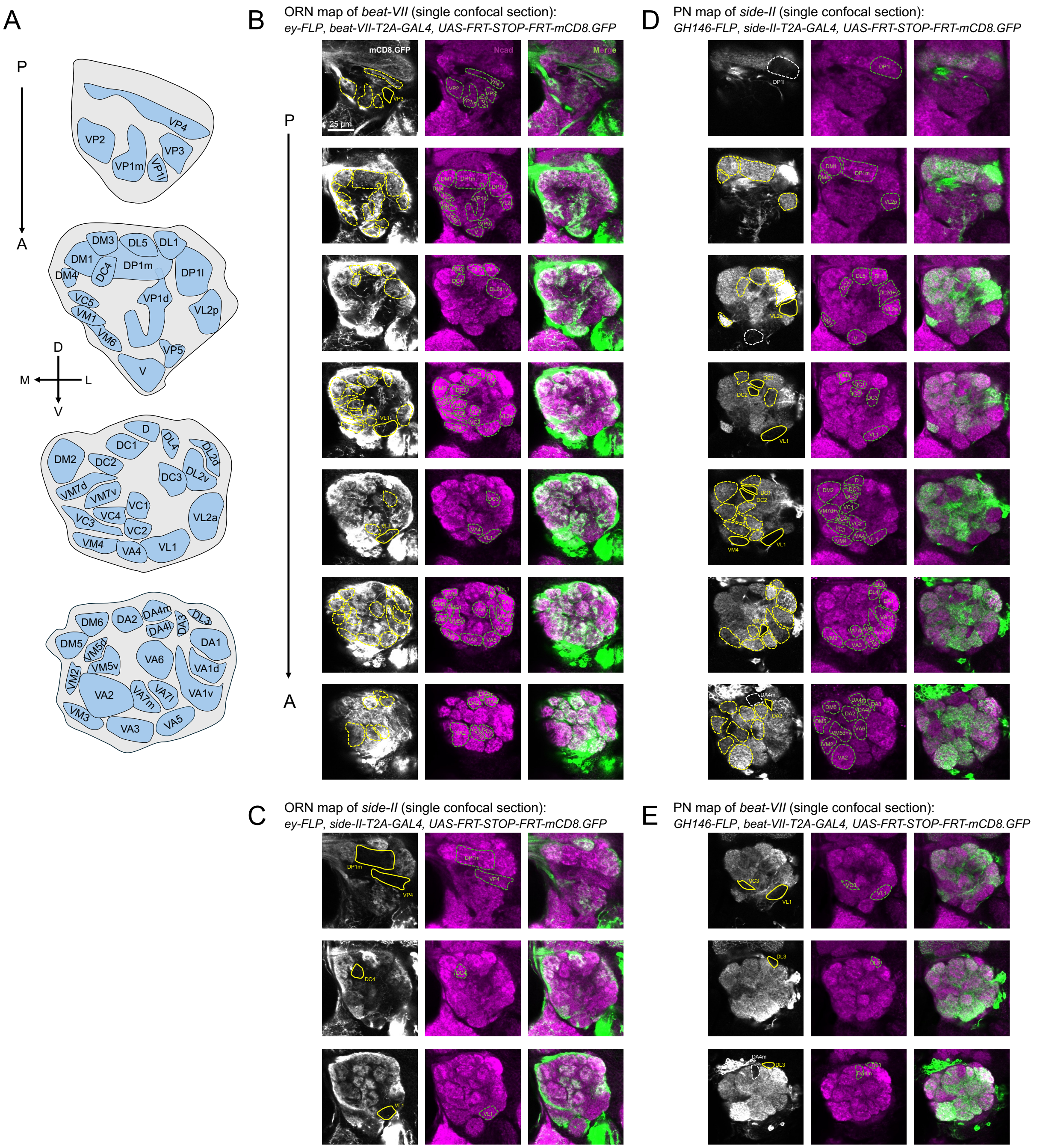

Reviewer Figure 2-3 (new Figure S4). Example single confocal sections showing the GAL4-labeled glomeruli across ORNs or PNs. Related to Figures 2, 3. (A) Schematic of olfactory glomeruli in each layer of the antennal lobe, showing the relative position and stereotyped morphology of each glomerulus. The antennal lobe planes are organized from the posterior side to the anterior side. **(B-E)** Example single confocal section of the ORN or PN expression map of the indicated gene. The glomerular identity is assigned based on the Ncad staining compared with the template antennal lobe in (A). The glomeruli where the given gene is not expressed are highlighted in solid lines. V, DP1l, and DA4m glomeruli, shown in white line in (D), appear not to be labeled by GH146-FLP, serving as a negative control.

Delve deeper:

3) this is a work on development, yet the expression patterns are recorded in adult. I understand that dissecting pupal brains is more tedious but much more informative.

We selected several genes to examine their ORN/PN expression in development (~ 36-48h APF). We added texts referring to the **new Figure S6** in the **Results** section:

"We also examined the ORN expression map of several genes in the mid pupal brains (~ 36-48h APF), including two genes with a restricted glomerular distribution (*beat-IIa*, *beat-IIb*), one gene with a widespread glomerular expression (*side*), and one gene that is only sparsely expressed (*beat-VI*). We found that at an earlier stage (36-48h APF), the expression of *beat-IIa*, *beat-IIb*, and *beat-VI* in several adult glomeruli has not yet been turned on, whereas *side* is already broadly expressed in the antennal lobe (Figure S6A, C). … We validated the PN expression of *side-IV* in the developing brain (36-48h APF), and found that it is expressed in fewer glomeruli than in the adult brain. Among these, glomeruli positive for *side-IV* in pupal stages appear to maintain expression throughout development, while others seem to be turned on in later stages (Figure S6B, C)."

Line 199-204, 340-343 in the change-tracked version.

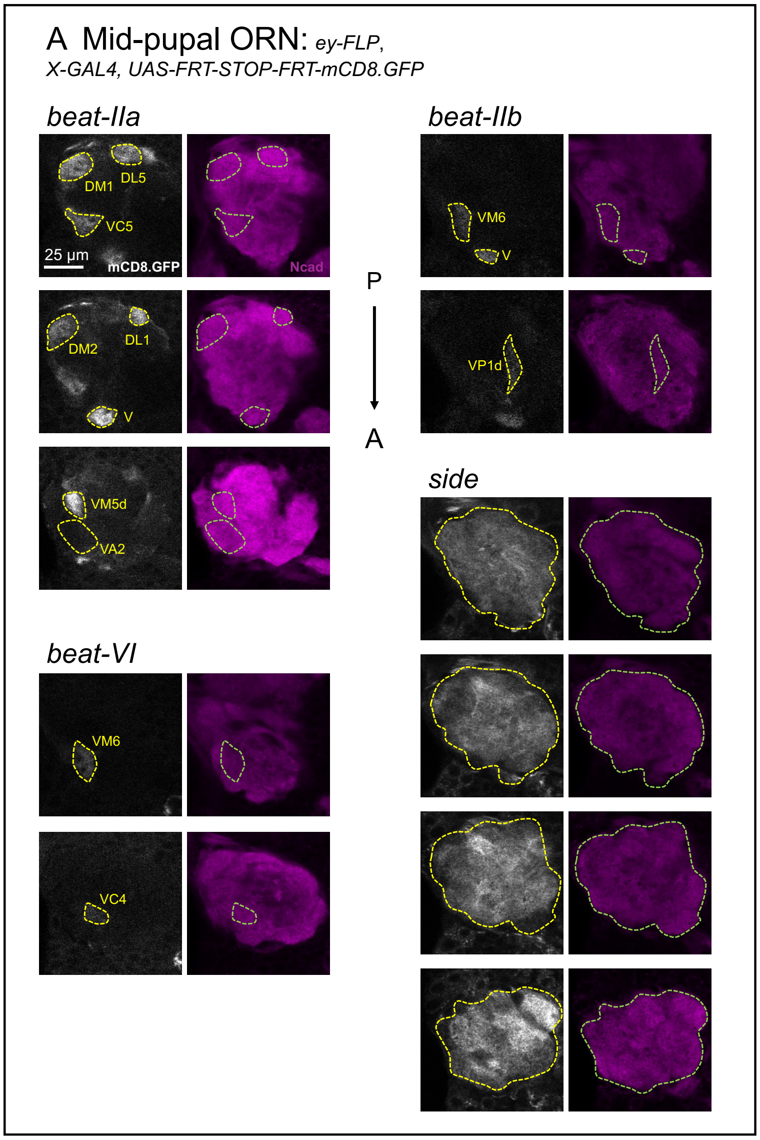

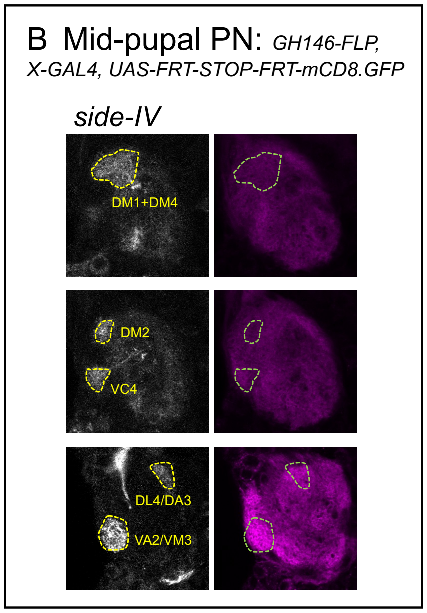

C

beat-IIa	Mid-pupal ORN (36~48h APF)	Adult ORN
DM1	✓	✓
DM2	✓	✓
DL1	✓	✓
DL5	✓	✓
V	✓	✓
VC5	✓	✓
VM5d	✓	✓
VC1	✗	✓
VC2	✗	✓
VA2	✗	✓

beat-IIb	Mid-pupal ORN (36~48h APF)	Adult ORN
VP1d	✓	✓
VM6	✓	✓
V	✓	✓
DC4, VA1d, VA1v, etc*	✗	✓

beat-VI	Mid-pupal ORN (36~48h APF)	Adult ORN
VM6	✓	✓
VC4	✓	✓
VC3	✗	✓
VM4	✗	✓
DL4	✗	✓

side-IV	Mid-pupal PN (36~48h APF)	Adult PN
DM1	✓	✓
DM2	✓	✓
DM4	✓	✓
VC4	✓	✓
VA2/VM3	✓	✓
DA3/DL4	✓	✓
DL1	✗	✓
VC2	✗	✓

Reviewer Figure 2-4 (new Figure S6). Characterization of the expression of several *beat/side MiMIC* drivers in the mid pupal stage. Related to Figures 2, 3. (A) ORN expression map of four *beat/side* genes in the developing brain. Single confocal sections are organized from the posterior side to the anterior side, with positive glomeruli highlighted by dashed lines. For *side*, which is expressed broadly across glomeruli, the outline of the whole antennal lobe is shown in dashed lines. **(B)** PN expression map of *side-IV* in the developing brain. Single confocal sections are organized from the posterior side to the anterior side, with positive glomeruli highlighted by dashed lines. **(C)** Comparison of the glomerular map of *beat/side* expression in mid-pupal and adult brains. ✓, detectable/expressed; ✗, undetectable/not expressed. * Full list of ORN glomeruli expressing *beat-IIb* in the adult but not mid-pupal brains: VP1m, VP2, VP3, VP4, DM1, DC4, DL1, DP1l, VC5, VM1, VL2a, DL2d, DL2v, VC3, VC1, VC2, VM4, DL4, DC3, DM5, VA1d, VA1v, VM5d, DA3, DA4m.

4) One has to remember that these mimic-Gal4s are not always faithful representation of the expression pattern and definitely say nothing about the localization of the proteins. Either using mimic-GFSTS or, even better - yet more time consuming, analysis of endogenously tagged

proteins (in sparse populations of neurons - for example see Sanfilippo et al 2024) will provide important data as to the true localization to the region of interest- in the case of the ORNs to their axons, and in the case of the PNs to their dendrites. As the authors write in the discussion, this is of particular interest in cases in which cognate Beats and Sides are co-expressed in the same PN/ORN - are they differentially distributed in axons vs. dendrites or do they co-localize to the same subcellular regions (perhaps suggestive of cis-inhibition)?

We thank the reviewer for raising this suggestion, which gives us an opportunity to provide more information about the *in vivo* expression of Beat/Side.

We added several lines of evidence to support the reliability of GAL4 lines. (1) For five genes, we happen to have gene trap lines from two independent MiMIC insertion sites. These lines showed consistent labeling patterns in the brain, which validates our method. We added a new supplementary figure (**new Figure S1**) to present these results. (2) We also tested several MiMIC-based protein trap lines in which a GFP tag is inserted into MiMIC sites within the coding introns, allowing direct visualization of those Beat/Side proteins. We also found a comparable labeling pattern between MiMIC-GFP and MiMIC-GAL4. For example, Side-GFP and side-T2A-GAL4-driven membrane marker show similar brain-wide expression patterns (**new Figure S9D**).

However, we noticed that MiMIC-GFP reagents are not always ideal, as some show the signal predominantly localized to cell bodies and barely detectable in neurites (**new Figure S9G**), likely owing to disrupted protein function caused by the inserted tag. These *MiMIC*-based GFP knock-in alleles also do not rescue the loss-of-function phenotype and are therefore maintained as heterozygotes. For example, the GFP tag is inserted inside the first Ig domain of Beat-VI, and it is very likely to misfold the protein and trigger ER retention. In contrast, the *MiMIC*-based GFP tag is inserted between two independently folded domains of Side-V (Ig5 and the fibronectin domain), which doesn’t seem to interfere with the function and localization of the Side-V protein.

Those good reagents indeed revealed the localization of Beats and Sides to the antennal lobe, but could not distinguish the cellular sources. This again highlights the importance of the genetic intersectional approach we used to precisely map the ORN and PN expression. By cross-validation with our GAL4 labeling data, we found that Beat and Side proteins could be expressed at both ORN terminals and PN dendrites, and perhaps also in the glomerular neurites of local interneurons. It is likely that interacting Beats and Sides could be colocalized in the same cell, as we mentioned in the Discussion. However, given the lack of appropriate MiMIC-GFP reagents, we could not systematically examine additional cognate Beat–Side pairs in this study.

Interestingly, a recent study showed that Kenyon cells of the mushroom body, which are postsynaptic to PNs, similarly express much fewer IgSF molecules (**Reviewer Figure 2-7D**), resembling ORNs that are presynaptic to PNs. These observations suggest that PNs, as the middle layer of the olfactory circuit, may require more diverse cell-surface repertoires to interact with numerous upstream and downstream partners, whereas their synaptic partner populations employ comparatively sparser and more selective molecular codes. It also suggests that Beat proteins expressed in PNs may additionally function at presynaptic axon terminals to interact with postsynaptic Kenyon cells, whereas some Side proteins may preferentially function on PN dendrites to interact with ORNs.

We thoroughly presented these results in a new section with the subhead “Expression of Beat/Side proteins in the antennal lobe” along with a new supplementary figure (**Figure S9**). We also extended the Discussion section accordingly.

In Results:

"Among these genes, we generated gene trap lines from two independent *MiMIC* insertion sites for five genes. These lines showed consistent labeling patterns in the brain, supporting the reliability of this method in faithfully reporting native gene expression (Figure S1)."

Line 153-156 in the change-tracked version.

New section in Results:

"Expression of Beat/Side proteins in the antennal lobe Recent proximity labeling-based cell-surface proteomic profiling found several Beasts/Sides are among the top proteins enriched on the ORN (Side-VI, Side-V, Side) and PN (Beat-IV, Side, Side-III, Side-VII, Beat-IIIC, Beat-VI) surface (Li et al., 2020; McLaughlin et al., 2026). To examine the *in vivo* glomerular expression of Beat/Side proteins, we used *MiMIC*-based protein-trap animals, where, instead of the *GAL4* cassette, a GFP protein tag (the full tag contains EGFP-FlAsH-StrepII-TEV-3xFlag, and here we term GFP for simplicity) is inserted into the *MiMIC* site as an artificial exon (Nagarkar-Jaiswal et al., 2015). We obtained a collection of available transgenic flies expressing *MiMIC-GFP*-tagged Beats/Sides. In particular, tagged Side protein exhibits very similar brain-wide localization patterns to that of the *side-T2A-GAL4*-driven membrane-bound myr.GFP reporter (Figure S9D), suggesting the reliability of these genetic reagents. Overall, we found signals for Beat/Side proteins in both presynaptic ORNs and postsynaptic PNs or local interneurons within the antennal lobe (Figure S9). For example, *side-VI* is broadly expressed across ORNs and PNs (Figure 2B, 3B) but is excluded in DM1-innervating ORNs. Concordantly, Side-VI-GFP signal is present in the ORN axonal commissure and shows lower yet still detectable expression in the DM1 glomerulus (Figure S9F). This suggests additional Side-VI protein localization to non-ORN neurites innervating the DM1 region, likely from PN dendrites or local interneurons. Another glomerulus, DC4, which is targeted by *side-VI*-positive ORNs, shows a marked Side-VI protein signal (Figure S9F). Beat-Va is an interacting partner of Side-VI and is not expressed in any ORNs based on the GAL4 labeling or RNA-seq results (Figure 2B; Figure S3B) but is expressed in many PN classes (Figure 3B). We also detected a Beat-Va protein signal across glomeruli, likely reflecting the neurite localization from PN or local interneurons (Figure S9C). Additionally, the *side-V* gene has a characteristic high expression in Gr21a ORNs, and the elevated signal of the tagged Side-V protein could also be detected in the corresponding V glomerulus (Figure S9E). Unlike *beat-Va*, many *beat* genes are expressed in both ORNs and PNs (Figure 2B, 3B). For example, we observed signals of Beat-IIb- and Beat-IV- tagged proteins in ventral PN (vPN) axons projecting to higher brain regions (Figure S9A, B). In parallel, the Beat-IV-GFP signal is also detected in ORN axons and pervades the antennal lobe, and is lower yet detectable in the DM1 glomerulus, which is negative for *beat-IV*-expressing ORNs (Figure S9B). It is noteworthy that these tagged proteins may not always reflect the native subcellular localization, depending on where the tag is inserted and whether the insertion interferes with the proper folding, processing, and trafficking of the native protein. We found several *MiMIC*-tagged Beat/Side proteins, including Beat-Ib, Beat-VI, Beat-VII, and Side-III, with GFP-tag signal predominantly localized to cell soma and barely detected in neurites (Figure S9G), likely owing to disrupted protein function by the inserted tag. In summary, we validated the *in vivo* subcellular localization of some Beat/Side proteins and confirmed their expression across glomeruli in the antennal lobe, supporting their functions as cell adhesion molecules to mediate the interactions between neurites in the antennal lobe."

Line 391-426 in the change-tracked version.

In Discussion:

"There are several possible functional explanations based on Beat and Side interactions. First, some Sides and Beats may have different subcellular localizations that position places of protein-protein

interactions and cellular functions. For example, Beats might localize to PN dendrites to interact with Sides on ORN axon terminals, while Side proteins localized to PN axon terminals may interact with Beats expressed on the dendrites of third-order neurons. And this asymmetric subcellular preference may vary in different cell types to mediate interactions with other neurons. In motor neurons, Beats are preferentially sorted to axon terminals, whereas in sensory neurons, some Sides are preferentially sorted to axon terminals. **Notably, a recent work found that in Kenyon cells of the mushroom body that receive inputs from olfactory PNs, many IgSF genes, including most *beats*, are not expressed, whereas many *sides* are (Thornton-Kolbe et al., 2025). This raises the possibility that some Beat proteins in PNs might also act on axon terminals to interact with postsynaptic Kenyon cells, while some Side family proteins are also required on the PN dendrites during ORN-PN matching.** Second, Beats/Sides can function in both dendrites and axons simultaneously and act as ligands and receptors bidirectionally. This points to a model in which the combinatorial code of multiple surface proteins establishes synaptic specificity recognition, likely by providing differential adhesive forces. **Indeed, we observed non-ORN signals for Side-VI and Beat-Va proteins in the antennal lobe, likely reflecting their colocalization on PN dendrites (Figure S9C, F).** Third, Beats and Sides in the same neuron might interact *in cis* on the same membrane in addition to their interactions *in trans* with Beat/Side proteins on other neurons. Interestingly, a recent study showed that DIP-Dpr interactions favor *cis* over *trans* when present in the same cell (Morano et al., 2025). It might also be the case that when Beats/Sides are localized together in the same membrane regions, it is the competition between *cis* and *trans* interactions with all present binding partners in the proximity that sets the “net” Beat/Side surface codes.”

Line 697-720 in the change-tracked version.

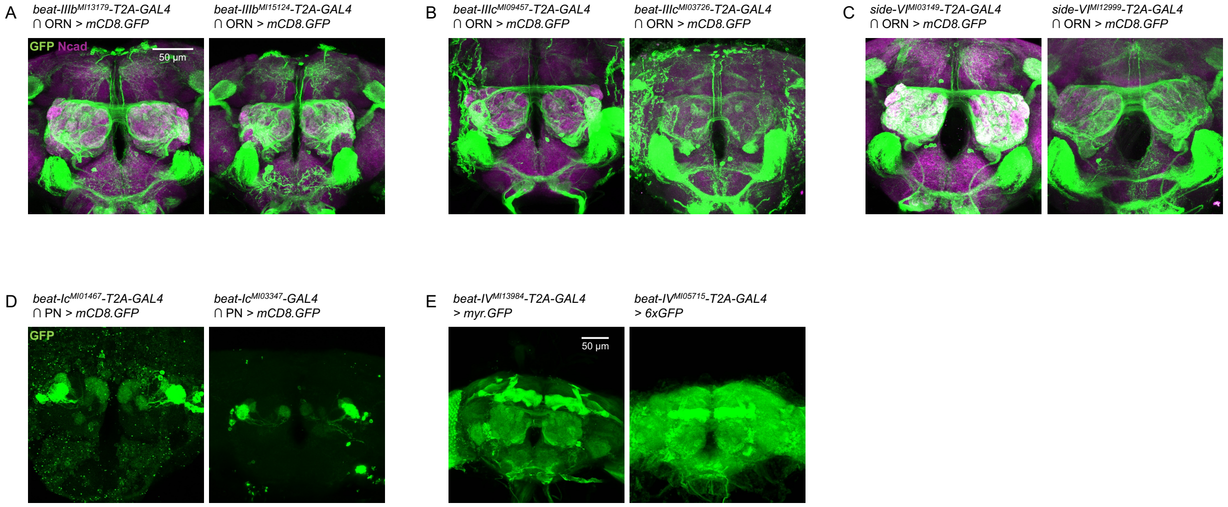

Reviewer Figure 2-5 (new Figure S1). Consistent expression patterns across different *MiMIC* driver lines for the same gene. Related to Figure 1. (A-E) Representative images showing brains labeled by two different *MiMIC*-based gene trap drivers targeting the same gene. Scale bar in (A) applies to images in (A) to (D). Insertion site for these additional *MiMIC* swap lines: *beat-IIIb*^{MI15124}: coding intron, targeting 2/2 isoforms; *beat-IIIc*^{MI09457}: coding intron, targeting 2/2 isoforms; *side-VI*^{MI12999}: coding intron, targeting 2/2 isoforms; *beat-Ic*^{MI01467}: coding intron, targeting 3/3 isoforms; *beat-IV*^{MI05715}: coding intron, targeting 2/2 isoforms.

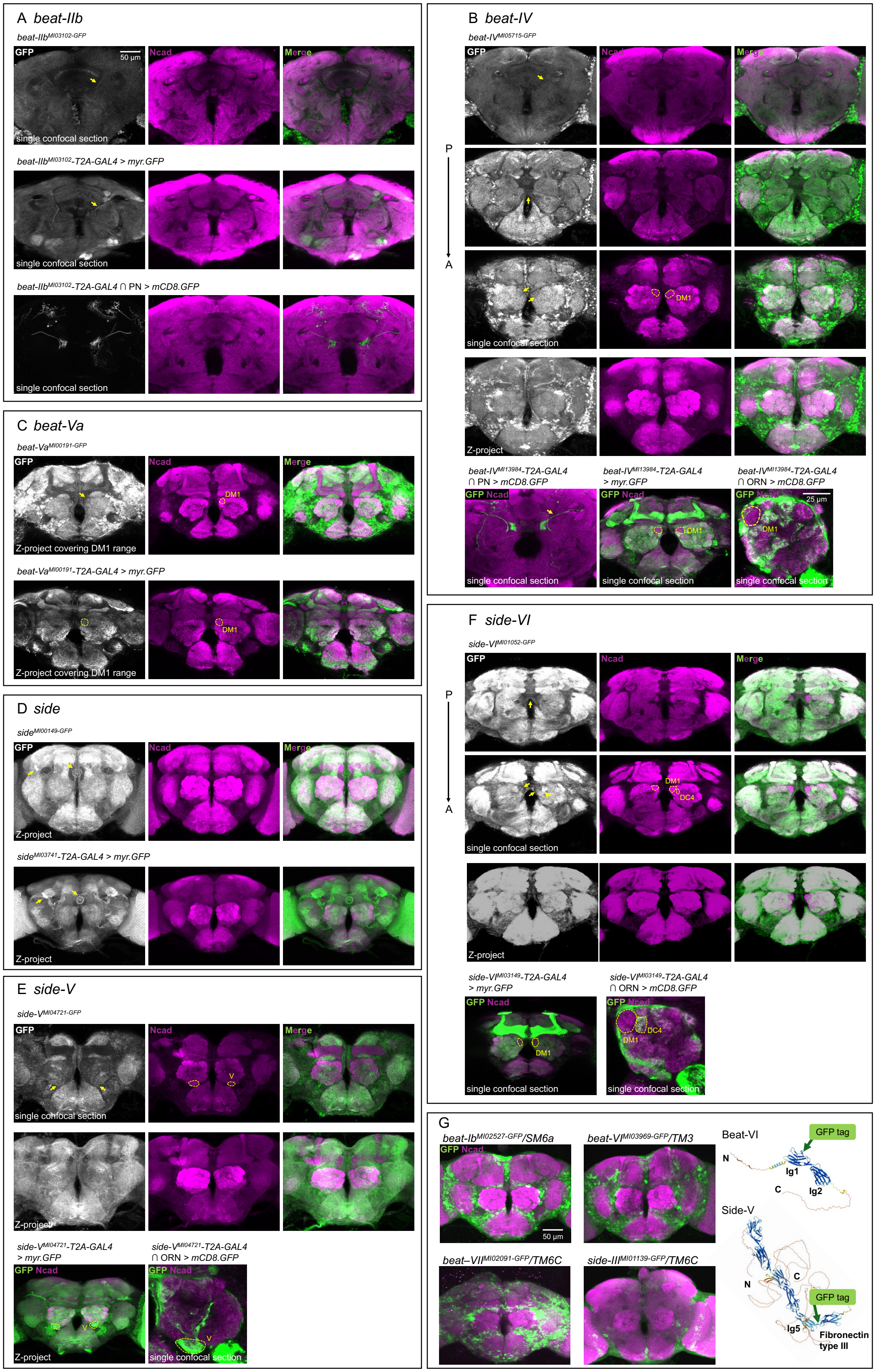

A beat-IIb
beat-IIbMI03102-GFP
GFP
50 µm
Ncad
Merge
single confocal section
beat-IIbMI03102-T2A-GAL4 > myr.GFP
single confocal section
beat-IIbMI03102-T2A-GAL4 ∩ PN > mCD8.GFP
single confocal section
B beat-IV
beat-IVMI05715-GFP
GFP
Ncad
Merge
P
A
DM1
single confocal section
Z-project
beat-IVMI13984-T2A-GAL4 ∩ PN > mCD8.GFP
beat-IVMI13984-T2A-GAL4 > myr.GFP
beat-IVMI13984-T2A-GAL4 ∩ ORN > mCD8.GFP
GFP Ncad
25 µm
DM1
single confocal section
C beat-Va
beat-VaMI00191-GFP
GFP
Ncad
Merge
DM1
Z-project covering DM1 range
beat-VaMI00191-T2A-GAL4 > myr.GFP
DM1
Z-project covering DM1 range
D side
sideMI00149-GFP
GFP
Ncad
Merge
Z-project
sideMI03741-T2A-GAL4 > myr.GFP
Z-project
E side-V
side-VMI04721-GFP
GFP
Ncad
Merge
V
single confocal section
Z-project
side-VMI04721-T2A-GAL4 > myr.GFP
side-VMI04721-T2A-GAL4 ∩ ORN > mCD8.GFP
GFP Ncad
V
Z-project
single confocal section
F side-VI
side-VIMI01052-GFP
GFP
Ncad
Merge
P
A
DM1
DC4
single confocal section
Z-project
side-VIMI03149-T2A-GAL4 > myr.GFP
side-VIMI03149-T2A-GAL4 ∩ ORN > mCD8.GFP
GFP Ncad
DM1
DC4
single confocal section
G
beat-IbMI02527-GFP/SM6a
beat-VIMI03969-GFP/TM3
GFP Ncad
50 µm
beat–VIIMI02091-GFP/TM6C
side-IIIMI01139-GFP/TM6C
Beat-VI
GFP tag
N
Ig1
C
Ig2
Side-V
N
C
GFP tag
Ig5
Fibronectin type III

Reviewer Figure 2-6 (new Figure S9). Characterization of the *in vivo* Beats/Side protein expression. Related to Figures 2, 3. (A-F) Example brain images showing the expression of the indicated Beat/Side protein, revealed by the MiMIC-based knock-in GFP tags. Images of *MiMIC-T2A-GAL4* driver-labeled brains are also shown for comparison. Scale bar (50 μm) in (A) applies to all rectangular brain images in (A) to (G). Scale bar (25 μm) in (B) applies to all square brain images in (B), (F), and (E). Yellow arrows in (A) denote axons of ventral PNs. Yellow arrows in (B) denote ventral PN axons, ORN axonal commissures, and the DM1 glomerulus, respectively, in *beat-IV*$^{MI05715\text{-}GFP}$ brains (from top to bottom). DM1 glomerulus, negative for *beat-IV* in ORNs, is also highlighted by dashed circles in other panels. Yellow arrows and dashed circles in (C) denote the DM1 glomerulus, which is negative for *beat-Va* in either ORN or PN. Yellow arrows in (D) denote two apparent brain structures that are consistently labeled by *in vivo* tagged Side proteins and *MiMIC* GAL4-driven membrane marker. Yellow arrows in (E) denote the V glomerulus that is labeled by *side-V*-expressing ORNs. V glomerulus is also highlighted by dashed circles in other panels. Yellow arrows in (F) denote ORN axonal commissures in the upper panels and DM1 and DC4 glomeruli in the lower panels showing the single confocal section of *side-VI*$^{MI01052\text{-}GFP}$ brains. DM1 (negative for *side-VI* in ORNs) and DC4 (positive for *side-VI* in ORNs) glomeruli are also highlighted by dashed circles in other panels. **(G)** Four representative fly brains carrying *MiMIC*-based GFP knock-in alleles, which do not rescue the loss-of-function phenotype and are therefore maintained as heterozygotes. The GFP signal is predominantly restricted to cell bodies. Upper right: AlphaFold-predicted structure of Beat-VI protein and the position where the *MiMIC*-based GFP tag is inserted. As the GFP tag is inserted inside the first Ig domain of Beat-VI, it very likely misfolds the protein and triggers ER retention. Lower left: AlphaFold-predicted structure of Side-V protein and the position where the *MiMIC*-based GFP tag is inserted. The GFP tag is inserted between two independently folded domains of Side-V (Ig5 and the fibronectin domain), which doesn't seem to interfere with the function and localization of the Side-V protein.

Mechanistic pillar:
Well - here the data is just very weak. suggestions for improving:

RNAi using pan-ORN or almost pan-PN drivers is not very specific and not surprisingly yielded in nothing interesting. I encourage the authors to use MARCM and mutants (for what's available) which allows a much more careful analysis of misrouting. In case of using RNAi against side - to the best of my knowledge an antibody exists and is readily available. Finally, due to known

In this case, a GOF experiment could actually be very informative - over expression of one of the sparsely expressed proteins in an inappropriate neuron and see if it is mistargeted appropriately. Obviously, this needs to be planned carefully to make sure the experiment is informative.

Taking reviewers' suggestions, we added additional functional investigation data to the revised paper:

(1) For our initial screening on glomerular targeting (V, VA1v, DM3 glomeruli), we used two different approaches to perturb each gene in ORNs, either by two independent RNAi lines or one RNAi line and one gRNA line, which induces somatic knockout. This screen revealed mild defects in the DM3 glomerulus, due to loss of *beat-IIIc*, and in the VA1v glomerulus, due to loss of *beat-IIb*.

(2) We also tried simultaneous knockout of two co-expressed genes, *beat-IIa* and *beat-IIb*, in ORNs using tissue-specific CRISPR and examined the corresponding glomerular targeting (the V glomerulus), though we didn't observe any obvious defect in that glomerulus.

(3) We tried over-expression of three sparsely or minimally expressed genes, *beat-Ib*, *beat-Vc*, *beat-VI*, respectively, in ORNs and examined glomerular targeting (V, VA1v, DM3 glomeruli). Unfortunately, these perturbations didn't give rise to any apparent wiring defects.

(4) For the Beat-IIa/Side-IV pair in DM1 and VA2 glomeruli, the RNAi reagents we used were also used with reported phenotypes in other published studies in the Drosophila visual system.

While these observations apply to the limited glomerular types we examined, it is entirely possible that stronger or more specific defects may emerge in other ORN/PN types with different combinations of *beat/side* perturbations. Indeed, our expression dataset suggests several intriguing candidate combinatorial manipulations of multiple *beat/side* genes in specific glomeruli. However, systematically testing these possibilities is technically demanding, which requires significant time to generate new transgenic reagents and extensive genetic crosses to assemble complex perturbation genotypes. We feel like this is beyond the scope of this paper.

We rewrote the paragraphs about the perturbation experiments related to the **new Figure S10**:

"To test whether *beat/side* plays any role in glomerular targeting or organization, we conducted a pan-ORN *peb-GAL4*-driven *UAS-RNAi* screening against *beat* and *side* genes exhibiting decent expression in specific ORNs and examined whether three glomeruli would show any defects as a readout. These include Gr21a ORNs, Or47b ORNs, and Or47a ORNs, targeting V, VA1v, and DM3 glomerulus, respectively (Figure S10A). We included perturbation of *side-VIII*, which is not expressed in any ORNs, as an additional negative control. For each gene perturbed, we used two independent approaches: either two different *RNAi* lines to knock down or one *RNAi* line and one gRNA line inducing a somatic knockout (Figure S10B). First, we noticed that V glomerulus appeared wild-type in all perturbations (Figure S10A). Second, this screen revealed local organizational defects in the VA1v glomerulus by knockdown of *beat-IIb, beat-IIIa/b/c,* and *side-III*. Among these, the VA1v phenotype was reproduced by two independent *RNAi* lines against *beat-IIb*, but the defects in *beat-IIIa/b/c* or *side-III* knockdowns could not be robustly observed in secondary perturbations (Figures S10B). Additional controls showed that the VA1v phenotypes of *beat-IIIa/b/c* knockdowns appeared to arise from the transgenic background that sensitizes the VA1v glomerular disorganization (Duan et al., 2023)(Figure S4C). Third, we observed a low-penetrance phenotype of split DM3 glomerulus, which appeared in both *RNAi* knockdown and gRNA-mediated ORN-specific knockout of *beat-IIIc* (Figure S10A, B, E). Overall, we identified two hits with mild glomerular phenotypes from this targeted screen: *beat-IIb* in Or47b ORNs and *beat-IIIc* in Or47a ORNs. However, Beat-IIb-interacting Side-IV is not expressed in postsynaptic PNs innervating the VA1v glomerulus or nearby regions. Coincidentally, *side-IV* is restrictedly expressed in Or47b and Or88a ORNs, which target a neighboring glomerulus (Figure 2A, B), raising the possibility for axon-axon contacts mediated by co-expressed Beat-IIb and Side-IV, which is interesting for future exploration. Additionally, Beat-IIIc is an orphan cell-surface protein with no characterized interacting partners (Figure 1A), which limits our further investigation of its role in glomerular organization. Moreover, we made a preliminary effort with multiplexing perturbation. We found that *beat-IIa* and *beat-IIb* are markedly co-expressed in the V glomerulus-innervating ORNs. Side-IV, which interacts with Beat-IIa and Beat-IIb, appears to be expressed in PNs targeting the V glomerulus as well (Figure S10F). We therefore used ORN-specific CRISPR to simultaneously knock out *beat-IIa* and *beat-IIb but* didn't find an overtly defective glomerular organization (Figure S10F). In addition, we also performed gain-of-function experiments using pan-ORN overexpression of *beat* genes that are sparsely expressed across ORNs, but did not observe any obvious mistargeting phenotypes (Figure S10G).

We then turned to other glomeruli with the matching expression of interacting Beats and Sides between partner ORN/PN classes. We found one straightforward case: presynaptic Beat-IIa and postsynaptic Side-IV. *beat-IIa* is expressed in just a few ORN classes, including Or42b ORNs targeting one posterior glomerulus DM1 and Or92a ORNs targeting one anterior glomerulus VA2 (Figure 4B). In parallel, *side-IV* expression is restricted in PNs, including PNs projecting to DM1 and VA2 glomeruli (Figure 4C). Next, we tested whether perturbing *beat-IIa* expression in ORNs or *side-IV* expression in PNs results in glomerular

targeting defects, using the *RNAi* lines validated with reported phenotypes in previous studies (Jain et al., 2022; Osaka et al., 2024).”

Line 440-479 in the change-tracked version.

Informed by our expression atlas, we specifically proposed experiments that are worthwhile to pursue in the future:

“Our expression dataset provides a roadmap for further cracking the combinatorial coding nature of Beats and Sides through multiplexed perturbation. For example, DM1 and DM2 glomeruli are adjacent to each other; DM2 ORNs might distinguish themselves from DM1 ORNs by Side-VI, which interacts with Beat-Va/Vc expressed by DM2 PNs, whereas these interactions don’t exist between DM1 ORNs and PNs (Figure 4H, I). In parallel, DM1 ORNs express the additional molecule Beat-IIb, potentially strengthening their interactions with DM1 PNs relative to DM2 ORNs (Figure 4H, I). Together, these differential combinatorial interactions may help reinforce the specificity of ORN–PN matching between neighboring glomeruli. Accordingly, combinatorial manipulations that disrupt these biased recognition cues could potentially lead to miswiring between DM1 and DM2 ORN–PN pairs.”

Line 735-742 in the change-tracked version.

In terms of the antibody again Side, we found a discrepancy for staining patterns. A recent study (Thornton-Kolbe et al., 2025) reported that many IgSF genes, including *side*, are not expressed in Kenyon cells of the Mushroom Body (**Reviewer Figure 2-7D**). This is consistent with both our MiMIC-GAL4 and MiMIC-GFP data for *side*, where the signal is absent in the Mushroom Body lobes (**Reviewer Figure 2-7B, C**). But anti-Side antibody detects an apparent signal across the Mushroom Body (**Reviewer Figure 2-7A**), suggesting nonspecific staining of this antibody. We therefore didn’t include the antibody staining data in our manuscript.

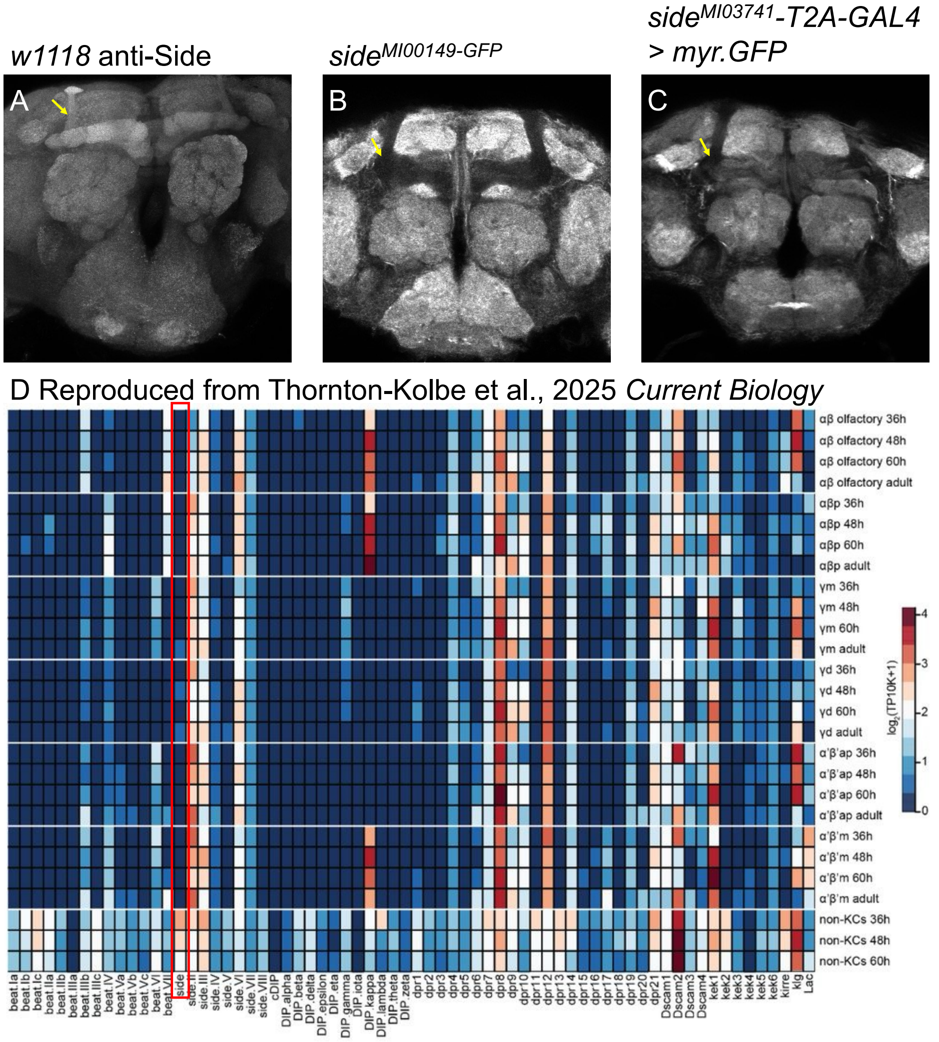

Reviewer Figure 2-7. (A) Anti-Side staining for a w1118 brain. (B) Anti-GFP staining for a *side*$^{MI00149\text{-}GFP}$ brain. (C) Anti-GFP staining for a *side-T2A-GAL4*-driven membrane-bound GFP brain. Yellow arrows in (A), (B), and (C) indicate the mushroom body lobes where Kenyon cells' neurites innervate. (D) Heatmap showing the expression of IgSF genes in the Mushroom Body neurons throughout development. Note that the *side* gene is largely depleted in all Kenyon cells except the γ subtype (red rectangle).

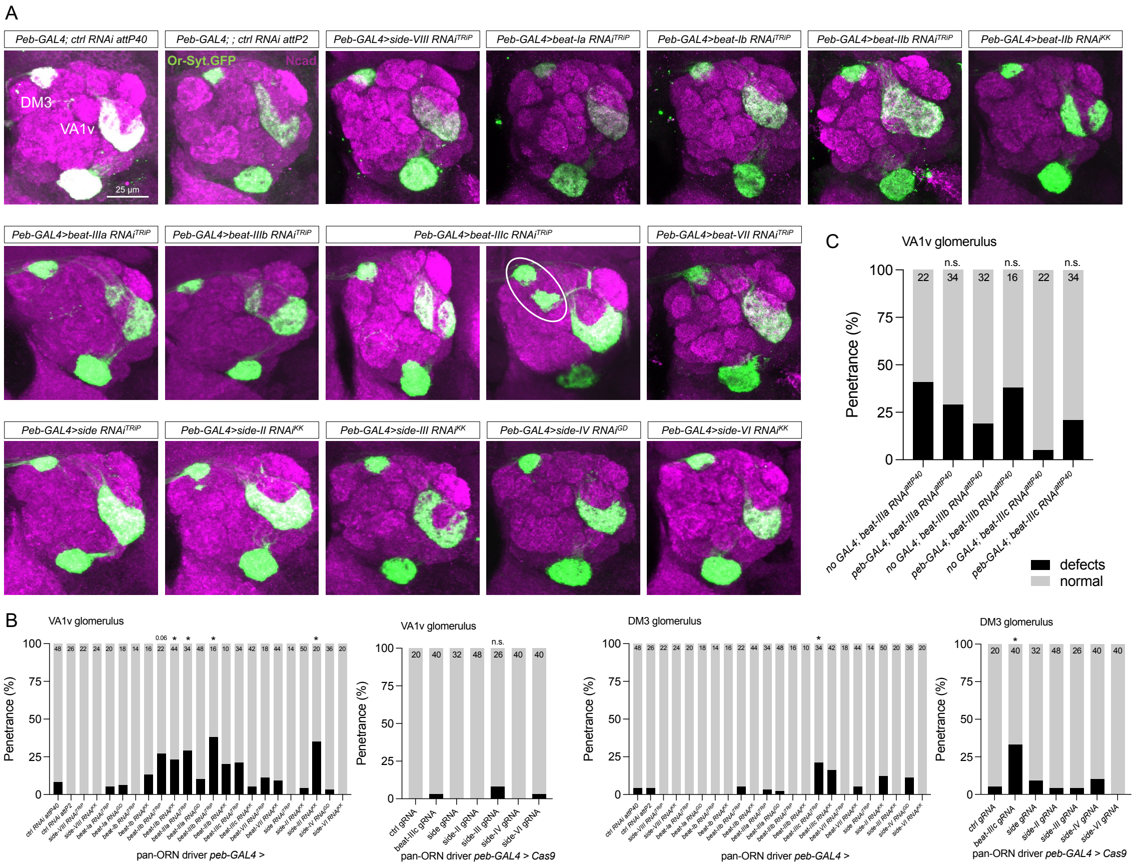

D

Expression pattern	Gene
Not expressed anywhere (negative control)	*side-VIII*
Not expressed in VA1v but expressed in neighboring glomeruli of VA1v	*beat-Ia*
Expressed in VA1v	*beat-Ib, beat-IIb, beat-IIIa, beat-IIIb, beat-IIIc, beat-IV*, beat-VII, side, side-II, side-III, side-IV, side-V*, side-VI*
Not expressed in VA1v or any nearby glomeruli (not tested)	*beat-Ic, beat-IIa^, beat-Va, beat-Vc, beat-VI*

Expression pattern	Gene
Not expressed anywhere (negative control)	*side-VIII*
Not expressed in DM3 but expressed in neighboring glomeruli of DM3	*beat-IIa^, beat-IIb, beat-Vc, side, side-III, side-V**
Expressed in DM3	*beat-Ia, beat-IIIa, beat-IIIb, beat-IIIc, beat-IV*, beat-VII, side-II, side-VI*
Not expressed in DM3 or any nearby glomeruli	*beat-Ib, beat-Ic, beat-Va, beat-VI, side-IV*

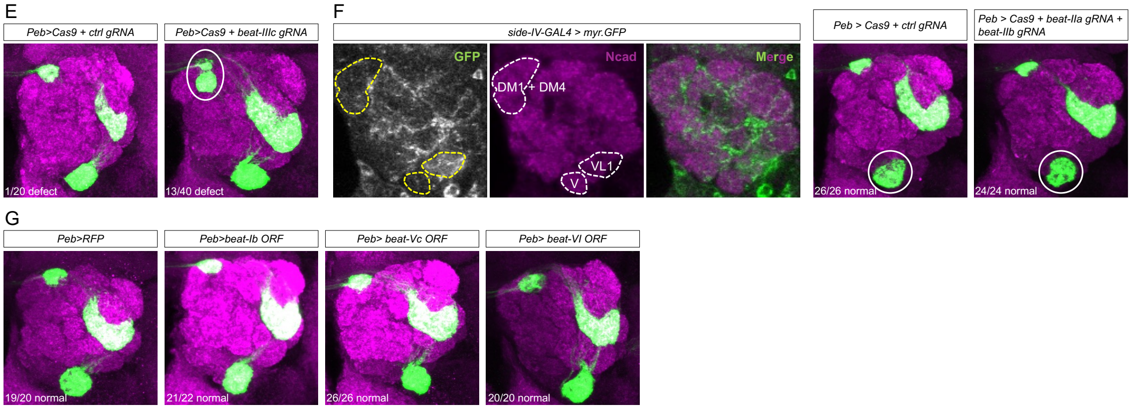

Reviewer Figure 2-8 (new Figure S10). Perturbation of *beats/sides* in ORNs leads to little to no defect in glomerular organization. Related to Figure 4. (A) Representative antennal lobes of controls and knockdowns of *beat/side* genes. A split phenotype of the DM3 glomerulus is highlighted with a solid circle. **(B)** Quantification of the percentage of lobes showing glomerular defects of VA1v and DM3 glomeruli in each genetic condition. The number of antennal lobes examined is shown in each bar. P

values were determined by comparison to control groups. *, $p<0.05$. n.s., not significant. **(C)** Quantification of the percentage of lobes showing glomerular defects of VA1v glomerulus of *beat-IIIa/b/c* perturbation with additional *RNAi* transgenic background controls. The number of antennal lobes examined is shown in each bar. n.s., not significant. **(D)** Summary of expression of each gene regarding to VA1v and DM3, respectively. * Knockdown of *beat-IV* or *side-V* with this *peb-GAL4* driver caused lethality. ^ Knockdown of *beat-IIa* was preliminarily tested for DM3 phenotype, but all nine brains examined appeared normal. **(E)** Representative antennal lobes showing the split DM3 glomerulus in the conditional knockout of *beat-IIIc* in ORNs, consistent with the *beat-IIIc RNAi* perturbation. **(F)** Representative antennal lobes showing that simultaneous knockout of *beat-IIa* and *beat-IIb* in ORNs didn't cause a gross targeting phenotype of V glomerulus. A single confocal section of *side-IV MiMIC* GAL4-driven GFP expression in the antennal lobe is also shown to indicate that *side-IV* is likely to be expressed in PNs innervating the V glomerulus, comparable to the positive glomeruli DM1 and DM4 (Figure 4A, C; Figure S6B). **(G)** Representative antennal lobes showing that the ectopic expression of three *beats* didn't produce an apparent glomerular targeting defect. These three *beat* genes, *beat-Ib*, *beat-Vc*, and *beat-VI*, are either barely expressed in ORNs or expressed only in a few ORN classes.

Evolutionary pillar:

I am not an expert here - but as it stands now it does not add much to the story. Either make a full story out of it or find a way to integrate into your study that makes sense. Does this part relate to your title, to your abstract? In its current form - the answer is no.

We thank the reviewer for raising this concern. In the revised manuscript, we refined the text to more clearly connect the evolutionary analyses with our expression analyses and overall conceptual framework in Drosophila. We view the evolutionary component as closely integrated with the central story rather than as an independent direction.

First, although our perturbation experiments in Drosophila yielded relatively mild or negative wiring phenotypes, the conservation of *beat/side* expression patterns across divergent insect species suggests that the regulation of these genes is under evolutionary selection pressure, implying functional relevance in ORN development and/or circuit assembly. In this sense, the evolutionary conservation provides indirect evidence supporting functional roles of Beats/Sides and complements the limited phenotypic outcomes observed in our current functional analyses.

Second, together with the lineage-correlated combinatorial organization observed in Drosophila, the cross-species conservation supports the idea that developmental programs coordinately regulate duplicated cell-surface recognition molecules during neural circuit assembly. We therefore believe the evolutionary analyses strengthen, rather than distract from, the main conceptual framework proposed in this study.

Finally, we also believe that systematically documenting *beat/side* orthologs and their expression patterns in emerging insect model systems such as ants and mosquitoes provides a valuable resource for future comparative and functional studies.

We have revised the manuscript to better emphasize these connections and to integrate the evolutionary findings more cohesively into the overall narrative.

In Abstract:

"This also implies the biological significance of *beats/sides* in ORN circuit development or function, which is preserved under selective pressure across divergent insect lineages."

Line 51-53 in the change-tracked version.

In Results:

“These comparative analyses suggest that the conserved and species-specific expression patterns of *beat* and *side* genes in peripheral sensory neurons likely arose through evolutionary events in the genome, including the duplication and loss of *beat/side* family members, as well as the conservation and divergence of their regulatory sequences. Importantly, the striking conservation of *beat* and *side* expression patterns in ORNs across species suggests that these expression programs are under selective pressure, supporting their functional relevance in ORN circuit development and/or operation.”

Line 594-600 in the change-tracked version.

Textual issues:

*Last sentence of the introduction: "Overall, our study reveals the combinatorial expression pattern and functional role that Beat/Side proteins play in olfactory circuit assembly and synaptic development" - I would delete the functional role, this is an overstatment (as the only substantial piece of data is the reduced synapses in Side KD)

We have reworded this sentence as follows:

“Overall, our study reveals the combinatorial expression profile of *beats/sides* in the fly olfactory circuit and sets a foundation for further functional investigation of interacting Beat/Side proteins in neuronal circuit assembly and synaptic development.”

Line 137-138 in the change-tracked version.

*Determining that "these results suggest that either Beat-IIa/Side-IV interaction in matching ORNs and PNs is unnecessary for glomerular targeting or is compensated by other Beat and Side proteins with redundant functions expressed in the same neurons" based on the one experiment is problematic. What if these RNAi lines are simply inefficient as often happens? see my other suggestions regarding mutant analysis etc.

We agree that RNAi efficiency is an important consideration when interpreting negative or mild phenotypes. In this study, we attempted to mitigate this concern by using two independent perturbation strategies for each candidate gene whenever possible, including either **two independent RNAi lines** or **one RNAi line together with a gRNA-mediated somatic knockout** approach. In addition, for the Beat-IIa/Side-IV experiments, we specifically selected RNAi lines that had previously been validated and shown to produce phenotypes in published studies.

We acknowledge that directly measuring knockdown efficiency using FISH or related methods would further strengthen the interpretation of individual perturbations. However, given the screening-based nature of this study and the large number of candidate genes examined, systematically validating the efficiency of all perturbations that produced negative results would

be technically challenging and beyond the scope of the current work. Instead, our strategy was to prioritize positive hits for follow-up analyses.

In the text, we cited the previous studies that used the same reagents with reported phenotypes:

"Next, we tested whether perturbing *beat-IIa* expression in ORNs or *side-IV* expression in PNs results in glomerular targeting defects, using the *RNAi* lines validated with reported phenotypes in previous studies (Jain et al., 2022; Osaka et al., 2024)."

Line 478-479 in the change-tracked version.

Reviewer #3:

Review of Duan et al. paper
This paper presents a remarkable compendium of information on expression of Beat and Side genes in the olfactory system. It will be the definitive source for expression data on these IgSF subfamilies in this system. It also describes new tools for evaluating expression of these genes, in that they have generated GAL4 drivers for almost all of them. The paper is essentially descriptive, because knockdown of the genes did not produce wiring phenotypes, although Side knockdown did lead to a general decrease in Brp labeling, likely reflecting a decreased number of presynapses.
I looked through all the figures and really have no technical criticisms of the work. I am sure it must have been disappointing, although perhaps not surprising, that they did not observe wiring defects in knockdowns of Beat-IIa and Side-IV. The olfactory and visual systems have a high degree of redundancy in synaptic targeting cues, and high-penetrance wiring phenotypes are seldom observed for single-gene perturbations. One has to survey many cell types to find one that is affected by a loss of expression of a single IgSF gene. For example, in Xu et al. 2018, loss of DIP-alpha in Dm4 neurons causes cell death but no targeting defects. Loss of DIP-alpha in Dm12 neurons, however, causes some of their dendrites (~20%) to project to the wrong layer of the medulla. In work from the Luo group, they find that complex combinatorial perturbations are required to generate high-penetrance wiring alterations in the antennal lobe. It is likely that Beat-IIa and Side-IV are in fact involved in glomerular targeting but are redundant with other, unknown cues. I would suggest that the authors also examine null CRISPR mutants of the genes in case knockdown is not strong enough. If even a few axons mistarget that would provide important evidence. They should also examine the scRNA datasets to find other genes that might be involved in wiring of these glomeruli, and perhaps combinatorial knockdown of these genes together with Beat-IIa and/or Side-IV might produce defects. They could also conduct gain-of-function studies. In Xu et al. 2018, they showed that, although loss of DIP-alpha's ligands Dpr6 and Dpr10, like loss of DIP-alpha, does not cause Dm4 wiring defects, high-level expression of Dpr10 in medulla layer M10 causes all Dm4 processes to project to M10, showing that Dpr10 is in fact a targeting cue for Dm4. However, given the large amount of work that has gone into this paper and the difficulty in finding interacting genes, this is beyond the scope of the present paper.

We thank the reviewer for the positive evaluation of our study and for recognizing the value of establishing a comprehensive expression atlas of *beat* and *side* genes in the olfactory system. We are also grateful for the reviewer's thoughtful comments regarding the challenges of

studying highly redundant cell-surface recognition systems and the importance of interpreting negative phenotypes cautiously.

Following other reviewers' suggestions, we expanded our expression analyses and provided new *in vivo* protein expression data. We hope the reviewer finds that our revision has been substantially strengthened.

We also added new functional investigation data. In particular, for our initial perturbation screen, we tested multiple independent perturbation strategies for candidate genes, including combinations of RNAi and gRNA-mediated somatic knockout approaches. We also tried multiplex perturbation experiments by simultaneously knocking out co-expressed *beat-IIa* and *beat-IIb* in ORNs using somatic CRISPR. In addition, we carried out gain-of-function experiments by overexpressing sparsely expressed *beat* genes in ORNs. While these additional experiments did not reveal strong wiring phenotypes, we agree with the reviewer that such negative or mild phenotypes are consistent with substantial redundancy within combinatorial cell-surface recognition systems.

We also appreciate the reviewer's suggestions regarding additional candidate genes and combinatorial perturbations that may reveal stronger phenotypes. Indeed, our expression atlas highlights many interesting candidate interactions that could be pursued in future studies. However, as the reviewer also noted, systematically testing these possibilities would require extensive generation of new reagents and complex genetic combinations and is beyond a reasonable timeframe of this revision and the scope of the current study. Nonetheless, we hope that the expression resource and conceptual framework established here will facilitate future mechanistic investigations into combinatorial Beat/Side functions in the olfactory circuit assembly.

References:

Barish, S., and P.C. Volkan. 2015. Mechanisms of olfactory receptor neuron specification in Drosophila. *Wiley Interdiscip Rev Dev Biol*. 4:609-621.

Brahma, A., D.D. Frank, P.D.H. Pastor, P.K. Piekarski, W. Wang, J.D. Luo, T.S. Carroll, and D.J.C. Kronauer. 2023. Transcriptional and post-transcriptional control of odorant receptor choice in ants. *Curr Biol*. 33:5456-5466 e5455.

Duan, Q., R. Estrella, A. Carson, Y. Chen, and P.C. Volkan. 2023. The effect of Drosophila attP40 background on the glomerular organization of Or47b olfactory receptor neurons. *G3 (Bethesda)*.

Hong, W., and L. Luo. 2014. Genetic control of wiring specificity in the fly olfactory system. *Genetics*. 196:17-29.

Jain, S., Y. Lin, Y.Z. Kurmangaliyev, J. Valdes-Aleman, S.A. LoCascio, P. Mirshahidi, B. Parrington, and S.L. Zipursky. 2022. A global timing mechanism regulates cell-type-specific wiring programmes. *Nature*. 603:112-118.

Li, J., S. Han, H. Li, N.D. Udeshi, T. Svinkina, D.R. Mani, C. Xu, R. Guajardo, Q. Xie, T. Li, D.J. Luginbuhl, B. Wu, C.N. McLaughlin, A. Xie, P. Kaewsapsak, S.R. Quake, S.A. Carr, A.Y. Ting, and L. Luo. 2020. Cell-Surface Proteomic Profiling in the Fly Brain Uncovers Wiring Regulators. *Cell*. 180:373-386 e315.

Liu, C., O. Trush, X. Han, M. Wang, R. Takayama, T. Yasugi, T. Hayashi, and M. Sato. 2020. Dscam1 establishes the columnar units through lineage-dependent repulsion between sister neurons in the fly brain. *Nat Commun*. 11:4067.

Lv, X., S. Li, J. Li, X.Y. Yu, X. Ge, B. Li, S. Hu, Y. Lin, S. Zhang, J. Yang, X. Zhang, J. Yan, A.L. Joyner, H. Shi, Q. Wu, and S.H. Shi. 2022. Patterned cPCDH expression regulates the fine organization of the neocortex. *Nature*. 612:503-511.

McLaughlin, C.N., M. Brbic, Q. Xie, T. Li, F. Horns, S.S. Kolluru, J.M. Kebschull, D. Vacek, A. Xie, J. Li, R.C. Jones, J. Leskovec, S.R. Quake, L. Luo, and H. Li. 2021. Single-cell transcriptomes of developing and adult olfactory receptor neurons in Drosophila. *Elife*. 10.

McLaughlin, C.N., H. Ji, K.X. Dong, C. Xu, K.K.L. Wong, Z. Li, D.J. Luginbuhl, C. Xu, C. Lyu, W. Qin, J. Li, N.D. Udeshi, S.A. Carr, A.Y. Ting, and L. Luo. 2026. Endocytome profiling uncovers cell-surface protein dynamics underlying neuronal connectivity. *Neuron*.

Morano, N.C., D.H. Lopez, H. Meltzer, A.P. Sergeeva, P.S. Katsamba, K.D. Rostam, H.P. Gupta, J.E. Becker, B. Bornstein, F. Cosmanescu, O. Schuldiner, B. Honig, R.S. Mann, and L. Shapiro. 2025. Members of the DIP and Dpr adhesion protein families use cis inhibition to shape neural development in Drosophila. *PLoS Biol*. 23:e3003030.

Nagarkar-Jaiswal, S., P.T. Lee, M.E. Campbell, K. Chen, S. Anguiano-Zarate, M.C. Gutierrez, T. Busby, W.W. Lin, Y. He, K.L. Schulze, B.W. Booth, M. Evans-Holm, K.J. Venken, R.W. Levis, A.C. Spradling, R.A. Hoskins, and H.J. Bellen. 2015. A library of MiMICs allows tagging of genes and reversible, spatial and temporal knockdown of proteins in Drosophila. *Elife*. 4.

Osaka, J., A. Ishii, X. Wang, R. Iwanaga, H. Kawamura, S. Akino, A. Sugie, S. Hakeda-Suzuki, and T. Suzuki. 2024. Complex formation of immunoglobulin superfamily molecules Side-IV and Beat-IIb regulates synaptic specificity. *Cell Rep*. 43:113798.
Task, D., C.C. Lin, A. Vulpe, A. Afify, S. Ballou, M. Brbic, P. Schlegel, J. Raji, G. Jefferis, H. Li, K. Menuz, and C.J. Potter. 2022. Chemoreceptor co-expression in Drosophila melanogaster olfactory neurons. *Elife*. 11.
Thornton-Kolbe, E.M., M. Ahmed, F.R. Gordon, B. Sieriebriennikov, D.L. Williams, Y.Z. Kurmangaliyev, and E.J. Clowney. 2025. Spatial constraints and cell surface molecule depletion structure a randomly connected learning circuit. *Curr Biol*. 35:3191-3208 e3110.
