# Supplementary material for: Beat- and Side-family cell-surface molecules are expressed combinatorially in the partner neurons of the olfactory circuit in Drosophila
Source: PLoS Biol. 2026 Aug 27;24(8):e3003955. doi: 10.1371/journal.pbio.3003955 (PMC13521578; doi:10.1371/journal.pbio.3003955)
Supplement: Supplementary file 3 [file pbio.3003955.s021.pdf]

!!

"#!$%&'! ( )#*'+,&-$!. /"010 2345467489: ;8<= ;!

^6_! Z ?!)›'C!$?'$?@!'?L?,)›! J & J "K4D)'?@!-,C$?&#!$,)-!›&#?'!&#!V%&+%!)!2S.!$)B!&'!&#'?,$?@!&#$C! J & J "K!'&$?'!V&$%&#!$%?!+C@&#B!&#$,C#'I!)»CV&#B!

! "#$"%"&'($)*&"'+,B'/0"%'($)*&"'1C2-'3>9&9:6"&$D96$40'4<'6>"

!

"#!$%&'!

Z ?!)+U#CV›?@B?!$%)$!

!

S&B*,?! : NI!$%?!

- , ?›ℓ (ℓ#) , F_

Z ?!$%) #U!

5?›L?!@??-?,]!!

9_!$%&'!&'!)!VC,U!C#!

!

! "#$"%"&' ($) *&"'. ,J'/0"%' ($) *&"

!

"#!=?'*›$']!!

! "#$"%"&

^9_! Z ?!

1&#?!7 ; 479!&#!$%?!+%)#B?4$,)+U?@!L?, '&C#R!!

!

"#!=?'*›$']!!

0$12 *!"#$%*

!

D?!$?+%#&+)»F!+%)»?#B&#B!)#@!D?FC#@!$%?!'+C-?!CG!$%?!+*,,?#$!VC,UR!"#'$?)@I!C*,!'$,)$?BF!V)'!
$C!-,&C,&$&P?!-C'&$&L?!%&$'!GC,!GC»CV4*-!)#)›F'?'R!!

"#!

'$*@F&#B!%&B%›F!,?@*#@)#$!+?»4'*,G)+?!,?+CB#&$&C#!'F'$? ('!)#@!$%?!& (-C,$)#+?!CG!&#$?,-,?$&#B! #?B)$&L?!-%?#C$F-?'!+)*$&C*'›FR!!

SC»CV&#B!C$%?,!,?L&?V?,'[!'*BB?'$&C#'I!

! "#"$"%&" '())

Z0-<-2!P42!J4!e01//2!b4!N-5O2!T4!e \ -5-O-2!L4!M- \ -@E.-2!34!J</5:2!J4!3EO/>2!34!L-<>6-I3EYE</2!
-56!R4!3EYE</4!#+#%4!8:@D;>d!A:.@-B/:5!:A!/@@E5:O;:XE;/5!0ED>.A-@/;C!@:;>?E;>0!
3/6>Ie9!-56!,>-BIeeX!.>OE;-B>0!0C5-DB/?!0D>?/F?/BC4! 4!%$H""$(*)4!
R-0<2!G42!848 4!Q/52!J4!9E;D>2!J4!JFAC2!34!,-;;:E2!=4!,.X/?2!74!3?1;>O>;2!P4!T-c/2![4!P>W>./O2!L4!Q/2!
