# Supplementary material for: Beat- and Side-family cell-surface molecules are expressed combinatorially in the partner neurons of the olfactory circuit in Drosophila
Source: PLoS Biol. 2026 Aug 27;24(8):e3003955. doi: 10.1371/journal.pbio.3003955 (PMC13521578; doi:10.1371/journal.pbio.3003955)
Supplement: Peer review history

# Author comment: Author response to Decision Letter 0

**DOI:** 10.1371/journal.pbio.3003955.r001

1 Jun 2026

Attachment Submitted filename: Response_Duan2025_BeatSideORNs_PlosBio_revision_v1.pdf

# Editor report: Decision Letter 0

**DOI:** 10.1371/journal.pbio.3003955.r002

**Authors:** Taylor Hart, PhD

8 Jun 2026

Dear Pelin,

Thank you for re-submitting your manuscript entitled "Deciphering the combinatorial expression and genetic regulatory mechanisms of Beats and Sides in the olfactory circuits of Drosophila" for consideration as a Research Article by PLOS Biology.

I have discussed your revised manuscript with the Academic Editor who advised on the earlier version of your manuscript, and we appreciate that the revisions have strengthened the study. We have decided to send the latest version of the manuscript out for further peer review, aiming to recruit reviewers from the original panel.

Because this manuscript is treated as a new submission in our system, before we can send it to reviewers, we need you to complete the submission again by providing the metadata that is required for full assessment. To this end, please login to Editorial Manager where you will find the paper in the 'Submissions Needing Revisions' folder on your homepage. Please click 'Revise Submission' from the Action Links and complete all additional questions in the submission questionnaire.

Once your full submission is complete, your paper will undergo a series of checks in preparation for peer review. After your manuscript has passed the checks it will be sent out for review. To provide the metadata for your submission, please Login to Editorial Manager (https://www.editorialmanager.com/pbiology) within two working days, i.e. by Jun 10 2026 11:59PM.

During the process of completing your manuscript submission, you will be invited to opt-in to posting your pre-review manuscript as a bioRxiv preprint. Visit http://journals.plos.org/plosbiology/s/preprints for full details. If you consent to posting your current manuscript as a preprint, please upload a single Preprint PDF.

Feel free to email us at plosbiology@plos.org if you have any queries relating to your submission.

Kind regards,

Taylor

Taylor Hart, PhD,

Associate Editor

PLOS Biology

thart@plos.org

# Author comment: Author response to Decision Letter 1

**DOI:** 10.1371/journal.pbio.3003955.r003

12 Jun 2026

Attachment Submitted filename: Response_Duan2026_BeatSideORNs_PlosBio_revision_v1.pdf

# Peer review: Decision Letter 1

**DOI:** 10.1371/journal.pbio.3003955.r004

**Authors:** Taylor Hart, PhD

9 Jul 2026

Dear Pelin,

Thank you for your patience while we considered your revised manuscript "Deciphering the combinatorial expression and genetic regulatory mechanisms of Beats and Sides in the olfactory circuits of Drosophila" for publication as a Research Article at PLOS Biology. This revised version of your manuscript has been evaluated by the PLOS Biology editors, the Academic Editor, and two of the original reviewers.

Based on the reviews and our discussion with the Academic Editor, we are likely to accept this manuscript for publication. We also discussed the point from Reviewer 2 about whether this manuscript would fit better into the Methods and Resources category, but we ultimately decided to keep it as a Research Article. Please also make sure to address the following data and other policy-related requests.

IMPORTANT: Please ensure that your next revision addresses the following points:

-------------------------

**Title:

We would like to tweak the title to bring it closer to our stylistic preference, which requires an active verb. We have a couple of alternative suggestions. Is one of these acceptable to you?

Beat- and Side-family cell-surface molecules are expressed combinatorially within olfactory circuit neurons in Drosophila

Or

Partner neurons in the fly olfactory circuit express specific combinations of Beat and Side genes

**Financial disclosure statement:

Please add links to the funding agencies in the Financial Disclosure statement in the manuscript details.

**Data:

Thank you for providing the underlying data. We require a bit more to meet our requirements, which include showing the numerical data as they relate directly to the figure plots. While I see that you include data related to Figures 2, 3, 4, 5, S10, S11, S12, we would specifically need to see the data used to generate the following panels:

2C

3C

4J

S3E

S5BC

S7D

S8BC

S10BC

S11CE

Could you please add this, as appropriate, and also include citations in all of the relevant main and supplementary figure legends indicating where these data can be found (e.g. “The data underlying this Figure can be found in S1 Data”)?

Can you please also tweak the naming scheme for these to match our style? In other words, "Data S1" should become "S1 Data" and have the filename "S1_Data.xlsx". As Table S1 and Table S3 also appear to contain source data, these should also be named following the same scheme (S3 Data, S4 Data, etc. ).

In the manuscript file description of the supporting documents, Data S1 is listed twice (one of these should become S2 Data).

**Figures:

Please upload the figure and supplementary figure files separately (the latter as Supporting Information).

**Phylogenetic trees:

If the phylogenetic trees in the paper were produced by you, please provide the tree files (or provide relevant references).

**Code availability:

I see that you plan to upload your custom code to GitHub.

Per journal policy, we require that you make it available without restrictions. Please ensure that the code is sufficiently well documented and reusable, and that your Data Statement in the Editorial Manager submission system accurately describes where your code can be found. Please also ensure that you choose a license for your code and include a Readme file.

Please note that we cannot accept sole deposition of code in GitHub, as this could be changed after publication. However, you can archive this version of your publicly available GitHub code to Zenodo. Once you do this, it will generate a DOI number, which you will need to provide in the Data Accessibility Statement (you are welcome to also provide the GitHub access information). See the process for doing this here: https://docs.github.com/en/repositories/archiving-a-github-repository/referencing-and-citing-content

**Data not shown:

Please note that per journal policy, we do not allow the mention of "data not shown", "personal communication", "manuscript in preparation" or other references to data that is not publicly available or contained within this manuscript. Please either remove mention of these data or provide figures presenting the results and the data underlying the figure(s).

-------------------------

As you address these items, please take this last chance to review your reference list to ensure that it is complete and correct. If you have cited papers that have been retracted, please include the rationale for doing so in the manuscript text, or remove these references and replace them with relevant current references. Any changes to the reference list should be mentioned in the cover letter that accompanies your revised manuscript.

In addition to these revisions, you may need to complete some formatting changes, which you will receive in a follow up email. A member of our team will be in touch with a set of requests shortly. If you do not receive a separate email within a few days, please assume that checks have been completed, and no additional changes are required.

We expect to receive your revised manuscript within two weeks.

To submit your revision, please go to https://www.editorialmanager.com/pbiology/ and log in as an Author. Click the link labelled 'Submissions Needing Revision' to find your submission record. Your revised submission must include the following:

- a cover letter that should detail your responses to any editorial requests, if applicable, and whether changes have been made to the reference list

- a Response to Reviewers file that provides a detailed response to the reviewers' comments (if applicable, if not applicable please do not delete your existing 'Response to Reviewers' file.)

- a track-changes file indicating any changes that you have made to the manuscript.

NOTE: If Supporting Information files are included with your article, note that these are not copyedited and will be published as they are submitted. Please ensure that these files are legible and of high quality (at least 300 dpi) in an easily accessible file format. For this reason, please be aware that any references listed in an SI file will not be indexed. For more information, see our Supporting Information guidelines:

https://journals.plos.org/plosbiology/s/supporting-information

*Published Peer Review History*

Please note that you may have the opportunity to make the peer review history publicly available. The record will include editor decision letters (with reviews) and your responses to reviewer comments. If eligible, we will contact you to opt in or out. Please see here for more details:

https://plos.org/published-peer-review-history/

*Press*

Should you, your institution's press office or the journal office choose to press release your paper, please ensure you have opted out of Early Article Posting on the submission form. We ask that you notify us as soon as possible if you or your institution is planning to press release the article.

*Protocols deposition*

To enhance the reproducibility of your results, we recommend that if applicable you deposit your laboratory protocols in protocols.io, where a protocol can be assigned its own identifier (DOI) such that it can be cited independently in the future. Additionally, PLOS ONE offers an option for publishing peer-reviewed Lab Protocol articles, which describe protocols hosted on protocols.io. Read more information on sharing protocols at https://plos.org/protocols?utm_medium=editorial-email&utm_source=authorletters&utm_campaign=protocols

Please do not hesitate to contact me should you have any questions.

Sincerely,

Taylor

Taylor Hart, PhD,

Associate Editor

thart@plos.org

PLOS Biology

Reviewer remarks:

Reviewer #1: The authors have done excellent work in revising the manuscript titled "Deciphering the combinatorial expression and genetic regulatory mechanisms of Beats and Sides in the olfactory circuits of Drosophila" (PBIOLOGY-D-26-01668_R1)! The response to my comments was thorough and reflected meticulous attention to details. There might be multiple reasons why single gene and even double gene knockdown did not show strong phenotypes. Future research could use conditional knockouts when tools are available. For now, the revised manuscript has substantially enhanced its scientific rigor and is recommended for publication.

Reviewer #2: This revised manuscript reflects an honest and hard working attempt by the authors to improve the paper based on the recommendations of three constructive reviewers. The authors should be lauded for the seriousness and robustness of their response.

That said, the mechanistic insight remains dull. This is likely a combination of bad luck, but also of somewhat superficial level of analysis. For example, pan-ORN, pan-PN KD (or over expression) analyses could be problematic in this sort of setting where every axon is testing its neighborhood. Attenuating one molecule from the entire system might not be sufficient to induce a phenotype. But taking it out from specific cells, based on educated guesses - for example KD/KO of beat1a in ORNs that project to DM1 or VA2 while KD/KO of Side IV from the respective PNs (I hope I'm getting the data from figure 4 right). Likewise, single cell over expression experiments would have been more informative in my opinion. Additionally, real loss of function experiments, using mutants rather than RNAi lines, is going to provide you with more conclusive take home messages. In today's technology, generating CRISPR-Cas9 mutants should not be an issue - at least for a selected group of genes.

I am not recommending more experiments because this is already a gigantic manuscript with lots of data. If such a category exists, I think this is an excellent resource paper.

# Author comment: Author response to Decision Letter 2

**DOI:** 10.1371/journal.pbio.3003955.r005

30 Jul 2026

Attachment Submitted filename: Response_Duan2026_BeatSideORNs_PlosBio_revision_v1_auresp_2.pdf

# Editor report: Decision Letter 2

**DOI:** 10.1371/journal.pbio.3003955.r006

**Authors:** Taylor Hart, PhD

3 Aug 2026

Dear Pelin,

Thank you for the submission of your revised Research Article "Beat- and Side-family cell-surface molecules are expressed combinatorially in the partner neurons of the olfactory circuit in Drosophila" for publication in PLOS Biology. I appreciate your thorough responses to our requests throughout the evaluation process. On behalf of my colleagues and the Academic Editor, Bing Ye, I am very happy to say that we can in principle accept your exciting study for publication.

You may possibly still need to address some formatting and reporting issues. If any remain, these will be detailed in an email you should receive within 2-3 business days from our colleagues in the journal operations team; no action is required from you until then. Please note that we will not be able to formally accept your manuscript and schedule it for publication until you have completed any requested changes.

Please take a minute to log into Editorial Manager at http://www.editorialmanager.com/pbiology/, click the "Update My Information" link at the top of the page, and update your user information to ensure an efficient production process.

PRESS

We frequently collaborate with press offices. If your institution or institutions have a press office, please notify them about your upcoming paper at this point, to enable them to help maximise its impact. If the press office is planning to promote your findings, we would be grateful if they could coordinate with biologypress@plos.org. If you have previously opted in to the early version process, we ask that you notify us immediately of any press plans so that we may opt out on your behalf.

We also ask that you take this opportunity to read our Embargo Policy regarding the discussion, promotion and media coverage of work that is yet to be published by PLOS. As your manuscript is not yet published, it is bound by the conditions of our Embargo Policy. Please be aware that this policy is in place both to ensure that any press coverage of your article is fully substantiated and to provide a direct link between such coverage and the published work. For full details of our Embargo Policy, please visit http://www.plos.org/about/media-inquiries/embargo-policy/.

Thank you again for choosing PLOS Biology for publication and supporting Open Access publishing. We look forward to publishing your study.

Sincerely,

Taylor

Taylor Hart, PhD,

Associate Editor

PLOS Biology

thart@plos.org
